# The Oldest Jurassic Dinosaur: A Basal Neotheropod from the Hettangian of Great Britain

**DOI:** 10.1371/journal.pone.0145713

**Published:** 2016-01-20

**Authors:** David M. Martill, Steven U. Vidovic, Cindy Howells, John R. Nudds

**Affiliations:** 1 School of Earth and Environmental Sciences, University of Portsmouth, Portsmouth, United Kingdom; 2 Department of Natural Sciences, Amgueddfa Cymru - National Museum Wales, Cardiff, United Kingdom; 3 School of Earth, Atmospheric and Environmental Sciences, University of Manchester, Manchester, United Kingdom; State Natural History Museum, GERMANY

## Abstract

Approximately 40% of a skeleton including cranial and postcranial remains representing a new genus and species of basal neotheropod dinosaur is described. It was collected from fallen blocks from a sea cliff that exposes Late Triassic and Early Jurassic marine and quasi marine strata on the south Wales coast near the city of Cardiff. Matrix comparisons indicate that the specimen is from the lithological Jurassic part of the sequence, below the first occurrence of the index ammonite *Psiloceras planorbis* and above the last occurrence of the Rhaetian conodont *Chirodella verecunda*. Associated fauna of echinoderms and bivalves indicate that the specimen had drifted out to sea, presumably from the nearby Welsh Massif and associated islands (St David’s Archipelago). Its occurrence close to the base of the Blue Lias Formation (Lower Jurassic, Hettangian) makes it the oldest known Jurassic dinosaur and it represents the first dinosaur skeleton from the Jurassic of Wales. A cladistic analysis indicates basal neotheropodan affinities, but the specimen retains plesiomorphic characters which it shares with *Tawa* and *Daemonosaurus*.

## Introduction

**Fig 1:**
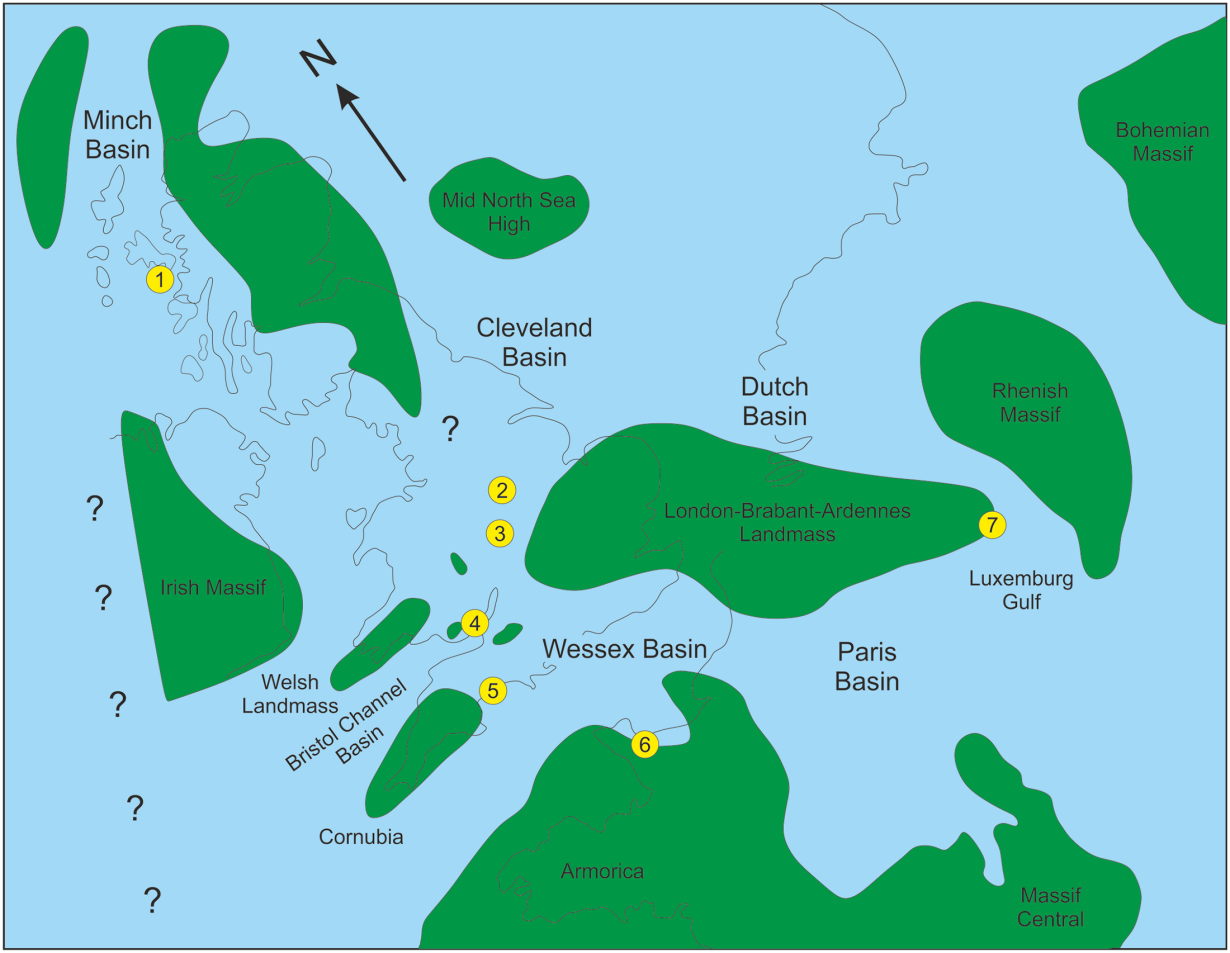
Outline palaeogeography of northwest Europe with Hettangian theropod dinosaur localities indicated. 1, Isle of Skye, Scotland; 2, Barrow upon Soar, Leicestershire, 3, Wilmcote, Warwickshire; 4, Lavernock Point, Glamorgan, Wales; 5, Lyme Regis/Charmouth, Dorset; 6, Airel, France; 7, Brouch, Luxembourg. Based in part on [6] with data from [1–5].

Theropod dinosaurs are extremely rare in the Lower Jurassic and most reports are of only fragmentary remains[1–6]. This rarity results in a considerable gap in our knowledge of these animals at a time when, indications are, theropods were diversifying rapidly. In Europe Early Jurassic theropods are reported from the Hettangian of Scotland[1], England[2,3,4], France[5] and Belgium[6], but all of these occurrences are of fragmentary material, isolated bones, or a few associated elements, with most of it non-diagnostic at generic level (Fig 1). Oddly, most have been obtained from marine or marginal marine strata. A few examples of Lower Jurassic theropods are known from elsewhere; they include the abelisaurid *Berberosaurus* from the Toarcian of Morocco[7], *Cryolophosaurus* from the Sinemurian-Pliensbachian of Antarctica[8], *Syntarsus* from the Hettangian-Pliensbachian of South Africa and Zimbabwe [9,10], *Podekosaurus* from the Pliensbachian to Toarcian of Massachussetts[11], and from Arizona *Dilophosaurus* from the Hettangian[12] and *Segisaurus* from the Pliensbachian to Toarcian[13], all of which are Neotheropoda. The presence of the derived therizinosaurid *Eshanosaurus* in the Lower Jurassic of China, tentatively dated as Hettangian[14], implies that many other higher theropod taxa should also be represented in the Lower Jurassic, if the dating of *Eshanosaurus* proves correct. However, a Hettangian age for *Eshanosaurus* contrasts with all other therizinosaurid occurrences[15], although the paucity of theropod remains in the Lower Jurassic globally may be the explanation for the disparity. It is imperative that fragmentary remains such as those of *Eshanosaurus* are identified correctly and reliably dated before any firm conclusions are drawn from their seemingly anachronistic occurrences. Two theropods have been named from the English Hettangian, *Sarcosaurus woodi* Andrews, 1921[4] from Barrow upon Soar, Leicestershire, based on an isolated pelvis, vertebra and proximal femur (BMNH 4840/1) and *Sarcosaurus andrewsi* Huene, 1932 [16] based on a partial tibia (NHMUK R3542) (see also ref [3]).

### Dinosaurs in Wales

Despite extensive outcrops of early Mesozoic strata in south Wales, including units of vertebrate-bearing, non-marine and marginal marine facies, the remains of dinosaurs in Wales are exceedingly rare. Excluding the Triassic (Norian) age footprints of Barry, Glamorgan[17], most Welsh dinosaur records are of isolated bones and teeth from fissure fill deposits where poorly dated sequences infill palaeokarst developed in Carboniferous age limestones[18,19] or from rémanie deposits, such as the so-called Rhaetian bone bed at or near the base of the Westbury Formation[20,21]. A single, large theropod left dentary with teeth was recovered from loose blocks of probable littoral sandstones from Stormy Down, Glamorgan,[22] and named *Zanclodon cambrensis* (see [21]) while *Thecodontosaurus caducus* was from the Late Triassic of Bonvilston, Glamorgan, south Wales[18]. This specimen was later given its own genus, *Pantydraco*[23].

**Fig 2:**
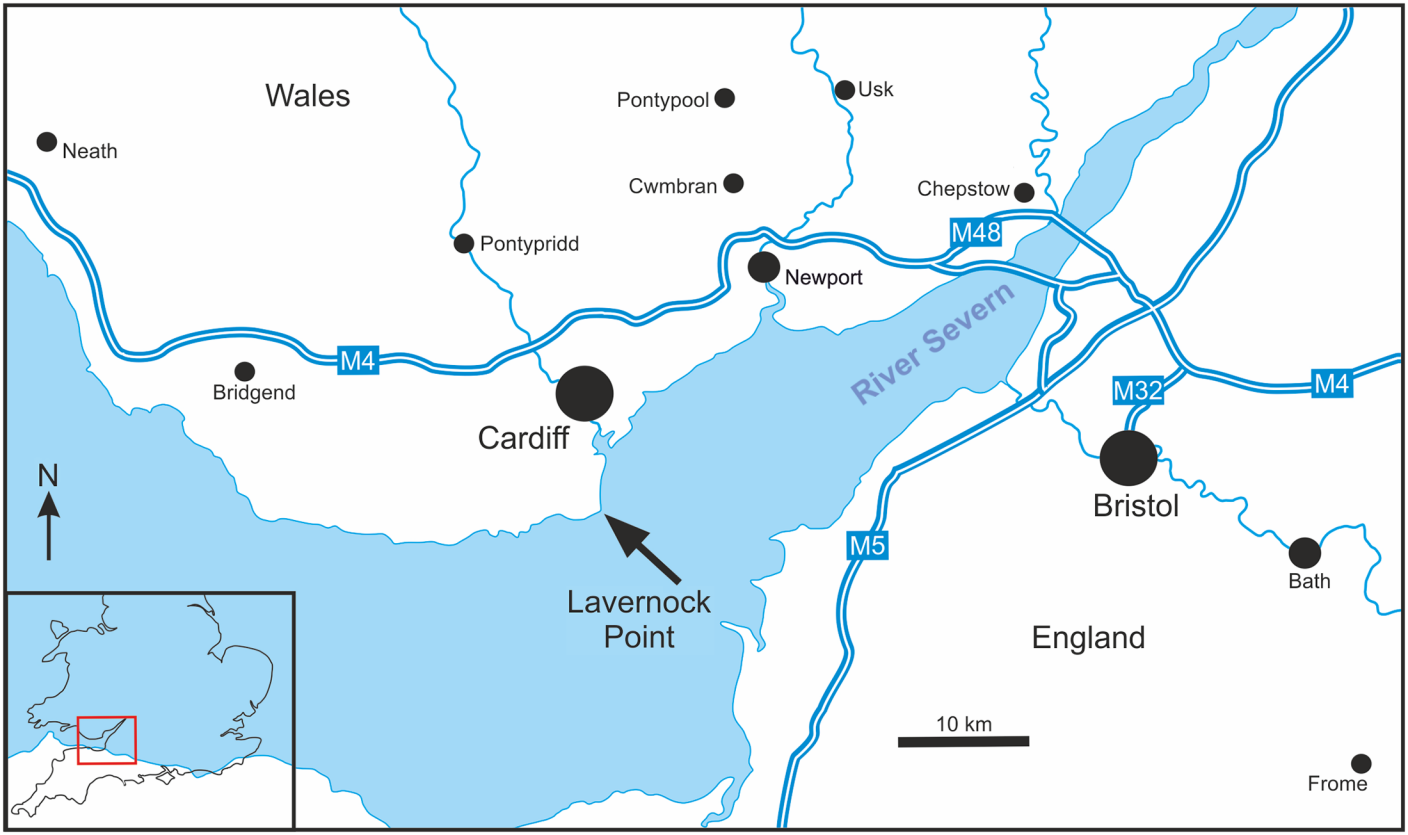
Outline map showing Lavernock Point on the Welsh side of the Severn Estuary. This site exposes one of the finest sections through the Triassic-Jurassic boundary in Europe, and yields important zone fossils in both the Triassic and Jurassic strata allowing for accurate dating.

**Fig 3:**
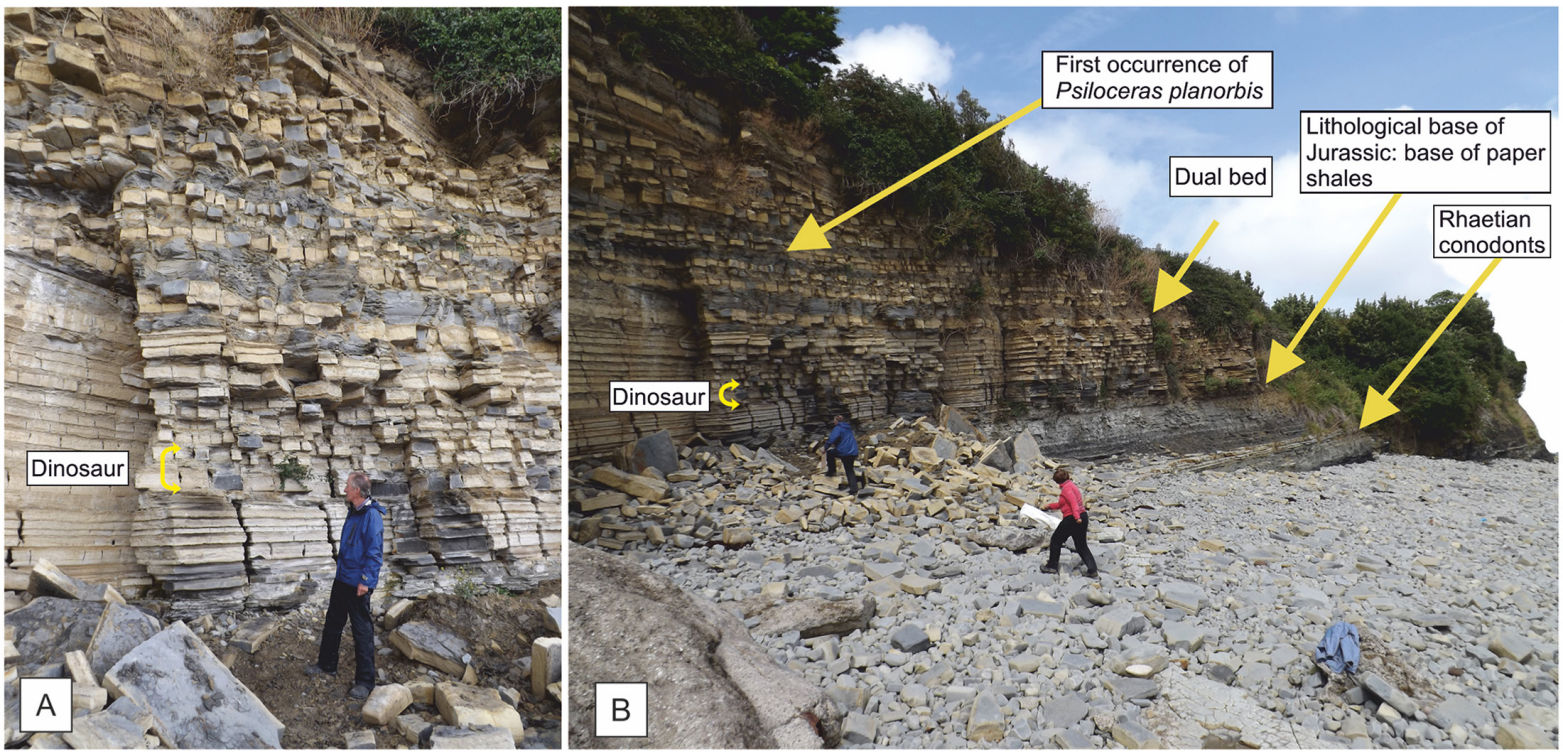
Cliff exposures at Lavernock Point, National Grid reference ST 187681. (A) The base of the Jurassic showing a series of alternating limestones and mudstones. The new specimen is most likely from the higher of two limestones indicated in yellow that contain a thin shelly horizon, but it was recovered from the fallen blocks in front of the exposure. The upper of the two matches most closely the bed thickness of the slabs with the dinosaur bones. (B) The same beds in stratigraphic context with the highest occurrence of conodonts, the lithological base of the Jurassic and the first occurrence of the ammonite *Psiloceras planorbis* indicated.

**Fig 4:**
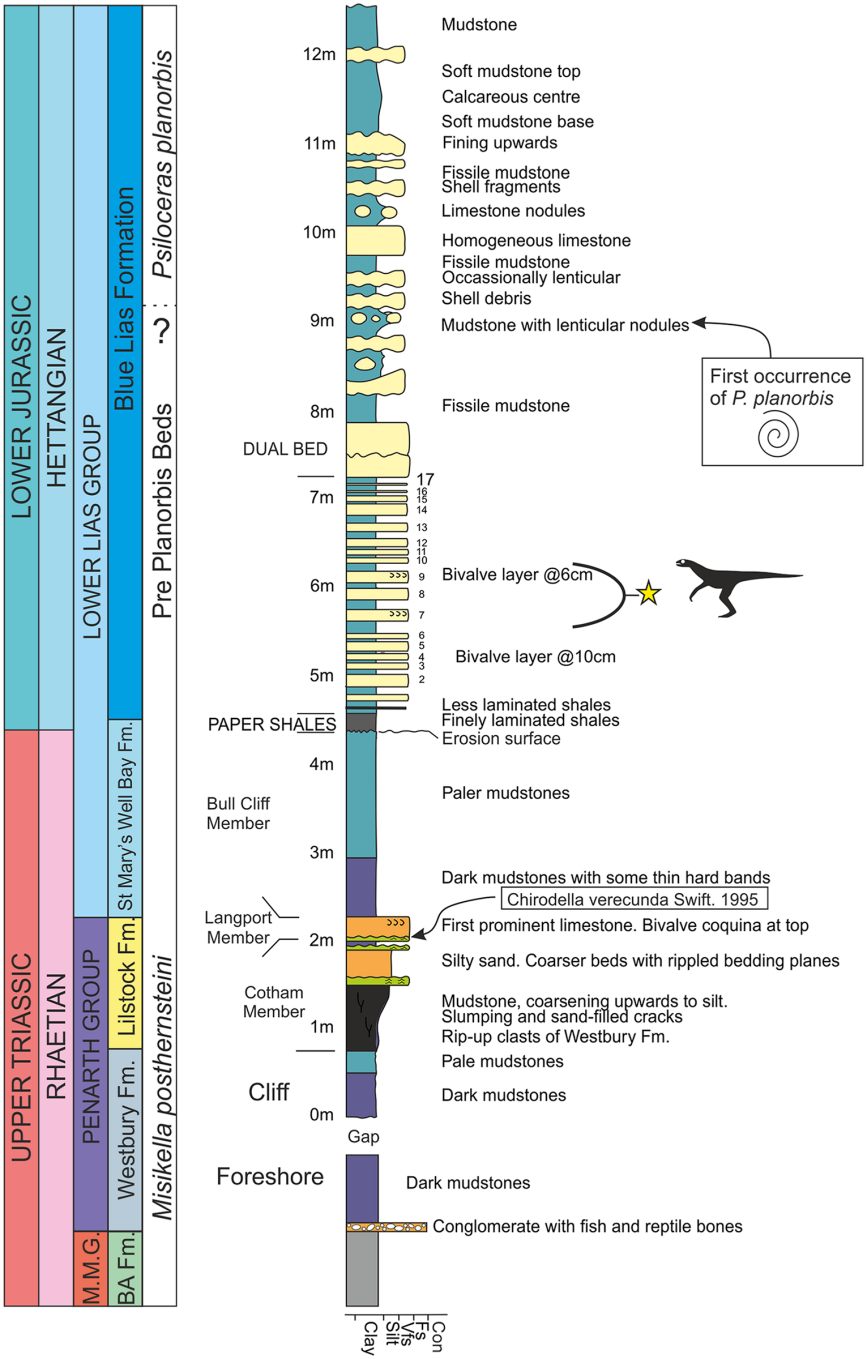
Stratigraphic log for Lavernock Point. The lithological boundary of the base of the Jurassic is the base of the Paper Shales horizon at 4.5 metres. Many British stratigraphers have historically used the first occurrence of the ammonite *Psiloceras planorbis* to indicate the base of the Jurassic, but elsewhere in Europe two other psiloceratacean ammonites appear before *P*. *planorbis*. The thin Langport Member at 2.2 m has been dated as Rhaetian by Swift (1995) using conodonts. The new theropod comes from one of the limestones at the 6 m interval (bed 7 or 9).

**Fig 5:**
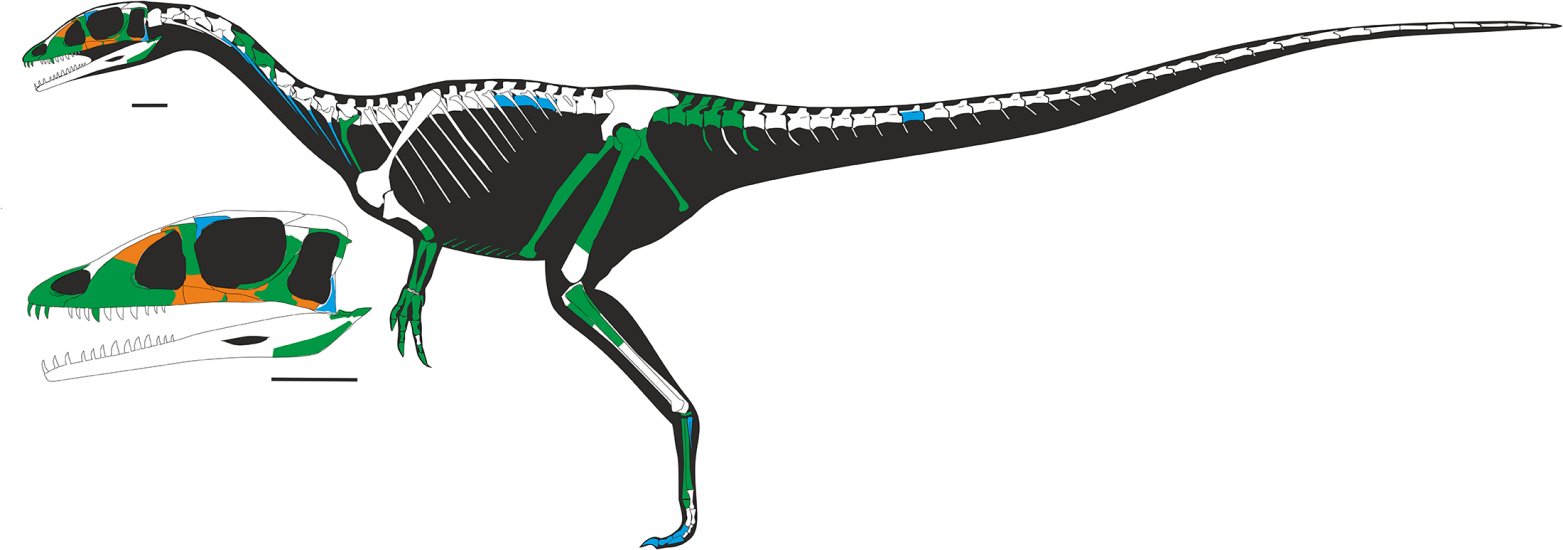
Skeleton outline of *Dracoraptor hanigani*. Bones highlighted green for present, orange for external moulds and blue for tentatively identified bones. Many unidentified or uncertain elements have been omitted.

Here we describe a partial disarticulated skeleton, including the skull, of a new genus and species of theropod dinosaur from well-dated marine strata of the southeast coast of Wales (Figs 2,3 and 4). It represents the most complete theropod from Wales, and one of the most complete from the European Lower Jurassic. The specimen is deposited in Amgueddfa Cymru—National Museum Wales, Cardiff, accession number NMW 2015.5G.1–2015.5G.11 with individual blocks and skeletal elements numbered as in Table 1 (Fig 5).

**Table 1:** Accessioned blocks of the new theropod from Lavernock Point, south Wales.

Description	Accession number
Skull block with counter slab	NMW 2015.5G.1a and NMW 2015.5G.1b
Hind limb, ischium, pubes, caudal vertebrae and chevron block with counter slab	NMW 2015.5G.2a and NMW 2015.5G.2b
Forearm and manus block, with fibula	NMW 2015.5G.3
Right pes block	NMW 2015.5G.4
Caudal vertebra and process	NMW 2015.5G.5
Loose cervical vertebra	NMW 2015.5G.6
Loose tibia fragment	NMW 2015.5G.7
Small block with fragmentary? rib	NMW 2015.5G.8
Casts of skull blocks	NMW 2015.5G.9-10
Cast of leg block	NMW 2015.5G.11
Left pes blocks	NMW 2015.10G.1a/b

*Museum abbreviations used*. NMW, Amgueddfa Cymru—National Museum Wales, Cardiff; NHMUK, Natural History Museum, London.

## Materials and Methods

### Basic collecting, preparation and repair

The specimen was discovered in March 2014 and ‘rescue’ collected from a minor cliff collapse of well bedded limestones and mudstones at Lavernock Point (Fig 4) by Mr Nick Hanigan and Mr Rob Hanigan. The larger blocks were dried over two weeks under damp newspaper to prevent rapid shrinkage and cracking of a thin mudstone veneer on the limestone surface. The various slabs were cleaned and prepared mechanically to expose bones using sodium bicarbonate abrasive powder, and were X-rayed and CT scanned at the Faculty of Clinical Radiology, University of Manchester. Small broken bones were repaired using superglue. Slabs with bones represented as external moulds were cast in a silicon compound, replicated in fibreglass and resin, and painted to restore missing bones known to have been present and presumed lost to the sea. Extensive searching failed to discover the missing bones on the foreshore. There exists the possibility that some of the specimen remains in the cliff, but repeated careful examination of the cliff face failed to reveal any exposed bones.

### Cladistic analysis

To establish the phylogenetic position of the new specimen it was analysed in a cladistic analysis comprised of 366 characters and 46 taxa, 18 of which lie outside of Dinosauria. The analysis used the 338 characters and coding of You et al.[24], formerly of Ezcurra and Brusatte[25], with *Daemonosaurus* included using the coding of Sues et al.[26]. It was possible to atomize 25 of the compound characters (sensu [27]) in the character list of Ezcurra and Brusatte[25], resulting in a further 28 characters. An additional compound character was reduced from three character states to two.

To demonstrate the effect of atomizing compound characters an experiment using jacknife procedures was conducted. The jacknife analysis was manually performed using Microsoft Excel to generate random characters for omission in each replicate of the procedure. For each replicate of the jacknife procedure the number of characters utilised and the resulting tree-lengths were recorded. The jacknife procedure was implemented on the original matrix of You et al.[24] and the matrix presented here, minus *Daemonosaurus* and the new theropod. Two bivariate plots were generated from the recorded data; one analysing difference in tree-length (total matrix tree-length minus jacknife replicate tree-length) *vs*. the number of characters removed; and the other analysing tree-length *vs*. the number of characters analysed in each replicate. It was predicted that the former plot would demonstrate the same rate of tree-length decay as characters are removed, whilst the latter would demonstrate an overall difference in tree-length between the two matrices. In the event of the predictions being correct, the two graphs in tandem would demonstrate a difference between the matrices that is a result of a change in character conflict, as opposed to the quantity of characters.

The ensemble retention index (RI) which is independent of character number did not change between the original analysis of You et al.[24] and the new analysis, due to the coding being exactly the same. Likewise, a plot of the difference in tree-length vs. the number of characters removed demonstrated the same rate of tree-length decay for both matrices. However, the larger, new matrix was only 15 steps longer despite an additional 25 characters. Additionally, the difference in relative tree-lengths taken from the regression lines of the jacknife plot (tree-length vs. number of characters) was approximately 60 steps less for the larger matrix. Therefore, the jacknife demonstrates the benefits of atomizing compound characters.

The final analysis was run in TNT using a ‘new technology’ search. Implied weights were used to adjust character weights during the analysis, in order to combat any coding errors or character conflict that was not resolved by atomizing compound characters. The resulting MPT stored to the computer’s RAM was further analysed with a TBR swapping algorithm in TNT’s ‘traditional’ search function to resolve the maximum MPTs possible. A Bootstrap analysis using 100 replicates in a ‘new technology’ search was also run. Absolute values were stored on the bootstrap tree and it can be found, along with further details on all the procedures discussed here in the supporting information (S1 File).

### Nomenclatural Acts

The electronic edition of this article conforms to the requirements of the amended International Code of Zoological Nomenclature, and hence the new names contained herein are available under that Code from the electronic edition of this article. This published work and the nomenclatural acts it contains have been registered in ZooBank, the online registration system for the ICZN. The ZooBank LSIDs (Life Science Identifiers) can be resolved and the associated information viewed through any standard web browser by appending the LSID to the prefix “http://zoobank.org/”. The LSID for this publication is: urn:lsid:zoobank.org:pub:2146600F-0A2D-4815-8361-C009021B3513. The electronic edition of this work was published in a journal with an ISSN, and has been archived and is available from the following digital repositories: PubMed Central, LOCKSS and the University of Portsmouth’s library, Pure.

### Stratigraphic Position and Age

Although not found *in situ*, the new dinosaur has been constrained on lithological grounds to one of two limestone horizons within the Blue Lias Formation of the Lower Lias Group (Fig 4), and specifically within the lower Bull Cliff Member (stratigraphic terminology follows that of Simms[28]) in the Bristol Channel Basin. Unfortunately, this horizon is the one part of the sequence for which a precise date remains contentious as it lies below the first occurrence of the ammonite *Psiloceras planorbis* (Sowerby, 1824)[29] historically used by British stratigraphers to define the base of the Jurassic[30], and above the last occurrence of a marker conodont for the Triassic, namely *Chirodella verecunda* Swift, 1995[31]. As a matter of pragmatism, these strata have traditionally been regarded as Late Rhaetian age and are informally called the Pre-Planorbis Beds [28,32,33,34]. The new dinosaur comes from a stratum that is ~3 metres below the first occurrence of *P*. *planorbis* and ~4 metres above the last confirmed conodont occurrence.

At Lavernock Point there is a distinctive lithological change at the base of the Blue Lias Formation that historically was used by some workers as a lithological definition of the base of the Jurassic[35] and is employed here.

In European and North American sections across the Rhaetian/Hettangian (Triassic/Jurassic) boundary the first appearance of a psiloceratacean ammonite is marked by the occurrence of *Psilocerus erugatum*, followed by *P*. *imitans* and *P*. *antecedans*, all of which are used to define subzones beneath the first occurrence of *P*. *planorbis*. In Austria *P*. *spelae tirolicum*, *P*. cf. *pacificum* and *P*. ex gr. *P*. *tilmanni* occur in strata below *P*. *planorbis*[36] while in Belgium and northeast Russia, *Primapsiloceras primulum* has been reported from strata below *P*. *planorbis*[37,38]. These earliest Jurassic subzones and other psilocerataceans have not been detected in the Lavernock Point sequence of strata. However, ammonites predating *Psiloceras planorbis* have been found in the basal Blue Lias Formation of Somerset, just 22 km south of Lavernock Point[34]. These include examples of *P*. cf. *erugatum* and *Neophyllites* sp. demonstrating that the basal subzone ammonites of the Jurassic did reach the British Isles.

The absence of these very early Jurassic forms at Lavernock is either a reflection of a facies dependency of the earliest Jurassic ammonites that excludes them from nearer shore waters, or (more likely) lack of preservation. In this latter respect it is worth noting that *P*. *planorbis* fossils of higher beds in the Lavernock Point Blue Lias Formation are preserved as compressed periostracal films with no aragonite preservation nor calcitic shell replacement, while aragonitic bivalves are entirely missing from the Bull Cliff Member. The fossil assemblage is thus a chemical lag dominated by calcitic fossils (echinoderms, ostreacean and pectenacean bivalves), and phosphatic vertebrate remains.

The question remains, should the new dinosaur be attributed to the very latest Triassic, or the very earliest Jurassic? Geochemical considerations may help resolve the precise stratigraphic age of the dinosaur bearing stratum. A number of studies have located isotopic excursions at or near the Triassic-Jurassic boundary (e.g. [39] for sequences in Hungary; [40] for Austria; [41] for Nevada, USA) and the British Isles[42,43]. A study at Lavernock Point[42], found a δ^13^C_org_ negative anomaly at the top of the Langport Member of the Triassic Penarth Group, while an extensive, integrated analysis of a section on the south side of the Severn Estuary at St Audries’ Bay, Somerset also detected a massive δ^13^C_org_ negative isotope excursion commencing just above the last conodont occurrences, but before the first ammonite occurrence[43]. This negative excursion has also been detected elsewhere, and in a section in New York State, USA, Ward et al.[44] proposed this excursion to define the base of the Jurassic.

### Conodont ages

Conodonts have been recorded from the Late Triassic Langport Member of the Lilstock Formation (Penarth Group) at Lavernock Point, some 4 metres beneath the dinosaur discovery site and 7 metres below the first occurrence of *Psiloceras planorbis*[44]. Examples of *Chirodella verecunda* Swift, 1995[31] have been collected from Bed 3 of Ivimey Cook[45] (see also [46,31]) and are indicative of the *Misikella postehernsteini* (Kozur and Mock, 1974)[47] conodont biozone. This conodont biozone is indicative of the Rhaetian *Paracochloceras suessi* to *Cochloceras marshi* ammonite biozones of the Tethyan Late Triassic[48,49]. The zonal conodont indicator *Misikella postehernsteini* has not been reported with certainty from Lavernock Point, but it has been reported from lithologically equivalent strata in the English Midlands[31].

#### Associated invertebrate fauna

The new theropod skeleton is associated with a variety of marine shelly invertebrate fauna on the same bedding surface including stem ossicles resembling, but not identical to, those of the crinoid *Hispidocrinus* sp. and portions of test and spines of the regular echinoid cf. *Diademopsis* sp. In addition associated bivalve fragments seem to be referable to *Liostrea* (grey, laminated), *Plagiostoma* (dark grey-black, shiny) *Pseudolimea*, cf *Oxytoma* sp. and juvenile examples of cf. *Antiquilima* (grey, ribbed). This assemblage is regarded as representing a Jurassic fauna.

### Latest Triassic or earliest Jurassic?

The ‘Golden Spike’ [GSSP] for the base of the Jurassic is at Kuhjoch in the Austrian Northern Calcareous Alps[36]. At this locality the base of the Jurassic is defined at the first occurrence of the psiloceratacean ammonite *Psiloceras spelae tirolicum*. This ammonite species occurs approximately 5 metres above the isotopic excursion proposed as the base of the Jurassic[50,51]. Hillebrandt et al.[36] in commenting on the T/J boundary in Britain stated that “the boundary should be expected to occur in the lowest few meters of the Blue Lias Formation”. It is fair to say that in the British Isles, it may never be possible to define the T/J boundary, and that a pragmatic approach may be adopted as a matter of convenience. The minor-non-sequence beneath the basal Paper Shale horizon at the base of the Blue Lias Formation would seem the most logical place to put the Triassic/Jurassic Boundary at Lavernock Point.

Considering that the new dinosaur occurs at a stratigraphic position above the δ^13^C_org_ isotope anomaly; that it occurs above a clear lithological boundary representing a distinct facies change from shallow to deepening marine conditions; that it is associated with a Jurassic type shelly fauna, and that pollen analyses of nearby sections are of Jurassic type[50,52], the new dinosaur should be regarded as being of Early Hettangian age. Thus it most likely belongs to the *P*. *erugatum* subzone of the *P*. *tilmanni* Zone and is dated at 201.3 ± 0.2 million years old[36].

## Results

### Systematic Palaeontology

Dinosauria Owen, 1842[53]

Saurischia Seeley, 1888[54]

Theropoda Marsh, 1881[55]

Neotheropoda Bakker, 1986[56]

*Dracoraptor* gen. nov.

urn:lsid:zoobank.org:act: DFD000B9-D42B-495D-B807-DCBA3B2C3745

*Dracoraptor hanigani* sp. nov.

urn:lsid:zoobank.org:act: 21D0AF91-5893-47D6-9C3B-EF985487C60B

#### Holotype

NMW 2015.5G.1–2015.5G.11 is a disarticulated, but associated partial skeleton with elements of the skull, including both premaxillae, both maxillae, some teeth, a lacrimal, partial jugal, post orbital, squamosal, fragmentary lower jaws and a possible hyoid, and postcranial skeleton including two cervical vertebrae, posterior elements of the vertebral column (lumbar and caudal vertebrae), distal forelimb, ischium and pubis, hind limb with femur, and fragmentary tibia with proximal fibula. There are also cervical ribs, thoracic ribs and several unidentified fragments (Table 1, Fig 5).

#### Etymology

The genus name *Dracoraptor* is from Draco alluding to the dragon of Wales with raptor, meaning robber, a commonly employed suffix for theropod dinosaurs. The species name honours Nick and Rob Hanigan who discovered the skeleton and generously donated it to Amgueddfa Cymru-National Museum of Wales.

#### Locality and horizon

The new specimen was collected from several limestone and mudstone blocks among debris from a small rock fall at the base of the cliff on the east side of Lavernock Point (National Grid reference ST 187681). The cliff at this locality is ~7 m high and exposes the top of the Late Triassic Penarth Group on the east side, and the Blue Lias Formation of the Lower Lias Group forming the point. Structurally this is the southwest dipping limb of the Lavernock syncline in the Bristol Channel Basin (Figs 2–4). The cliff fall in which *Dracoraptor* was discovered comprises material restricted to the lowest two to three metres of the section and comprises debris mainly from the Bull Cliff Member of the Blue Lias Formation (see above for detailed discussion of the age of these strata). Lithological and bed thickness comparisons indicate that the dinosaur comes from either bed 7 or bed 9 (Fig 4), of the Bull Cliff Member, both of which contain a thin, but distinctive bed of broken calcitic shelly material[57].

#### Diagnosis

A basal neotheropod with the following autapomorphies and unique combination of plesiomorphies: Three teeth in the premaxilla, slender maxillary process of jugal, large narial opening with slender subnarial bar, anteriorly directed pubis considerably longer than ischium, and large dorsal process on distal tarsal IV.

#### Differential diagnoses relative to selected Norian/Rhaetian and Hettangian Neotheropoda

*Dracoraptor hanigani* can readily be distinguished from other basal neotheropods despite significant parts of the skeleton being missing or incomplete. *D*. *hanigani* differs from *Coelophysis* and *Syntarsus* in that the nasal process of the lacrimal is short. In *Coelophysis* and *Syntarsus* this process is considerably longer than the height (> × 2) of the lacrimal[58,59]. In *Camposaurus arizonensis* from the Norian of Arizona, USA, widely regarded as the oldest neotheropod[58,25], the distal fibula is fused to the distal tibia and to the fused astragalocalcaneum. Although this may be a feature of a mature individual, these elements are unfused in *Dracoraptor*, which is of similar size.

Comparisons with *Panguraptor* are not easy to make due to the very small size of *Panguraptor*. Nonetheless, from published figures[24] the maxilla of *Panguraptor* has three anteriorly located fenestra that distinguish it from most other Theropoda, including *Dracoraptor*. There are also differences in the relative size of the phalanges compared to the radius and ulna, which appear to be considerably longer in *Panguraptor*.

Relative to ‘*Syntarsus*’ *keyantakatae*, *D*. *hanigani* lacks the acute bend in the antero-dorsal margin of the maxilla for articulation with the premaxilla, and does not appear to have the proximally located fibular sulcus (Fig 5 of[10]).

*Lophostropheus airelensis* from the Hettangian of Normandy, France differs from *D*. *hanigani* in the form and complexity of the pleurocels of its cervical vertebrae[60]. However, the cervical vertebrae of *Liliensternus liliensterni* are quite similar to those of *D*. *hanigani*, but the ventral lamina of the centrum is well developed in both *Lophostropheus* and *Liliensternus*, but weakly developed or absent in *D*. *hanigani*.

Relative to *Tawa* from the Late Triassic of New Mexico, USA, *D*. *hanigani* has a similar lacrimal, but as in ‘*Syntarsus*’ *keyantakatae* it differs markedly in the form of the anterior margin of the premaxilla. Furthermore in *D*. *hanigani* the anterior border of the external nares is gently rounded whereas in *Tawa* this fenestra appears somewhat angular (Nesbitt et al. 2009)[61]. Selected measurements for *Dracoraptor* are given in Table 2.

**Table 2:** Selected measurements of skeletal elements of *Dracoraptor hangani*.

Skeletal element	Dimensions
Right premaxilla subnarial length	32 mm
Right premaxilla dorsal process length	39 mm
Left premaxilla dorsal process length	42 mm
Left composite premaxilla subnarial length	32 mm
Right maxilla preserved length	34 mm
Right maxilla maximum height	43 mm
Lacrimal depth	51 mm
Jugal length (impression)	57 mm
Jugal post orbital process height	31 mm
Jugal post orbital process length	33 mm
Jugal post orbital angle	123 degrees
Radius	71 mm
Ulna	73 mm
Fibula preserved length	105 mm
Femur preserved length	158 mm
Metatarsal II	102 mm
Metatarsal III	116 mm
Metatarsal IV	93 mm
Metatarsal V? preserved length	44 mm
Ungual	25 mm
Ischium shaft preserved length, to posterior margin of the acetabulum	129 mm
Estimated total length of pubis	212 mm
Pubis next to femur preserved length	182 mm
Pubis underlying femur preserved length	171 mm

## Description

### Skull

The skull is around 2/3 complete, but disarticulated. Most of the few associated teeth have fallen from their alveoli, but are closely associated with tooth-bearing elements. The anterior rostral bones, premaxillae and maxillae are associated, but not articulated. Some elements of the skull are preserved as external moulds, or have composite bone/mouldic preservation, notably jugal and lacrimal elements. Of the braincase only a supraoccipital has been confidently identified and only more caudal elements of the lower jaws have been identified.

#### Premaxilla

**Fig 6:**
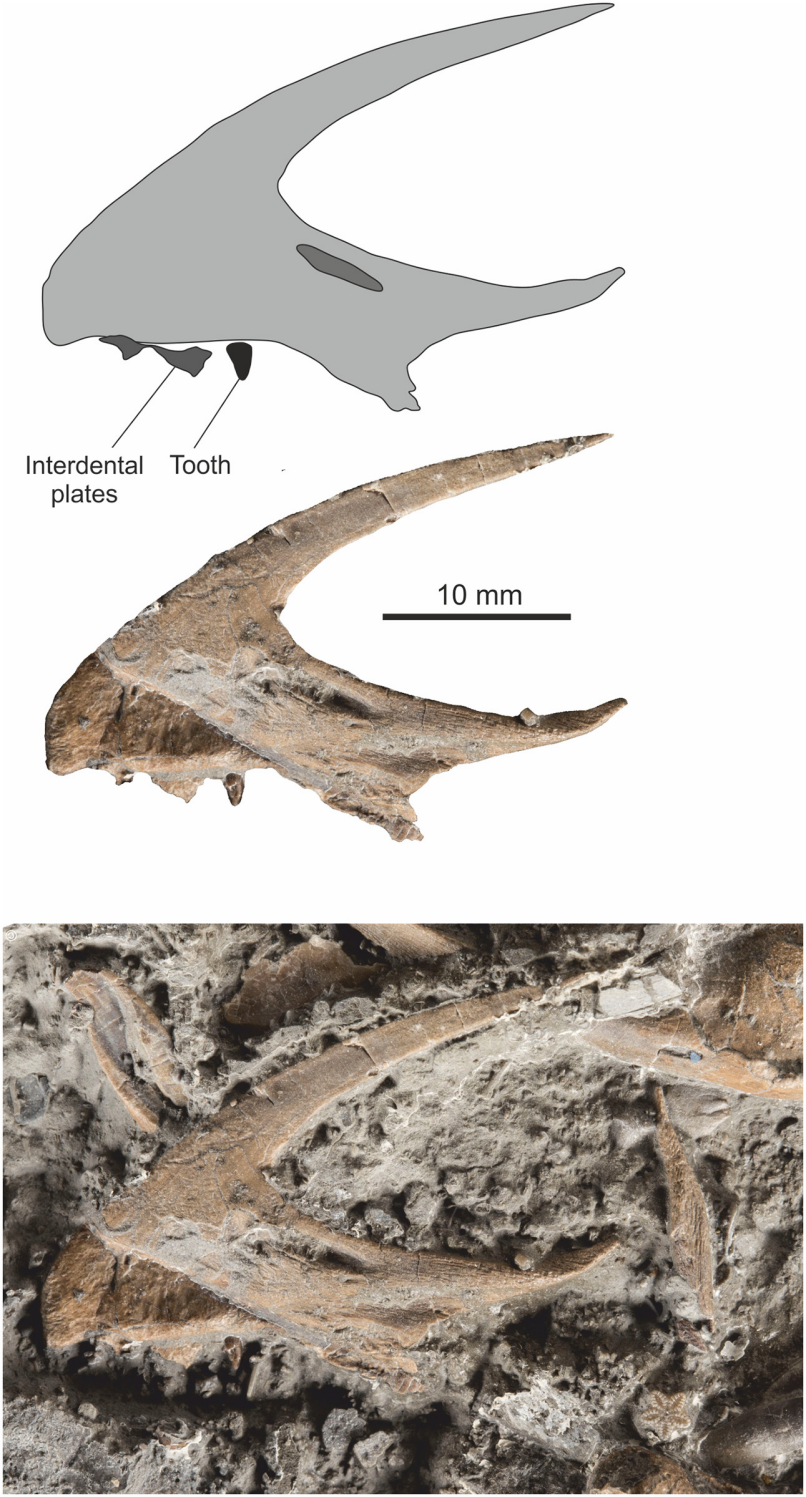
Right premaxilla in medial view. Top, simplified outline diagram highlighting the position of an elongate sub-narial vacuity. Middle, the premaxilla with matrix digitally removed. Bottom, premaxilla *in situ*.

**Fig 7:**
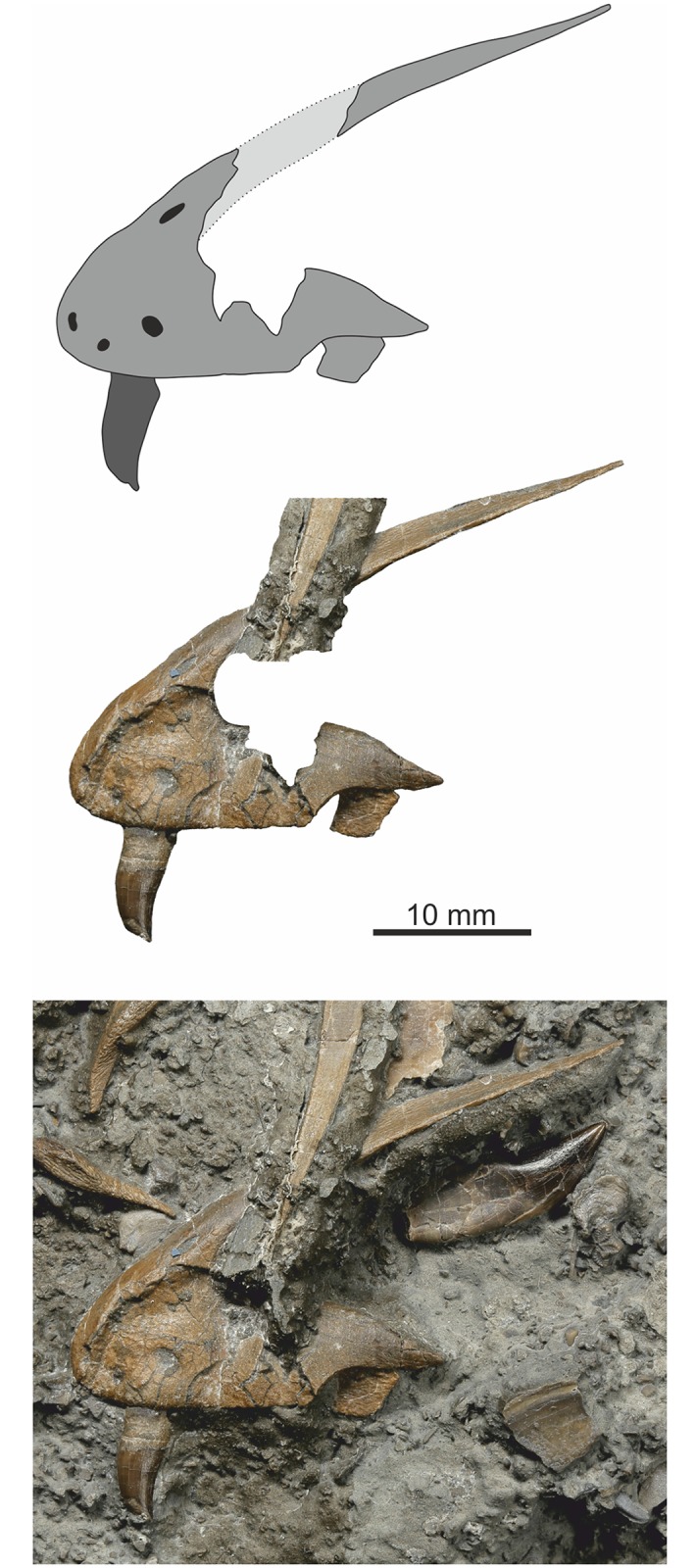
Left premaxilla with tooth in lateral view. Top, simplified outline diagram highlighting a series of? sensory openings at the tip of the element. Middle, the premaxilla and tooth with matrix digitally removed. Bottom, premaxilla *in situ*.

Both premaxillae are preserved and both are nearly complete with only small parts of the dorsocaudal or ventrocaudal rami missing. The left is exposed in lateral view while the right displays its medial surface. There are three tooth sockets in each premaxilla, but the teeth are generally absent. A partially erupted, caudally located replacement tooth is present in the right premaxilla (Fig 6) and a fully erupted tooth is present in alveolus two of the left premaxilla (Fig 7).

#### Maxilla

**Fig 8:**
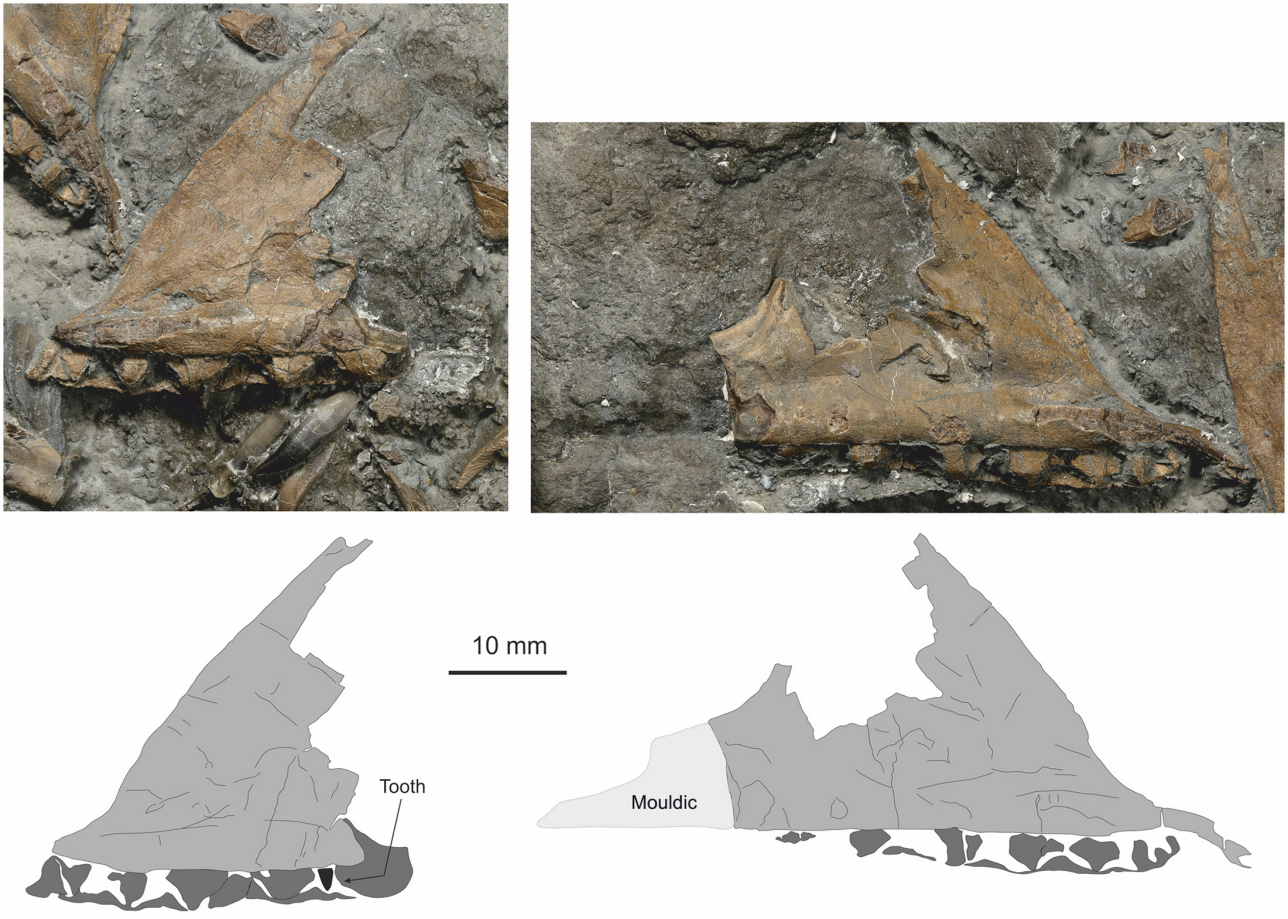
Left and right maxilla in medial view. Top left, incomplete right maxilla with dental shelf and interdental plates clearly visible. Top right incomplete left maxilla, but with caudal portion preserved as external mould. Bottom images simplified line drawings highlighting the interdental plates.

Both maxillae are preserved and are almost entire, missing just some of the thinner part of the cheek while the right element is missing some of the caudal region. No teeth are retained in the alveoli. Both elements are seen in lateral view and the interdental plates are well seen. The right element has five alveoli preserved while the left displays seven alveoli (Fig 8). Four teeth lie close to the dental border of the right maxilla.

#### Teeth

**Fig 9:**
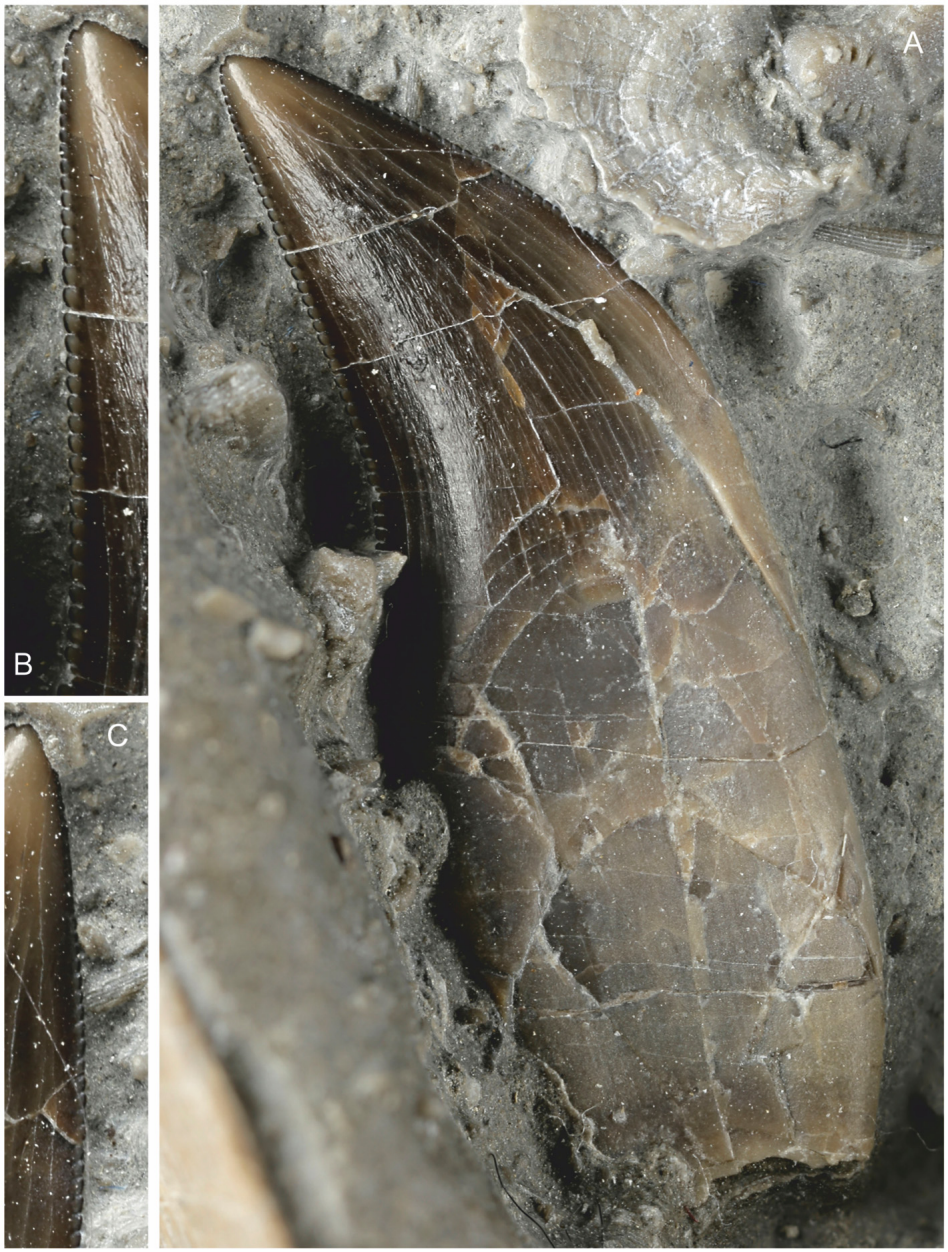
Tooth, assumed to be from right maxilla with which it lies adjacent. (A) Entire tooth with crown and root. Note that the tip is in good condition with minimal wear. (B) Serrations of the caudal margin. (C) Serrations of the cranial margin with a slightly damaged tip and some wear on the first few (app. 8–10) serrations. The morphology of the serrations on either side is distinctive.

At least 7 teeth are present in the assemblage. One is *in situ* in the left premaxilla (Fig 7), a replacement tooth is just visible in the right premaxilla, three closely associated complete teeth are adjacent to the dental border of the left maxilla while the root of another lies adjacent to these. A tooth is also associated with a portion of possible dentary. All of the teeth are caudally recurved, laterally compressed and serrated (Fig 9). The four teeth associated with the maxilla, and assumed to be maxillary teeth are at least 12 mm high from the root base to the crown tip, with the enamelled crown 5 mm high. The crown is serrated both on the cranial and caudal carina, but the serration denticles extend all the way to the crown-root junction only on the caudal carina. There are between 6 and 8 denticles per millimetre with denticles decreasing in size slightly towards the crown tip. The denticles are considerably worn on the cranial carina at the tip, and toward the root they fade out and the crown margin becomes smooth (Fig 9c).

#### Lacrimal

**Fig 10:**
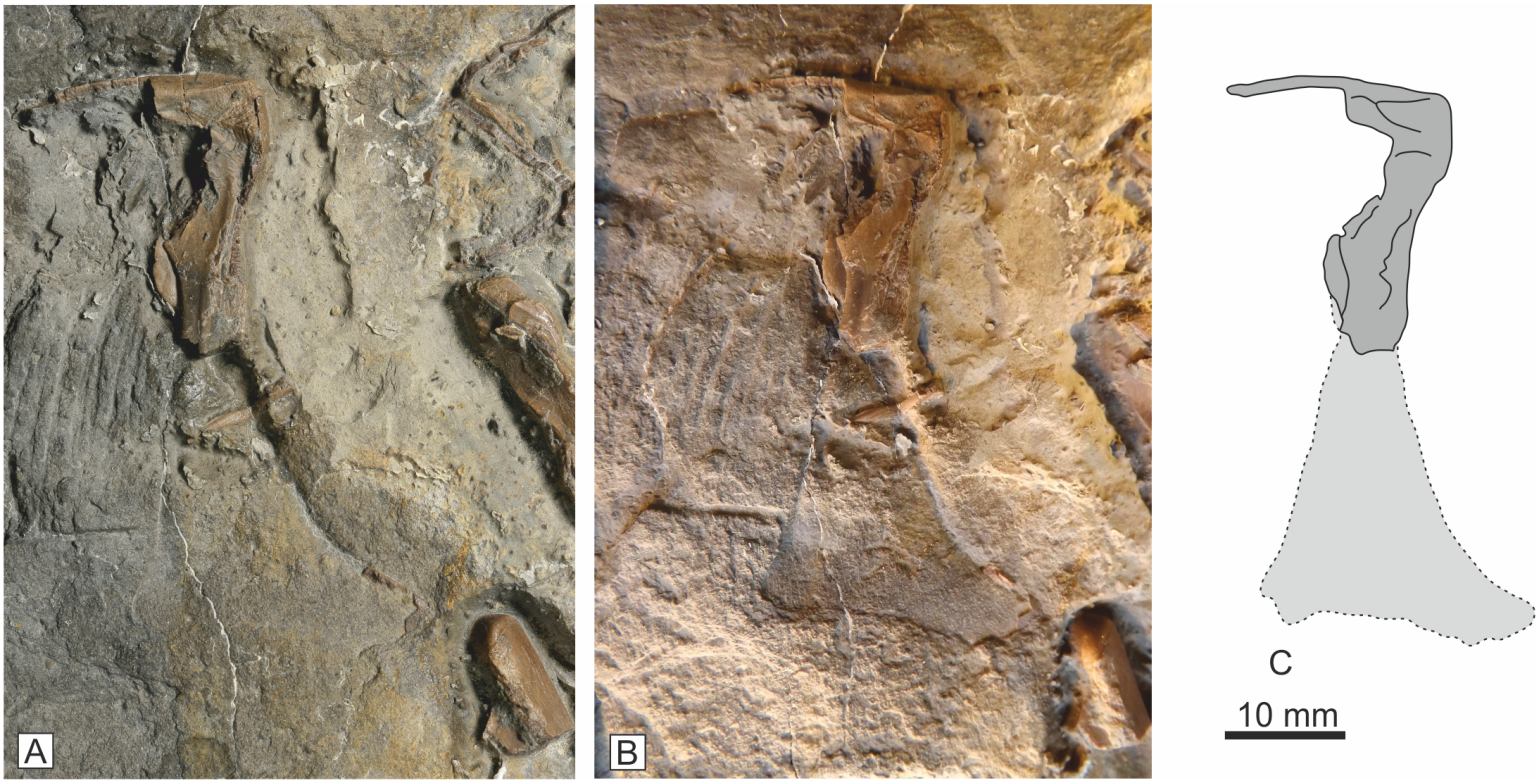
Lacrimal. This bone is missing its ventral half, but an excellent external mould shows the outline of the element. (A) The lacrimal with ‘flat’ light, (B) with very low angle light from NW, (C) and outline of lacrimal. Most probably there are small portions missing from the area to the top left.

A single lacrimal is present, but only its dorsal half is represented by bone, the ventral half preserved as an external mould (Fig 10). The preserved bone portion is approximately hour-glass shaped in lateral view with an antero-dorsal process approximately the same length as the basal margin that articulates with the jugal/maxilla.

#### Jugal

**Fig 11:**
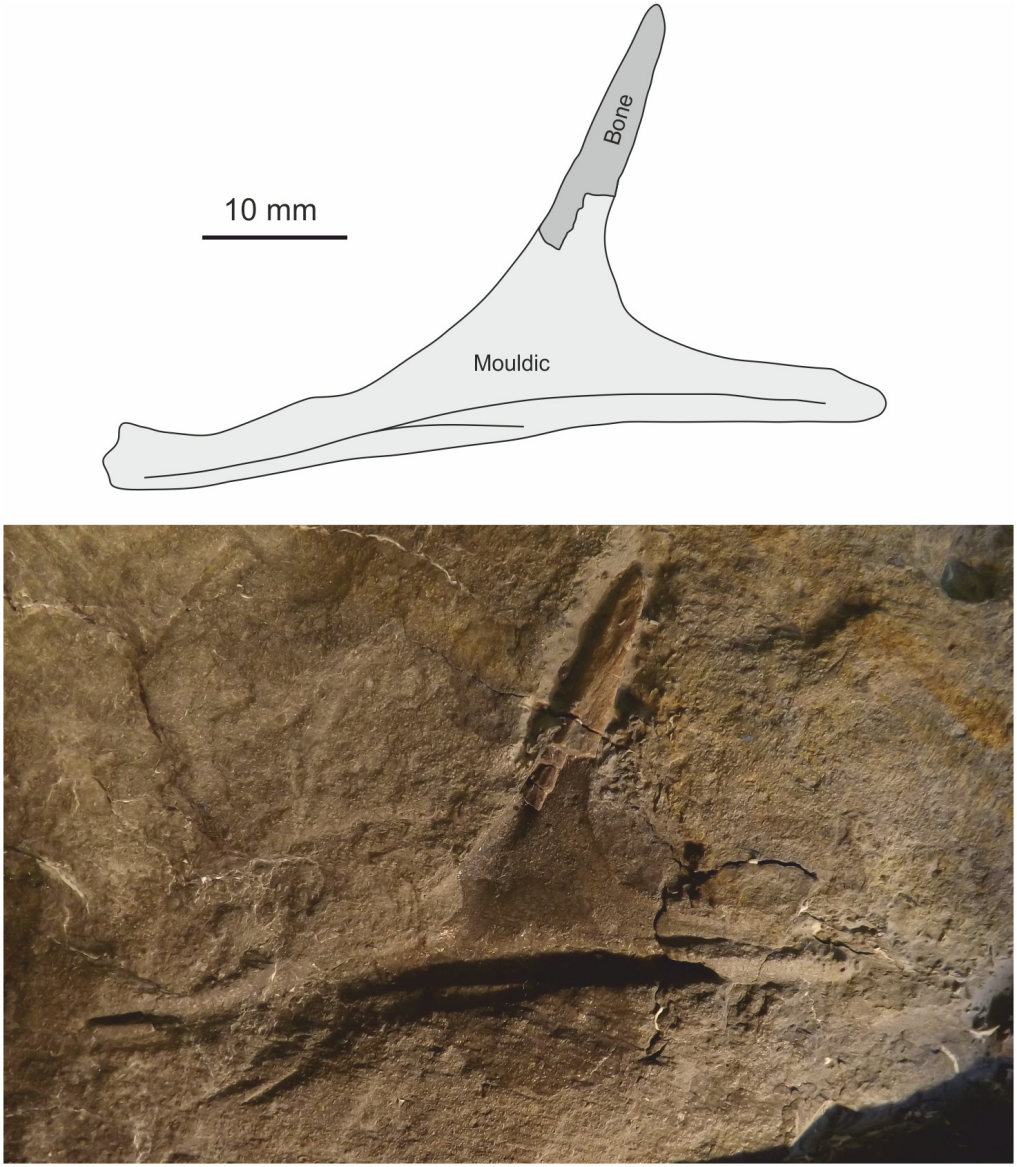
Jugal, A, probably from the left side. Only a small portion of the original bone is preserved in the dorsal process, but a distinctive mould (B) of the entire element is clearly visible in low angle light.

A single left jugal is preserved mostly as an external mould, but with some original bone in the postorbital process and anteriormost tip of the maxillary process (Fig 11). The bone is slender with a straight ventral border. The lacrimal process is directed dorso-caudally at an angle of 60° to the ventral border. There is an impression of a ridge and beneath it a groove slightly above the ventral margin and paralleling it. This groove and ridge extends cranially and caudally for much of the length of the preserved element, but evidence for its continuation caudally is missing. It probably represents the sutural surface for the maxilla.

#### Supraoccipital

**Fig 12:**
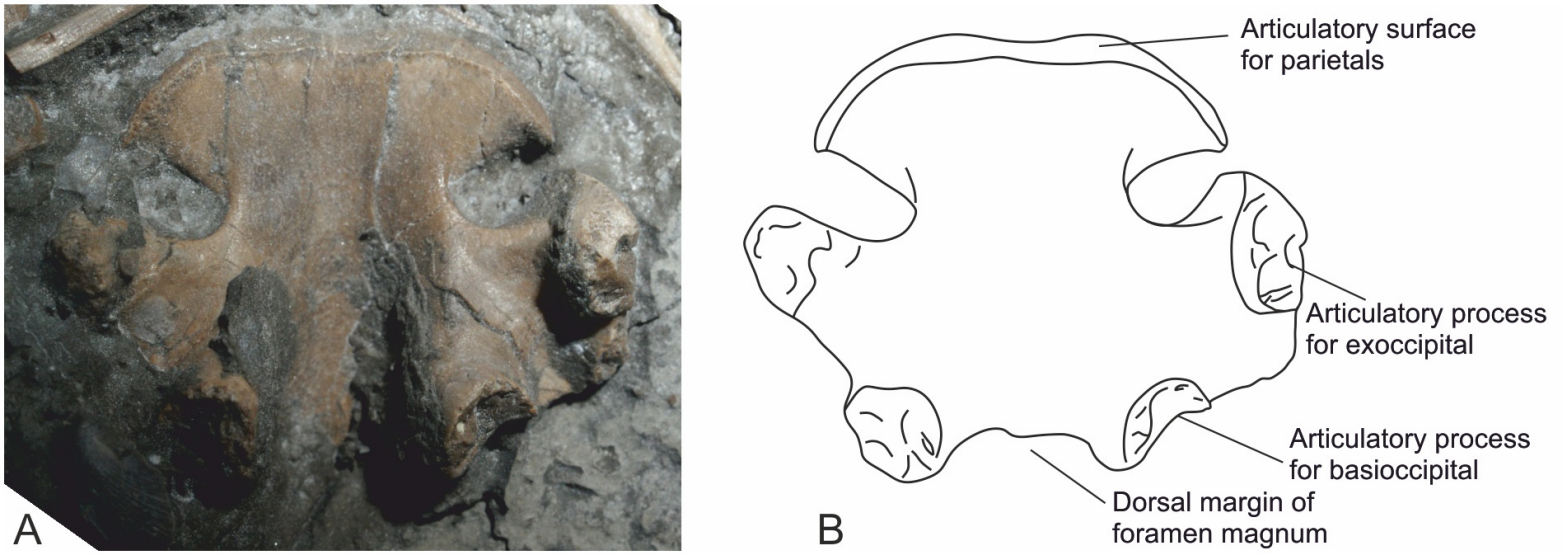
Supraoccipital in ventral view.

This element is seen in ventral view. It is well preserved with two clear processes for articulation with the exoccipitals (Fig 12). It is almost as long as it is broad, giving it a subquadrate outline. Caudally two processes uniting with the basioccipital project directly downwards, thus forming the lateral margins of the foramen magnum. The anterior border is arcuate with a slight concavity medially and is bordered by deep, paired antero-lateral notches.

#### Skull roof element

**Fig 13:**
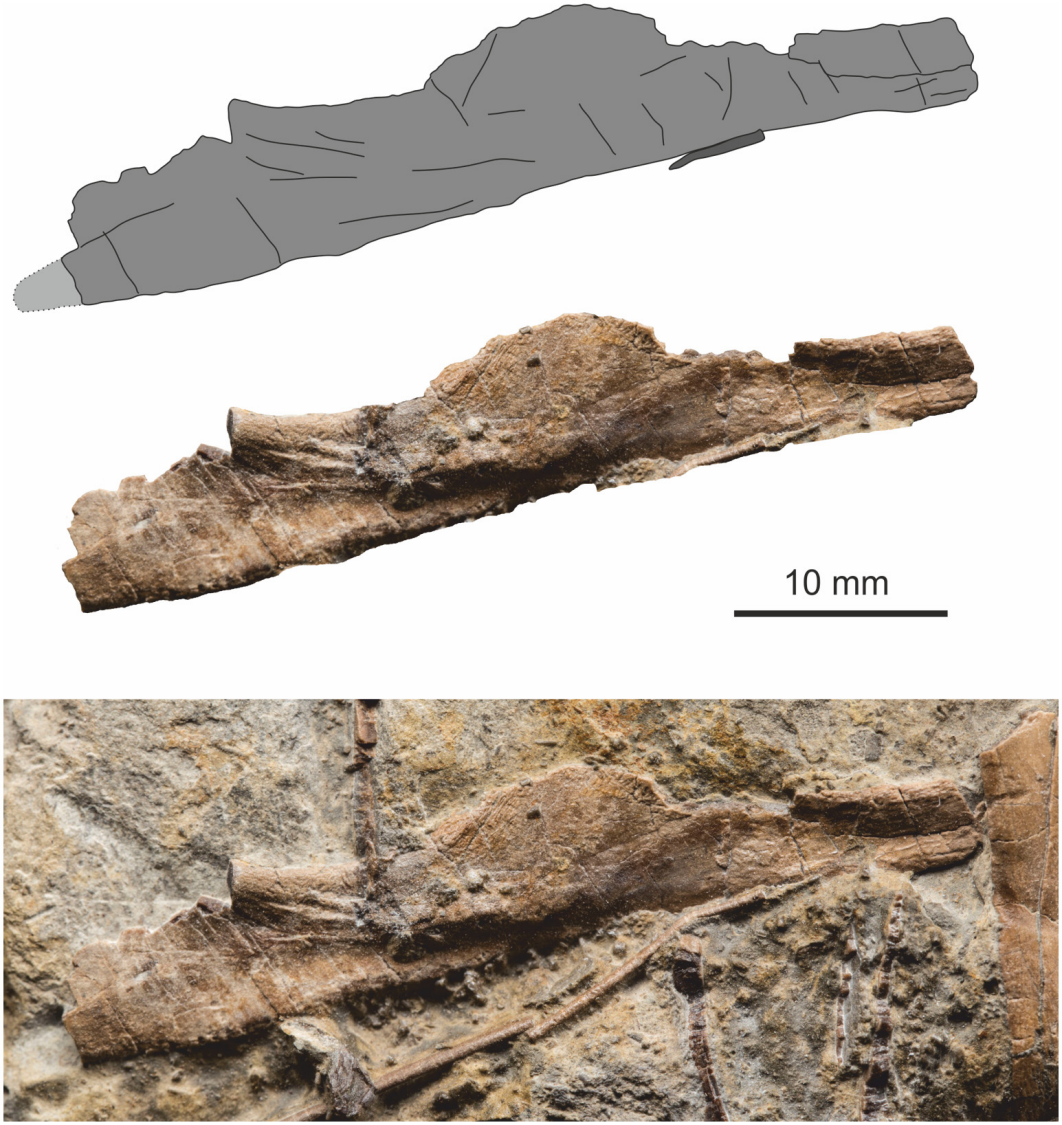
Uncertain element, possibly a nasal. This isolated element has a distinctive straight margin suggesting a median location in the skull. Unfortunately useful landmarks are absent.

A single elongate bone (Fig 13) with straight medial border is assumed to represent one of the paired elements of the skull roof. There are no obvious markers to confirm its identity, but a small lateral process with broken termination may be a caudal process as seen in the nasal of *Syntarsus*.

### Axial skeleton

Elements of the axial skeleton preserved include cervical vertebrae and ribs, posterior thoracic vertebrae, anterior thoracic ribs, anterior caudal vertebrae, chevrons and gastralia. Most are disarticulated, and most vertebral centra are separated from their neural arches. Some ribs are associated with vertebrae, but none are articulated with them. At least five centra are in loose articulation close to the pelvic elements.

#### Cervical vertebrae and ribs

**Fig 14:**
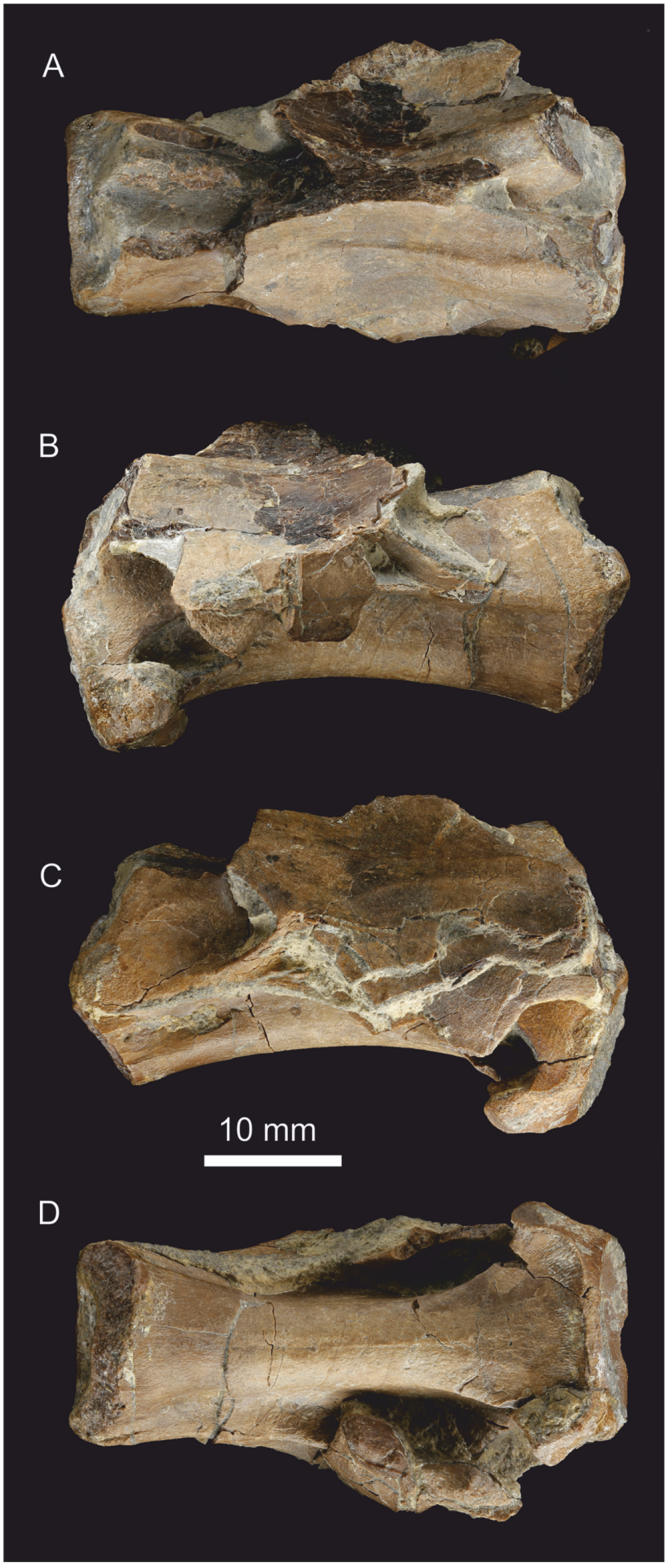
Anterior cervical vertebra. Position in neck uncertain, possibly 3 or 4. (A) Dorsal view, (B) left lateral view, (C) right lateral view, (D) ventral view. The neural spine is damaged, but a fresh break suggests it was present prior to collection.

**Fig 15:**
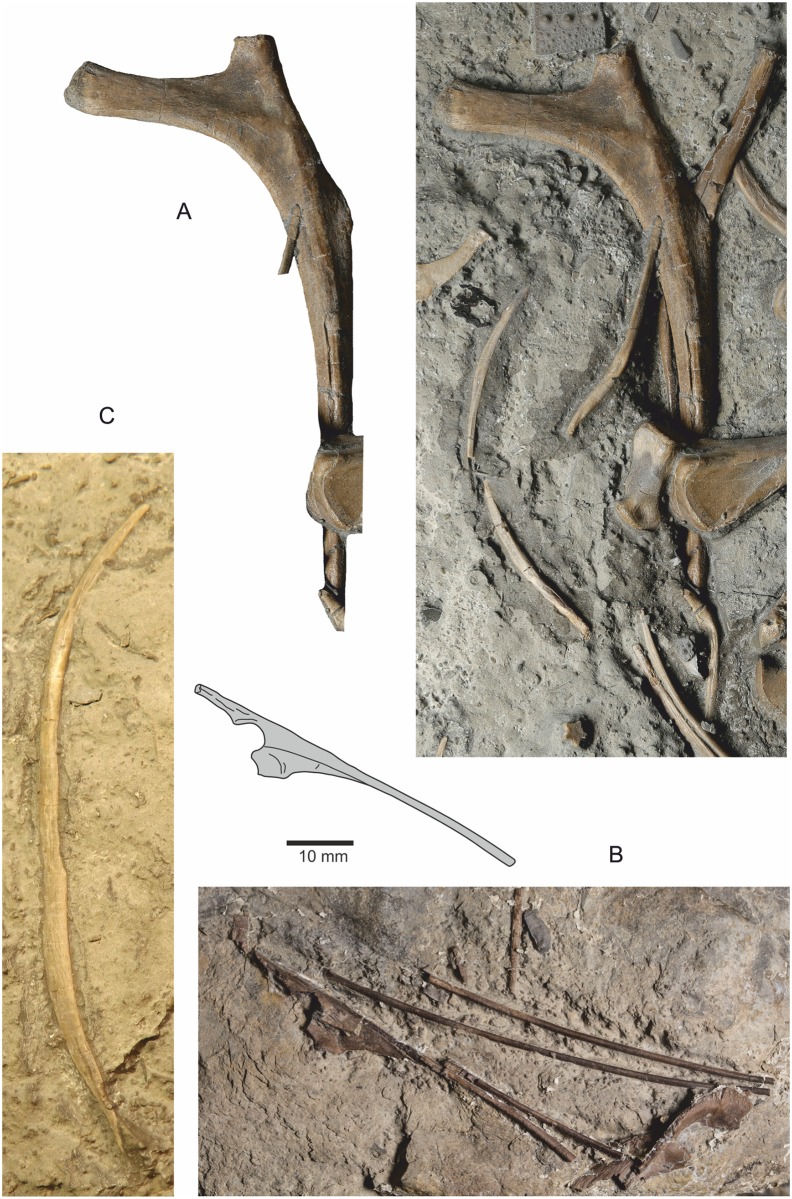
Cervical and thoracic ribs. (A) Left anterior thoracic rib in cranial view. (B) Three partial cervical ribs, and almost complete cervical rib and a fragment of a fifth cervical rib. This closely associated group of ribs is not adjacent to any cervical vertebra. (C) Gastral rib from median row.

Two cervical vertebrae (Fig 14) are preserved along with a group of four cervical ribs (Fig 15) and fragments of thin, straight bones that may also be cervical ribs. The vertebrae have elongate centra, are opisthocoelous with long, low neural spines, although these are broken caudally. Processes for articulation of the cervical ribs are located cranially with the inferior process situated on the centrum. The dorsal process projects from the neural arch. The ventral margin of the centrum is gently arched while the centrum itself is ‘pinched’ mid corpus. A pleurocoel is present on the lateral margins of the centrum cranially. In caudal view the centrum has a subquadrate outline.

#### Thoracic or posterior cervical ribs

Although no certain thoracic vertebrae are present in the assemblage, there is a single thoracic rib (Fig 15), and a smaller, probable thoracic rib. The ventral process for articulating with the vertebrae is elongate while the dorsal process is much shorter. The dorso-lateral surface of the probable thoracic rib is conspicuously straight or slightly concave before curving convexly ventrally.

#### Caudal vertebrae

**Fig 16:**
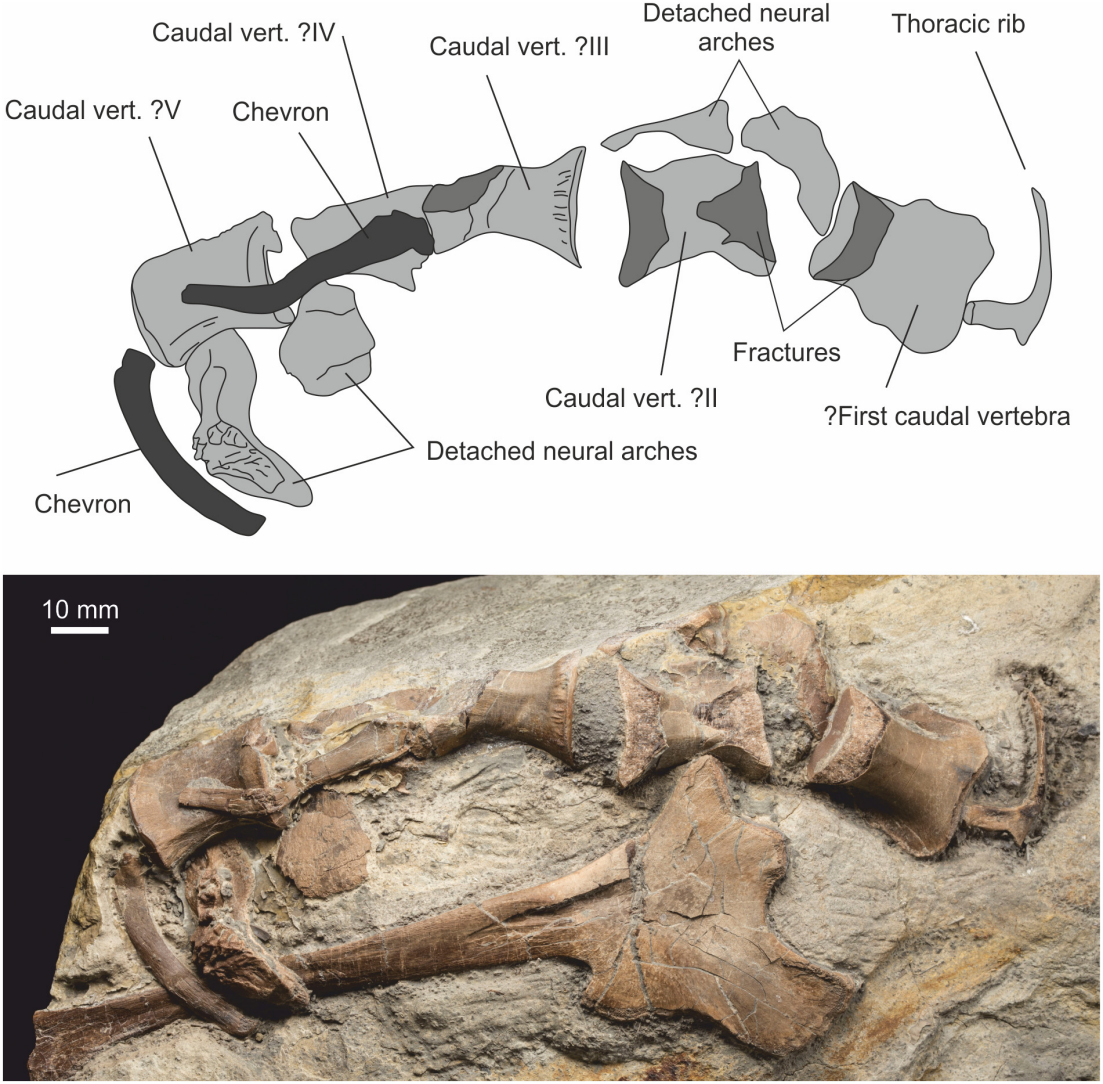
Anterior caudal vertebrae in ventral aspect with detached neural arches. Also within view are the first two chevrons, the ischium and a partial thoracic rib.

**Fig 17:**
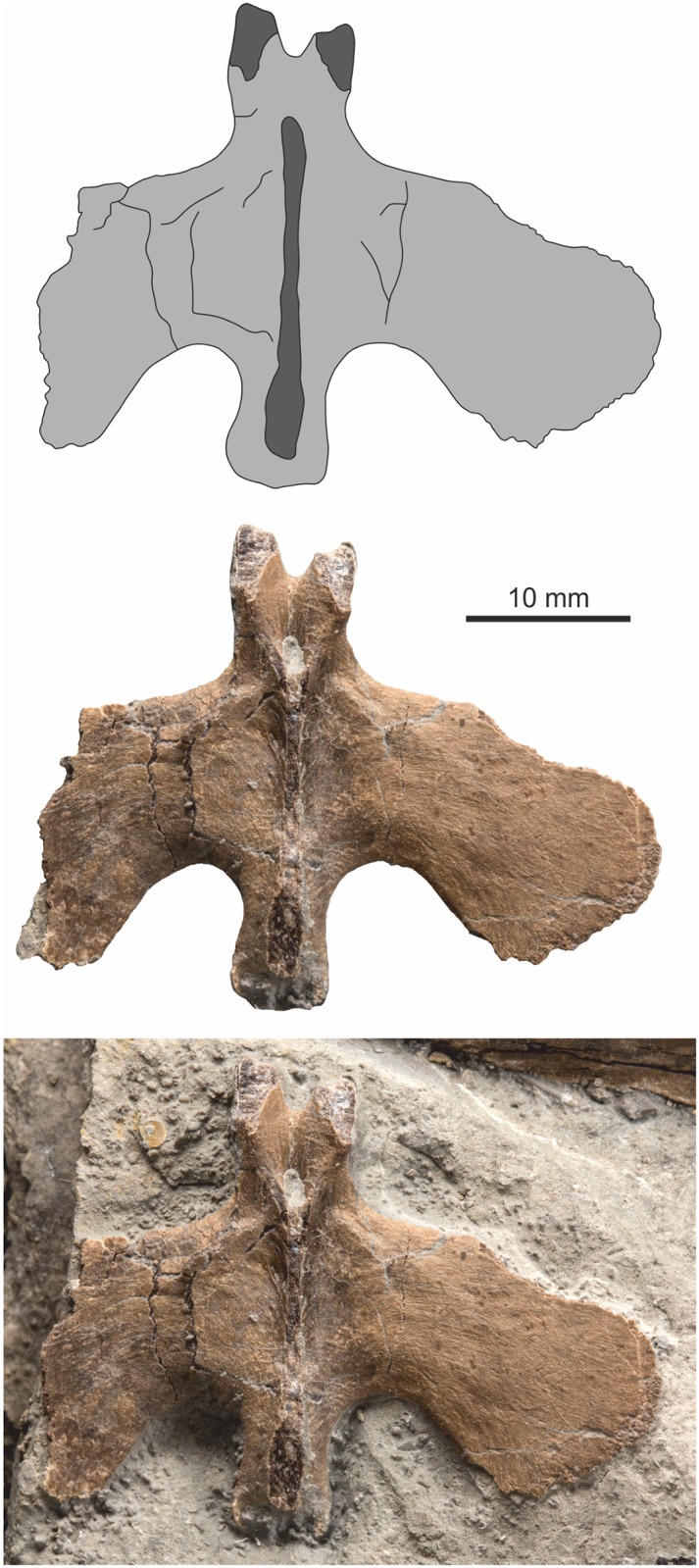
Neural arch of caudal vertebra seen in dorsal aspect. The neural spine is broken, but note how thin it is at its base.

At least five caudal vertebrae are in loose articulation and closely associated with the preserved ischium. No fused sacral vertebrae are present, but an anterior caudal vertebra with robust, broad lateral processes appears to be a partially sacralised element (Fig 16) and is considered here to represent the first caudal vertebra. Its centrum is similar in length to the more caudal three centra with which it is loosely articulated, and it lacks facets for chevrons. The second and third caudal vertebrae are damaged, with only the ventral face of the centra seen. The fourth caudal vertebral centrum is crushed, but there is a chevron element in articulation between the third and fourth caudal centra. The ramus of the chevron appears to be curved caudo-ventrally, but this is exaggerated as an artefact of crushing. A chevron associated with the caudal margin of the fifth caudal vertebra is only gently curved. Each caudal centrum has a pair of near parallel carinae that are more prominent caudally, fading cranially such that the ventral surface of the centrum is smoothly rounded cranially. At least three disarticulated neural arches are associated with the caudal centra, bearing flat, broad lateral processes. A similar neural arch (Fig 17) is seen in dorsal view showing equal sized anterior and posterior zygapophyses. An isolated caudal vertebral centrum lacking neural arch from a more distal part of the tail is also present.

#### Ventral rib cage

Several (n = ~8) isolated, thin rib-like elements with tapered termini are interpreted as gastralia (Fig 15c). They are gently curved with a circumferential length of between 50 mm and 60 mm and a maximum thickness in their central region of 4 mm. An elongate facet is present on either the cranial or caudal surface, presumably for accommodating an adjacent lateral gastral rib.

#### Furcula

A slender, curved, symmetrical element in slab NMW 2015.5G.3 is probably the furcula. It is 80 mm long, but missing two or three millimetres from one end preserved as an external mould. There is a fine carina extending along its caudal and cranial margins for much of its length, but not reaching either of the distal termini where the furcula articulates with the scapulocoracoids. The two rami diverge at an angle of approximately 125°, with no discernible suture between the two rami. The element differs from a number of medial gastralia present on the slab in its possession of the distinctive carinae. Like that of the coelophysoid *Segisaurus halli*[13], the furcula is a thin, slender element lacking a hypocleideum, although possibly the carina represents this structure. The furcula of *Segisaurus* does not appear to have the carinae[13], but that of *Syntarsus rhodesiensis*[9] has a short apical ridge in the same region as the carina in *Dracoraptor*. Furculae have not been reported for any other non-coelophysoid Late Triassic or Lower Jurassic Theropoda[62].

### Forelimb

A partial left forelimb is preserved comprising a distal humerus, complete radius and ulna, metacarpals, phalanges and unguals. They are loosely associated with the radius and ulna in approximate articulation and only slightly displaced from the humerus. There are no carpals, presumably a consequence of the immature age of the individual and consequent delayed ossification of the carpal elements[61], but it cannot be ruled out that they have simply been lost. Because of the degree of disarticulation of the manual elements the phalangeal formula has not been determined.

#### Humerus

**Fig 18:**
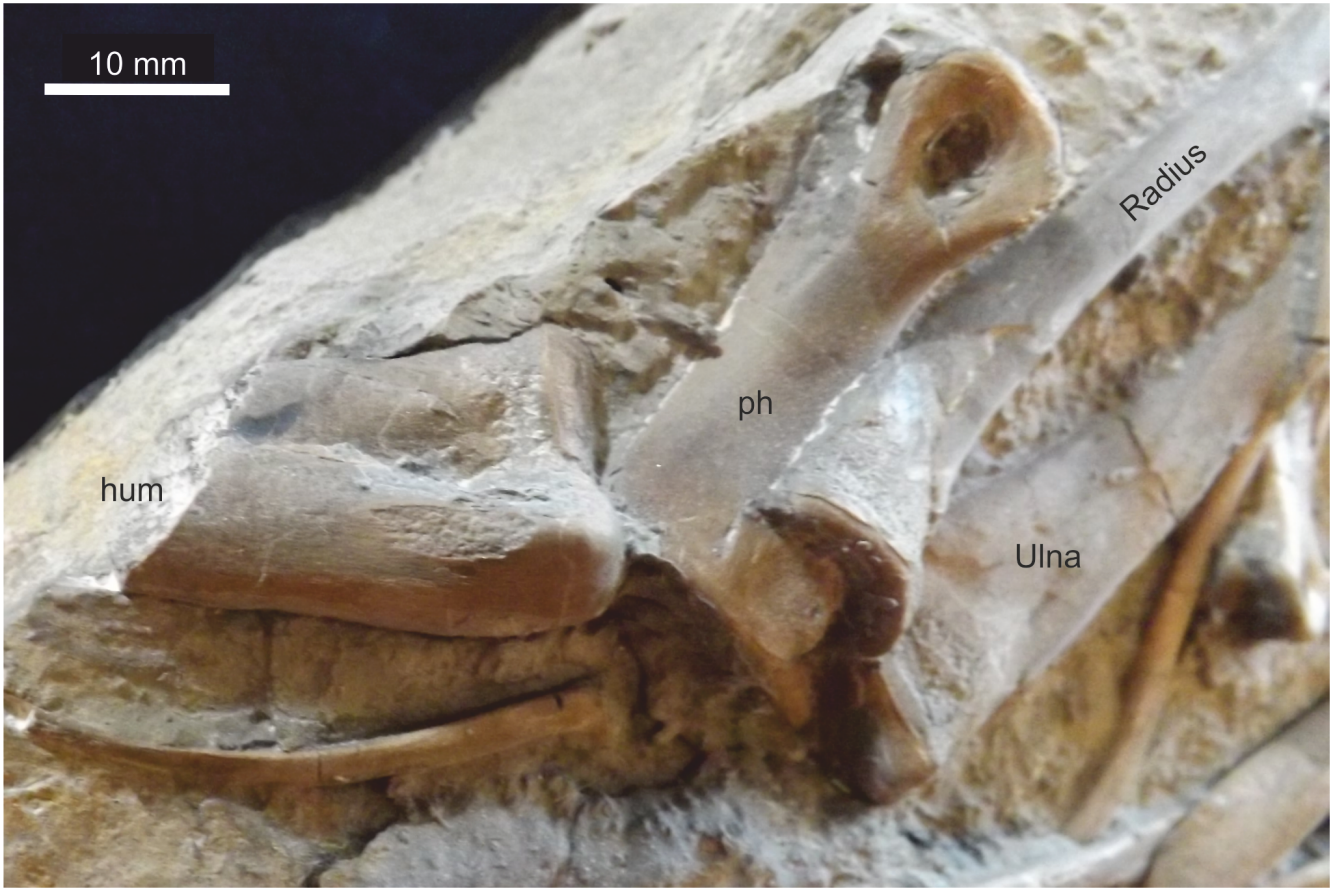
Distal left humerus. Note the close proximity to the articulated radius and ulna.

Only the distal articulation of the left humerus is preserved with the proximal? ^3^/_4_ missing, but a clean break at the edge of the slab suggests it was entire (Fig 18). It shows the articulatory surface for the radius and ulna exposed in caudal view. A suture between the articulatory epiphyseal surfaces is well defined and an elongate depression between the two distinct articulating surfaces extends proximally for all the length of the preserved segment. The diameter at its broken end is ~12 mm and ~17 mm at its distal termination.

#### Ulna

**Fig 19:**
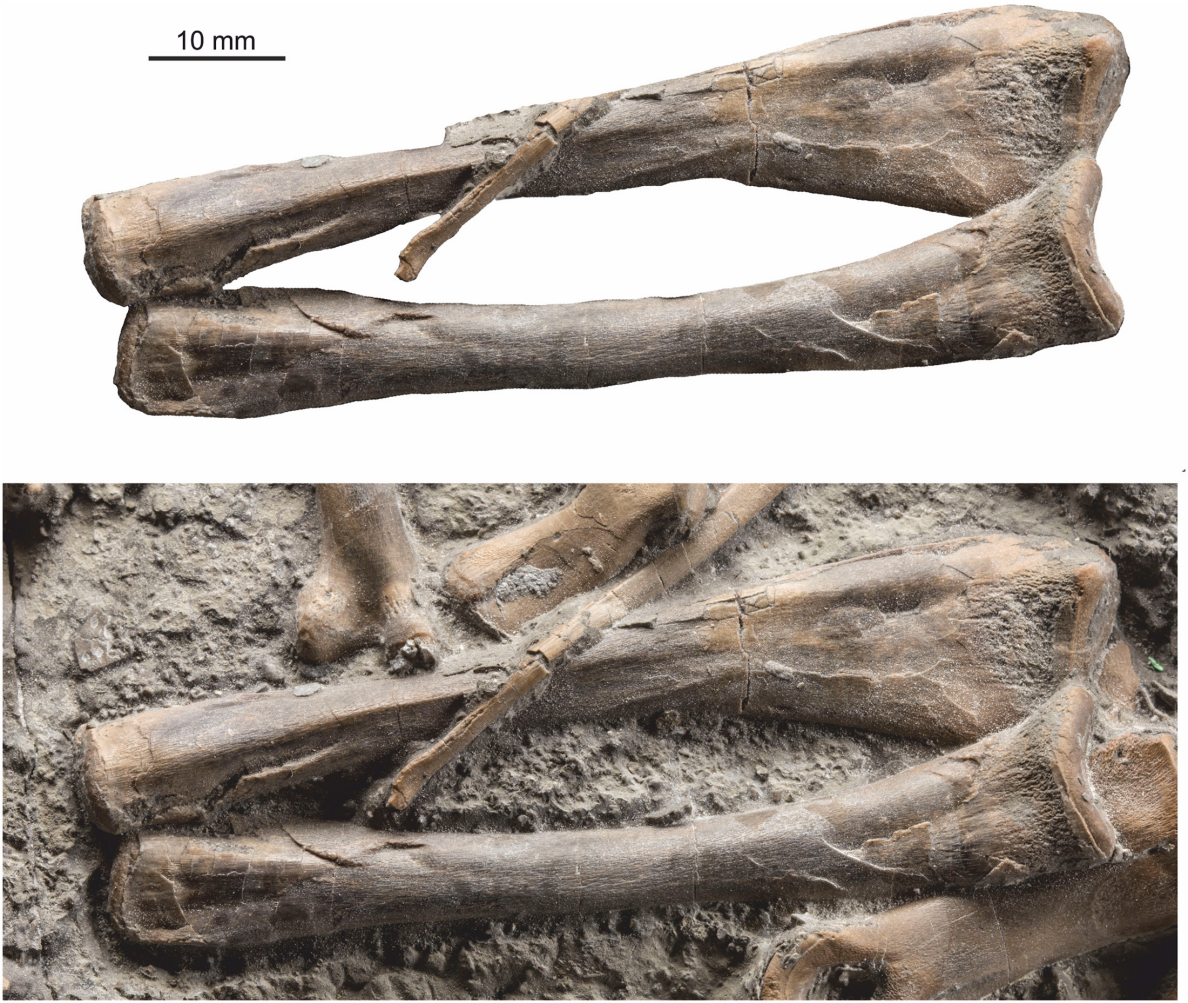
Radius and ulna. Top, matrix digitally removed for clarity. Below, bones in context. The ulna is considerably broader proximally than the radius.

The left ulna is well preserved and entire (Fig 19). It is slender, expanded both proximally and distally, with the proximal expansion being greater than the distal expansion, and also greater than the proximal expansion of the adjacent radius. The proximal condyle surface is smooth and well defined, marked from the diaphysis by a prominent suture. The proximal ulnar crest is distinct for about a quarter of the ulna length, while a distal carina is present on the same (medial) surface from the middle of the diaphysis to almost the distal end. The proximal end is almost twice as wide as the distal end. The total length is 73 mm, and at the narrowest part of the diaphysis it is ~ 7 mm diameter.

#### Radius

The left radius, associated with the ulna is entire (Fig 19). It is 71 mm long, slender with only slight expansion at the proximal and distal ends. At its narrowest part of the diaphysis it is ~ 6 mm diameter.

#### Metacarpals

**Fig 20:**
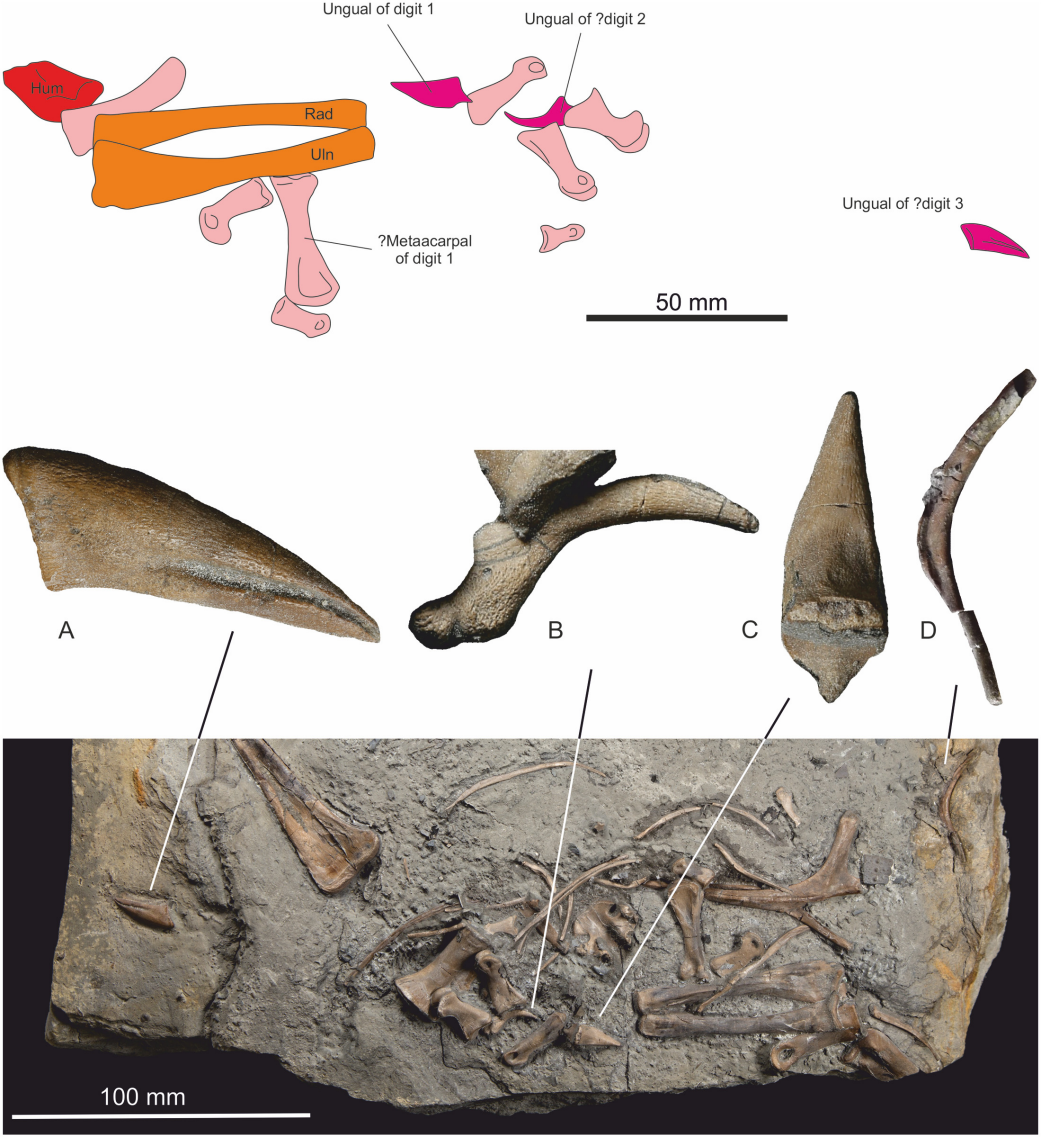
Manual elements. Outline diagram showing the disarticulated assemblage of manual elements relative to the articulated radius and ulna. 20 (A-C) Morphology of unguals. Exact position in manus uncertain; (D) furcula.

A single possible metacarpal of digit 1 is preserved (Fig 20). It is characterised by an obliquely arranged distal articulatory surface that probably allowed the first digit to oppose the other digits. The element compares closely with that of *Dilophosaurus*, but has a somewhat longer aspect ratio.

#### Phalanges

Several phalanges are preserved and are complete, but all are disarticulated (Fig 20). They are assumed to be from the left manus as they are associated with the left radius and ulna. A tentative restoration suggests that the manus was approximately the same length as the forearm (see Fig 5).

#### Unguals

Several ungual elements can be identified, but they are disarticulated and randomly orientated. One example (Fig 20) has a prominent proximally directed process on its dorso-proximal margin and compares well with the ungual of digit one of *Dilophosaurus wetherilli* Welles, 1954[12]. Another ungual has no discernible process, while a third ungual is obscured by an overlying phalange.

### Pelvic girdle and hind limb

Elements of the pelvis and hind limb are preserved, but none are complete or in articulation. Both ilia are missing and only the left and right pubes and? left ischium are preserved. The right femur, partial fibula and portion of probable tibial diaphysis are present, while one disconnected slab has three proximal portions of the metatarsals, partially preserved as external moulds (see below).

#### Pubis

**Fig 21:**
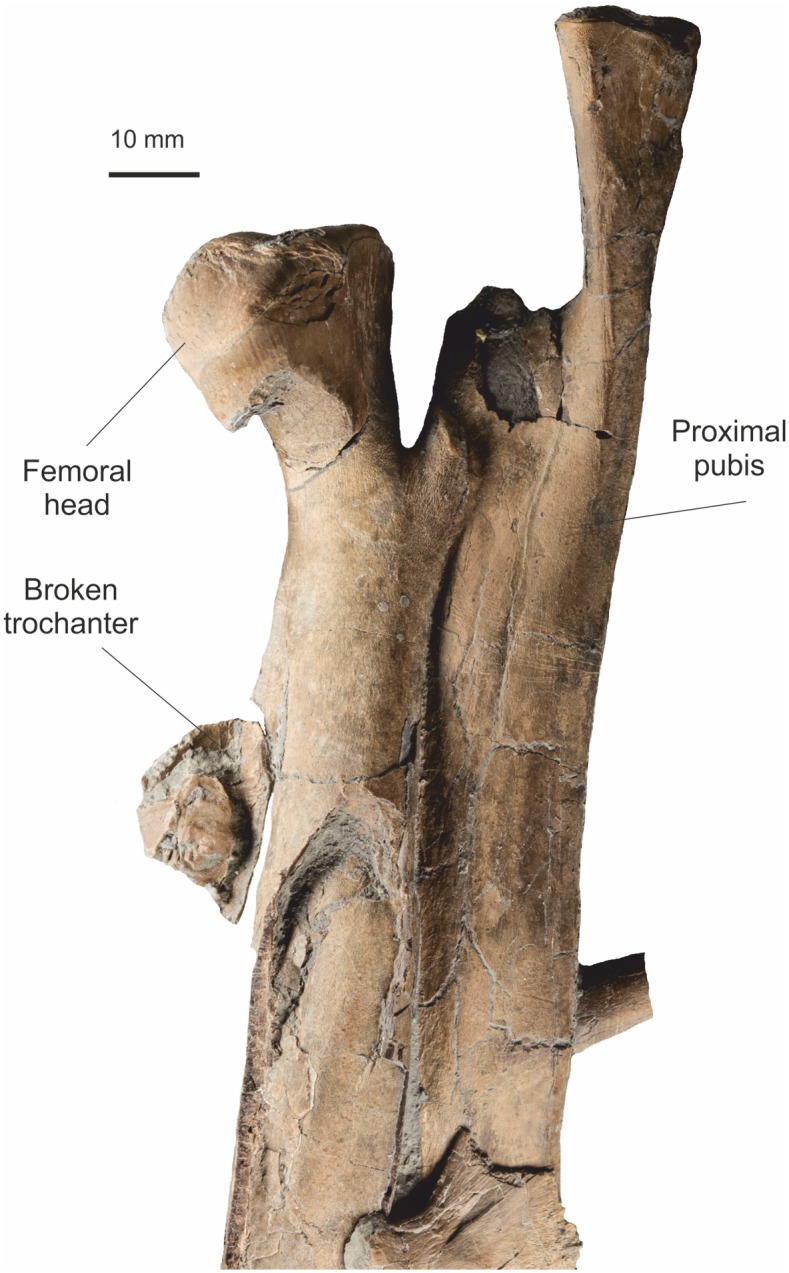
Proximal left femur and pubis. The matrix has been digitally removed for clarity.

**Fig 22:**
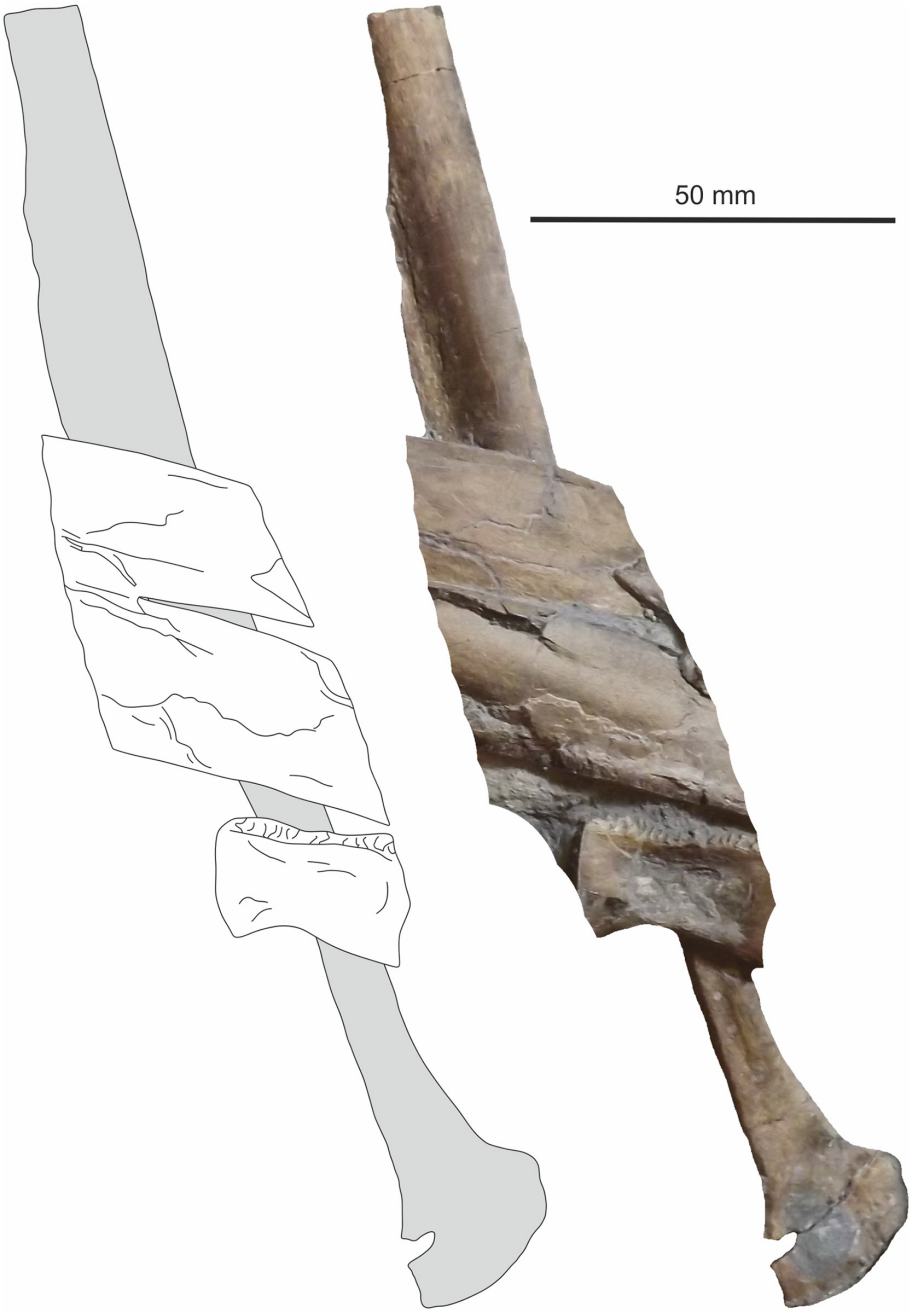
Pubis. Distal left(?) pubis (matrix digitally removed for clarity).

A near complete right pubis, missing the distal ¼, and the distal ⅔ of the left pubis are preserved (Figs 21 and 22). They are not united along the medial symphysial lamina, but lie across each other on the slab. Both were complete with cleanly broken termini. An approximate total length of 212 mm is determined by overlapping the two elements making the pubis approximately 80 mm longer than the ischium. The right pubis is displayed in cranio-lateral view, while the left is seen in medial view. There is only a very gentle eminence for the insertion of M. puboischiofemoralis. Distally the pubic boot is gently expanded both cranially and caudally in almost equal amounts, but its total expansion is only about twice the width of the pubic shaft in front of the expansion.

#### Ischium

**Fig 23:**
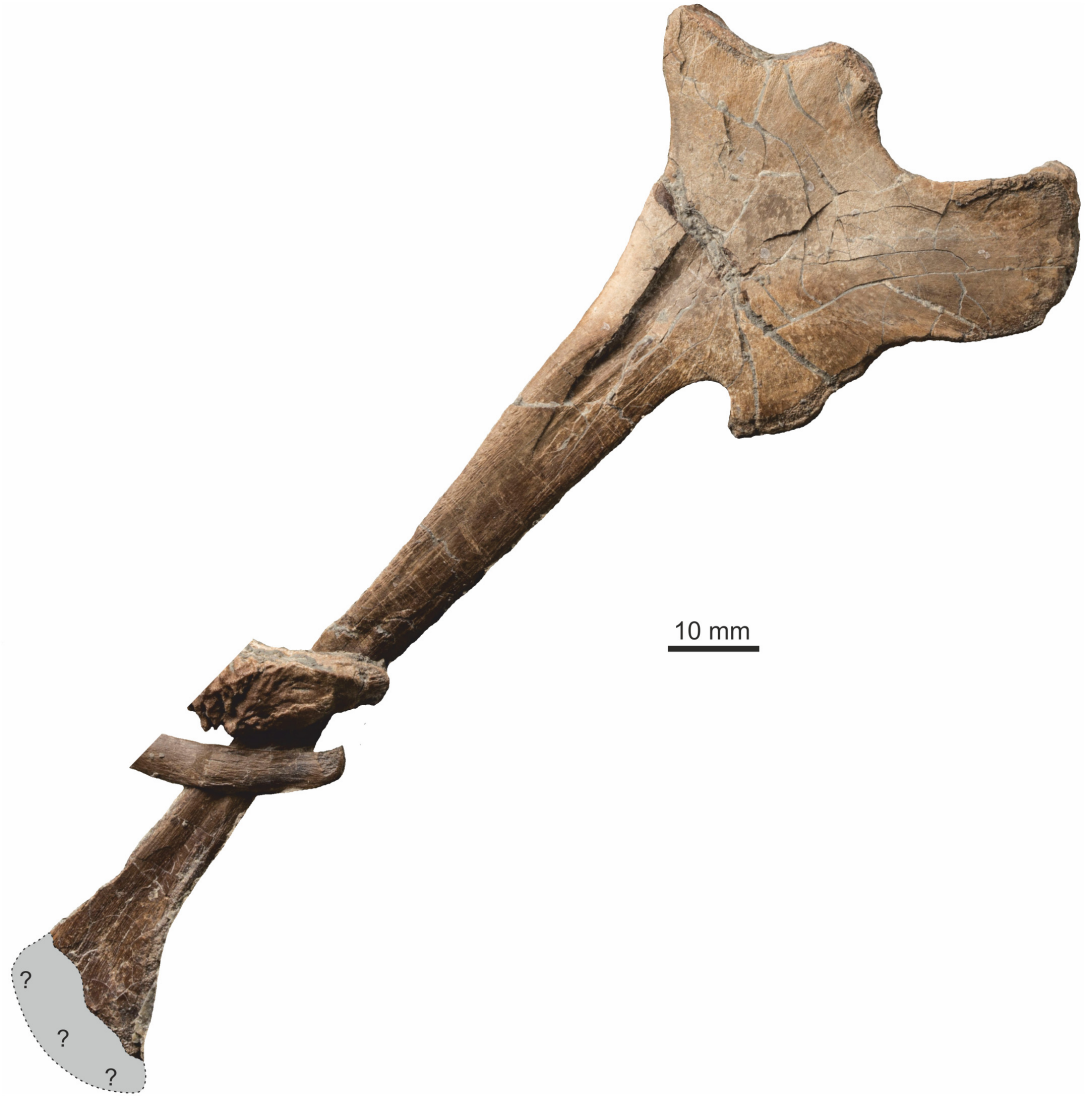
Left ischium. Almost complete element with only the distal tip missing, here minimally restored. The overlying elements are a chevron and caudal neural arch (matrix digitally removed for clarity).

A single near complete ischium from the left side seen in medial view is preserved (Fig 23), missing only the very distal few millimetres of the ischial boot. Proximally the acetabulum comprises the anterior half of the proximal margin and is deeply concave. The surface for articulation with the ischial process of the pubis is straight and forms an angle of approximately 45° to the shaft of the ischium. The articulatory surface of the ilial process is gently concave and forms approximately half of the proximal border of the ischium. There is a prominent obturator process on the anteroventral surface. The ischial boot is slightly more expanded cranioventrally than caudodorsally.

#### Femur

The femur is complete for its proximal ⅔, missing the distal condyles (Fig 21). It is exposed in antero-medial view and displays the femoral head prominently. It is overlain by a rib, but this does not obscure detail. The femoral head (greater trochanter) is ‘hooked’ with a prominent smooth articular condyle. There is a prominent lesser trochanter that is approximately ^2^/_3_ the height of the greater trochanter and forms a distinctive V shaped groove in medial view. The fourth trochanter is prominent, but damaged by compaction. Part of the distal femur is lost.

#### Tibia

**Fig 24:**
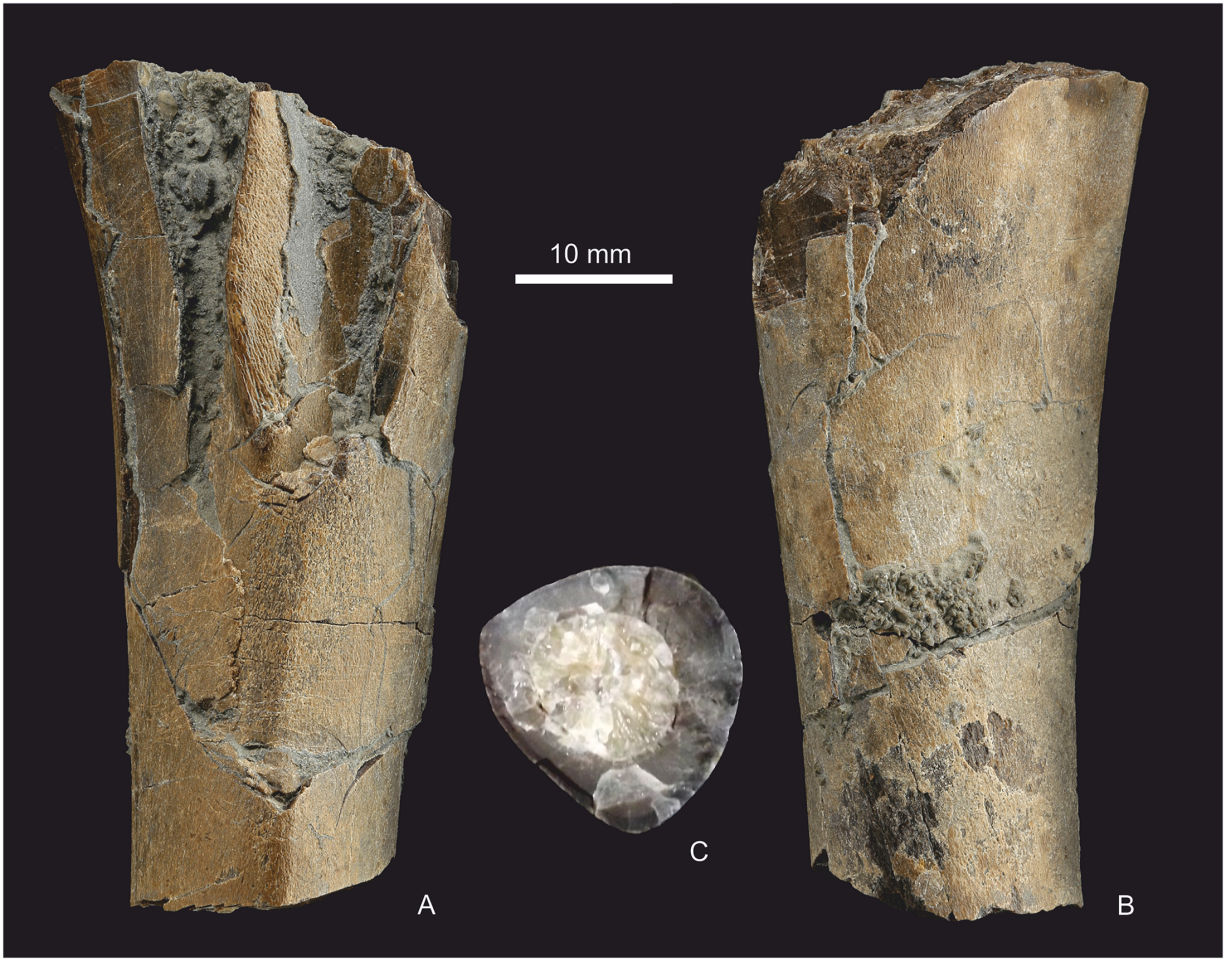
Partial left tibia from proximal third. (A) Fragment in lateral view, (B) in medial view and (C) the distal cross section.

A small portion (max length ~ 40 mm) of the diaphysis of a probable tibia is present (Fig 24). It is not attached to a slab, but clean breaks indicate it was more complete, and the remainder is probably lost. A lumen is filled with white calcite. The cross-sectional shape is reminiscent of that of theropod tibiae, but there are no distinctive landmarks to confirm its identification, although a slight flaring suggests the preserved portion is from the proximal end.

#### Fibula

**Fig 25:**
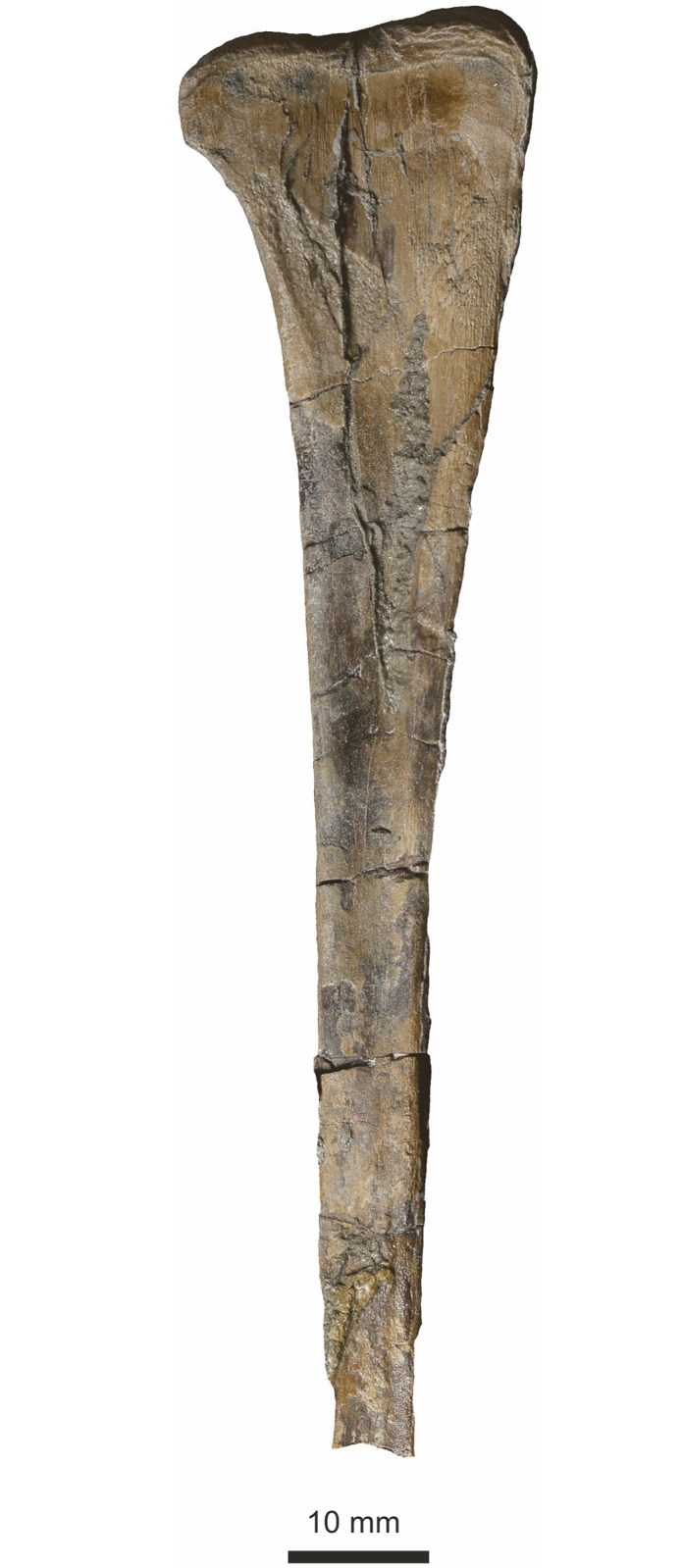
Proximal part of fibula in medial view. Probably the left.

The proximal portion of a left fibula is present (Fig 25). It is flat on its medial surface where it was adpressed against the tibia, but this flatness may be exaggerated by post burial compaction. It has a broad, straight proximal epiphysis and tapers evenly on its cranial margin, and more rapidly with a distinct curve on its caudal margin giving the bone an asymmetry. There is a very slight eminence on the caudal margin approximately 10 mm from the proximal end that may represent a muscle scar. Maximum length preserved ~100 mm. Maximum width of proximal articulatory surface 26 mm, tapering to 6 mm at its distal end and resembles closely the fibula of *Dilophosaurus*.

#### Metatarsals

**Fig 26:**
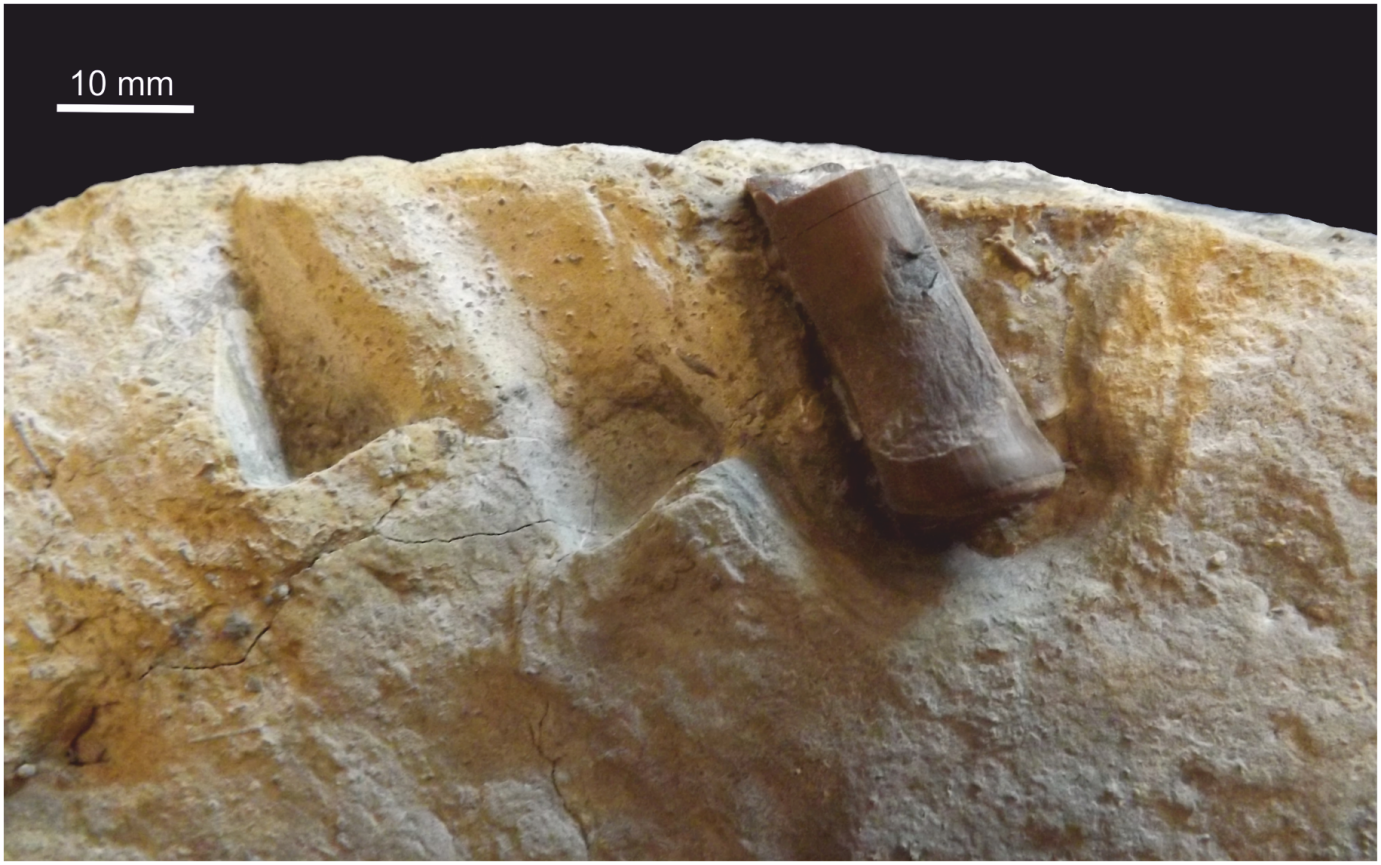
Proximal portions of right metatarsals. Only part of metatarsal? IV is preserved, with external moulds of II and III in approximate articulation.

Three right proximal metatarsals are present on one slab, two preserved as external moulds, with the distal parts missing and a third well preserved, but also missing its distal end. Thus no reliable length measurement can be obtained. They appear to be in close enough association and with similar orientation to suppose they are close to true articulation, although there is an equidistant offset between each metatarsal. The distal articulatory surfaces for the tarsals are flat. The diaphysis are 8 mm diameter with a flattened oval ‘D-shaped’ cross-section (Fig 26).

**Fig 27:**
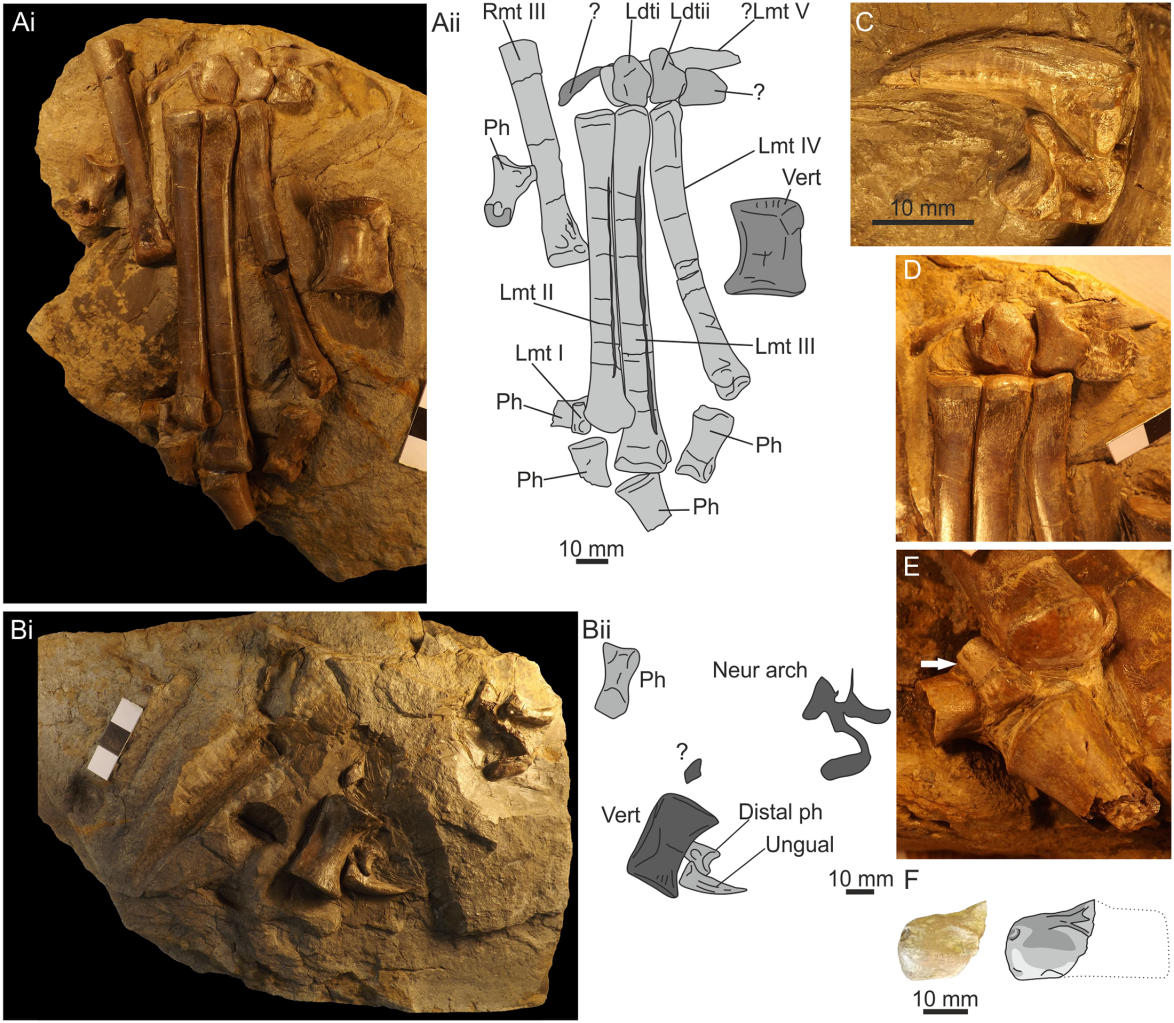
Left pes and part of right pes. (Ai) Photo of main slab with elements of left pes in approximate articulation. (Aii) Outline drawing of the same with elements identified. (Bi) Counter slab with some elements of right pes, including two ungual elements. (Bii) Outline drawing of the same with elements identified. (C) Ungual and associated distal phalanx. (D) Distal tarsals and proximal portion of metatarsals II-IV. (E) Distal portion of metatarsal I preserved in articulation next to metatarsal II. (F) Partial astragalocalcaneum in cranial view, photo on the left, interpretive drawing on the right. Abbreviations: L, left; R, right; dt, distal tarsal; mt, metatarsal; Ph and ph, phalanx; Vert, vertebra.

Additional elements of both pedes were discovered in a second cliff fall on 20th July 2015 by University of Portsmouth student Sam Davies (Fig 27). The remains occur on part and counterpart slabs each measuring approximately 270 x 170 mm. These are accessioned into the National Museum of Wales as NMW 2015.10G.1 a+b. The slab has split between the skeletal elements leaving the majority of the left tarsus, metatarsus and a vertebral centrum on one slab (NMW 2015.10G.1 a [Fig 27Ai & 27ii]), with a few elements of the right metatarsus, a short phalange, an ungual, a vertebral centrum and a separate neural arch on the counterslab (Fig 27Bi & 27ii). There are also two isolated metatarsal fragments that were picked up separately. It has not proved possible to unite the distal elements of the right metatarsus with the proximal elements preserved on slab NMW 2015.5G.4.

The left tarsus comprises a fragment of astragalus which is broken in its midline revealing a slightly dished ventral surface (Fig 27F) and a slight dorsal process, although some of this may be missing. A calcaneum is not present. Two distal tarsals (dt III & dt IV) and part of a putative third are present in a row. These lie adjacent to the third and fourth metatarsals and a broken element lies where the fifth metatarsal would articulate with the ankle.

The first metatarsal is represented only by the distal end and is considerably thinner than metatarsals II to IV with a diaphyseal diameter of just 4 mm before a slight expansion at the distal condyle (Fig 27E). Metatarsal II is elongate (102 mm), straight with a flattened face on the proximolateral margin for tight adpression with metatarsal III. The distal condyle of mt II has a prominent process on its lateral distoventeral surface. Metatarsal III is the longest of the series (116 mm). The medioproximal and lateroproximal faces are flattened for adpression with adjacent metatarsals. Metatarsal IV is shorter than both Mt II and Mt III (93 mm) and is gently curved laterally. The distal condyle is complex with a deep mediolateral notch dividing it into two distinct regions. Phalanges are in loose articulation with Mt II, Mt III and Mt IV, but a phalangeal formula cannot be determined.

### Phylogenetic Analysis

**Fig 28:**
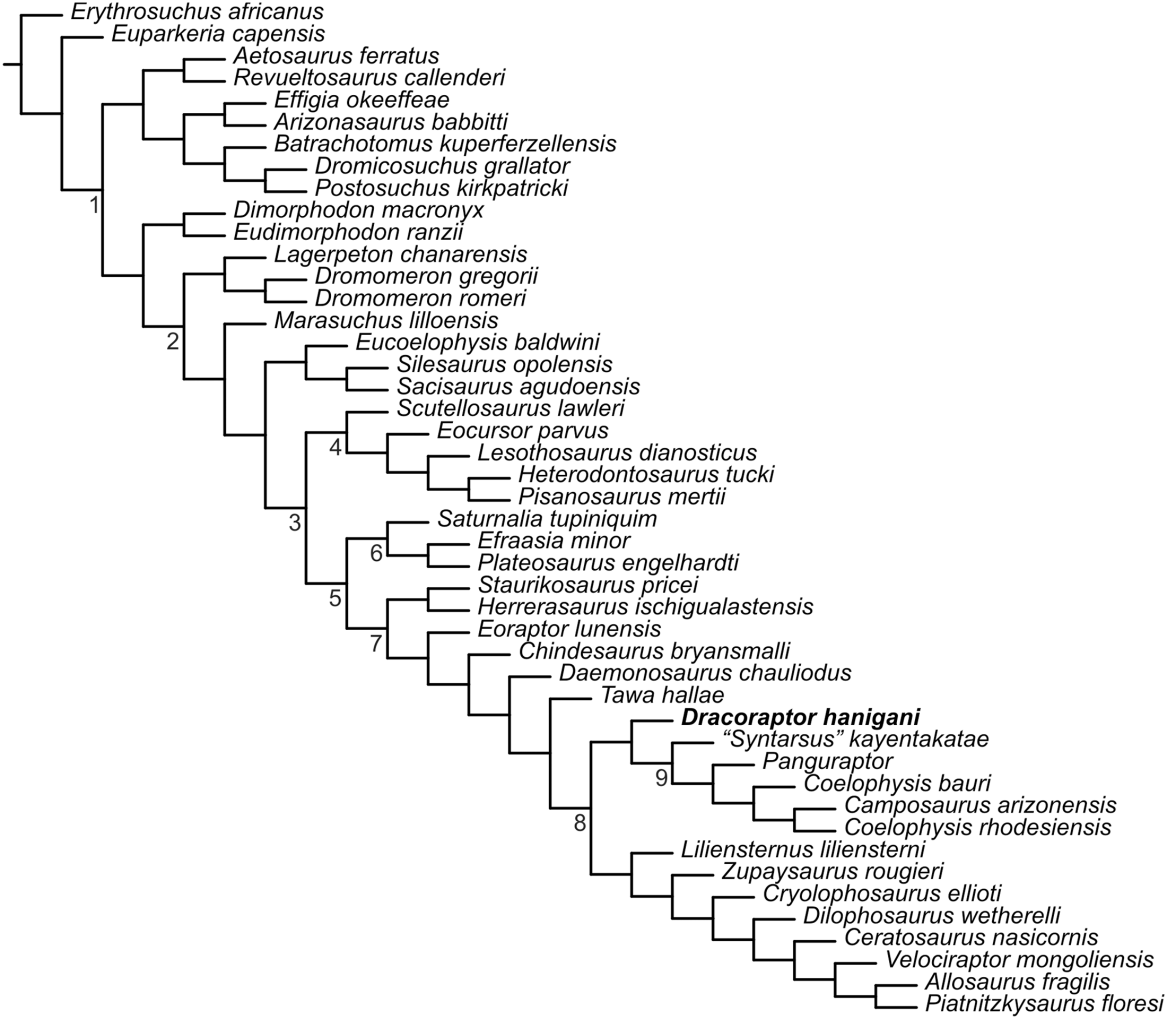
Cladisitic analysis of *Dracoraptor hanigani*. 1. Archosauria; 2. Dinosauromorpha; 3. Dinosauria; 4. Ornithischia; 5. Saurischia; 6. Sauropodomorpha; 7. Theropoda; 8. Neotheropoda; 9. Coelophysidae.

The analysis of 366 characters for 46 taxa resolved Neotheropoda, with *Tawa hallae* as its sister taxon, similar to the analysis of Nesbitt et al.[61], Ezcurra and Brusatte [25] and You et al.[24] (Fig 28). However, the topology of the basal region of Neotheropoda differs, being more similar to the results of the Sues et al.[26] analysis. The “coelophysoids” *Liliensternus liliensterni* and *Zupaysaurus rougieri* are now successive sister taxa to crown group neotheropods, making the contents of Coelophysoidea *sensu* Nesbitt et al.[61] synonymous with Neotheropoda. *Dracoraptor hanigani* is recovered as the basal most coelophysoid, sister taxon to “*Syntarsus*” *kayentakatae* and all other coelophysids. Coelophysidae is supported by 3 synapomorphies (15 in the bootstrap) and Neotheropoda is supported by 23 synapomorphies. The bootstrap results demonstrate that although Neotheropoda is well supported, the basal part of the group is not. *Dracoraptor hanigani*, *Liliensternus liliensterni*, *Zupaysaurus rougieri*, *Dilophosaurus wetherelli*, *Ceratosaurus nasicornis* and *Cryolophosaurus ellioti* are found in a polytomy with the remaining clades of Neotheropoda; Coelophysidae and an unnamed clade containing *Velociraptor*, *Allosaurus* and *Piatnitzkysaurus*.

*Dracoraptor hanigani* possesses 18 apomorphies, some of which are convergent with more derived neotheropods, but it also retains plesiomorphic conditions, such as three teeth in the premaxilla.

## Discussion

*Dracoraptor hanigani* is clearly a saurischian dinosaur on account of its pelvic construction, while the possession of serrated, recurved laterally compressed teeth among other characters demonstrates its theropod affinities. The cladistic analysis finds *Dracoraptor* to lie within Neotheropoda, but is basal within the clade. *Dracoraptor* can be distinguished from other basal neotheropods from the Late Triassic and Early Jurassic on a variety of criteria, not necessarily apomorphic. However, *Dracoraptor* possesses a combination of basal characters that make it difficult to place phylogenetically. The shallow antorbital fossa of the maxilla, an anteriorly located pleurocoel on the cervical vertebrae and the presence of an obturator notch in the ischium indicates neotheropodan affinities ([63], characters 12, 88, 190 respectively), but many of the other neotheropodan synapomorphies cannot be identified in *Dracoraptor*. The cladistic analysis recovers *Dracoraptor* as the sister taxon to “*Syntarsus*” *kayentakatae* and all other coelophysids placing it close to the base of Coelophysoidea (Fig 28). However, several of the synapomorphies of Coelophysoidea are coded ‘?’ for *Dracoraptor*. Notably the horizontal ridge of the maxilla parallel to the tooth row (character 15 of[63]) cannot be seen as both left and right maxillae expose their internal surface. The sublacrimal process of the jugal of *Dracoraptor* is certainly not pointed as it is in some non-coelophysoid theropods, but this process is slender, slightly damaged and it is ambiguous as to whether it is bluntly rounded (Character 23, state 1 Rauhut 2003)[63] which is typical for coelophysoids. *Dracoraptor* retains many basal features, and would appear to be very basal within Neotheropoda. It is likely that further preparation of the specimen will enable the identification of additional characters that may better resolve its phylogenetic affinities.

**Fig 29:**
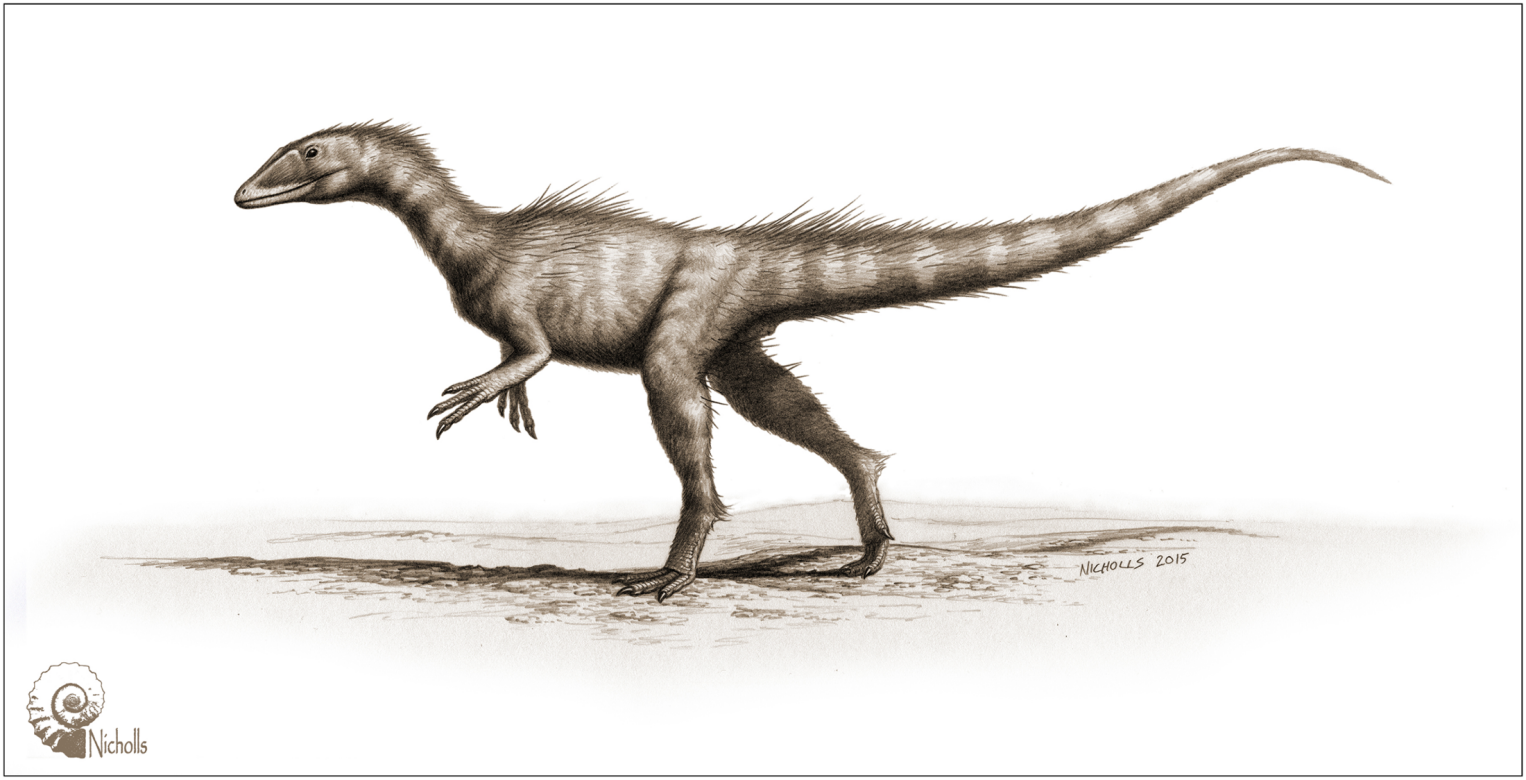
*Dracoraptor hanigani* restored as a shoreline dwelling predator and scavenger. Artwork by Bob Nichols (paleocreations.com).

The presence of *Dracoraptor* in marine strata may be of little significance with regard to the animal’s autecology, but we have restored it as a shore-line dwelling animal (Fig 29).

## Supporting Information

S1 File Cladistic procedure. The file contains cladistic characters, the compound characters that were atomized, experiments demonstrating the utility of atomized compound characters, a data matrix, and results of a cladistic analysis with bootstrap analysis.
(DOCX)
