# Correction: The Oldest Jurassic Dinosaur: A Basal Neotheropod from the Hettangian of Great Britain

**DOI:** 10.1371/journal.pone.0154352

**Published:** 2016-04-20

**Authors:** David M. Martill, Steven U. Vidovic, Cindy Howells, John R. Nudds

There is an error in the final sentence of the first paragraph of the Introduction. The correct sentence is: Two theropods have been named from the English Hettangian, Sarcosaurus woodi Andrews, 1921[4] from Barrow upon Soar, Leicestershire, based on an isolated pelvis, vertebra and proximal femur (NHMUK 4840/1) and Sarcosaurus andrewsi Huene, 1932 [16] based on a partial tibia (NHMUK R3542) (see also ref [3]).

There is an error in the final sentence of the second paragraph of the subsection “Dinosaurs in Wales” within the Introduction. The correct sentence is: The specimen is deposited in Amgueddfa Cymru—National Museum Wales, Cardiff, accession number NMW 2015.5G.1–2015.5G.11 and NMW 2015.10G.1 with individual blocks and skeletal elements numbered as in Table 1 (Fig 5).

There is a sentence missing before the final sentence of the first paragraph of the subsection “Basic collecting, preparation and repair” within the Materials and Methods. The last three sentences of this paragraph are: Extensive searching failed to discover the missing bones on the foreshore. In July 2015 a block containing the left pes was recovered by Mr. Sam Davies. There exists the possibility that some of the specimen remains in the cliff, but repeated careful examination of the cliff face failed to reveal any exposed bones. There are errors in Fig 4, “Stratigraphic log for Lavernock Point.” Please see the correct Fig 4 here.

**Fig 4:**
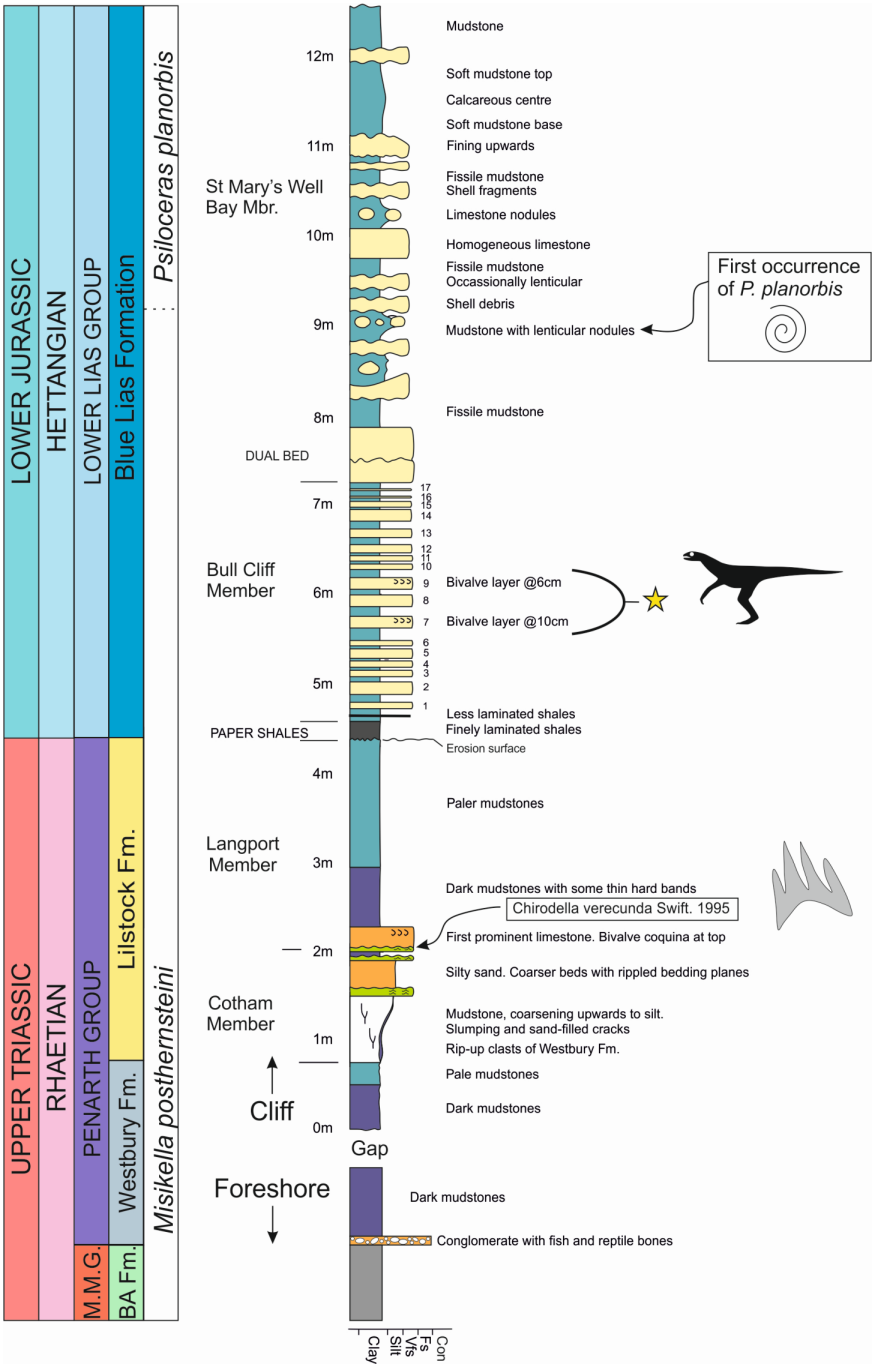
Stratigraphic log for Lavernock Point. The lithological boundary of the base of the Jurassic is the base of the Paper Shales horizon at 4.5 metres. Many British stratigraphers have historically used the first occurrence of the ammonite *Psiloceras planorbis* to indicate the base of the Jurassic, but elsewhere in Europe two other psiloceratacean ammonites appear before *P*. *planorbis*. The thin limestone at the base of the Langport Member, at 2.2 m, has been dated as Rhaetian by Swift (1995) using conodonts. The new theropod comes from one of the limestones at the 6 m interval (bed 7 or 9).

There are errors in the first sentence of the Holotype subsection of the Systematic Palaeontology subsection of the Results. Please see the correct sentence here: NMW 2015.5G.1—2015.5G.11 and NMW 2015.10G.1 are a disarticulated, but associated partial skeleton with elements of the skull, including both premaxillae, both maxillae, some teeth, a lacrimal, partial jugal, post orbital, squamosal, fragmentary lower jaws and a possible hyoid, and postcranial skeleton including two cervical vertebrae, posterior elements of the vertebral column (lumbar and caudal vertebrae), distal forelimb, ischium and pubis, hind limb with femur, and fragmentary tibia with proximal fibula and left pes.
