# Supplementary material for: The Oldest Jurassic Dinosaur: A Basal Neotheropod from the Hettangian of Great Britain
Source: PLoS One. 2016 Jan 20;11(1):e0145713. doi: 10.1371/journal.pone.0145713 (PMC4720452; doi:10.1371/journal.pone.0145713)
Supplement: S1 File — The file contains cladistic characters, the compound characters that were atomized, experiments demonstrating the utility of atomized compound characters, a data matrix, and results of a cladistic analysis with bootstrap analysis. (DOCX) [file pone.0145713.s001.docx]

**S1: Supporting Information**

The oldest Jurassic dinosaur: a basal neotheropod from the Hettangian of Great Britain

*by* DAVID M. MARTILL, STEVEN U. VIDOVIC, CINDY HOWLLS, JOHN NUDDS

David M. Martill [*david.Martill@port.ac.uk*]; Steven U. Vidovic [*steven.vidovic@port.ac.uk*], Palaeobiology Research Group, School of Earth and Environmental Sciences, University of Portsmouth, Burnaby Building, Burnaby Road, Portsmouth, PO1 3QL, United Kingdom. Cindy Howells [cindy.howells@museumwales.ac.uk], Department of Natural Sciences, Amgueddfa Cymru - National Museum Wales, Cardiff, United Kingdom. John R Nudds [*john.nudds@manchester.ac.uk*], School of Earth, Atmospheric and Environmental Sciences, University of Manchester, Manchester, United Kingdom.

**Table of contents**

**S1.1.1.** Character generation and coding methods

**S1.1.2.** Treatment of compound characters

**S1.1.3.** Character list

**S1.2.1.** Data matrix

**S1.3.1.** Jacknife - comparison of datasets

**S1.4.1.** TNT procedure

**S1.5.1.** TNT results

**S1.5.2.** Cladogram

**S1.5.3.** Synapomorphies/apomorphies of key nodes

**S1.5.4.** Bootstrap analysis

**S1.1.1.**

The 366 characters in the analysis presented here are a product of the 339 characters of Ezcurra and Brusatte (2011) (after Nesbitt et al. 2009). The additional 27 characters were produced by atomizing or modifying the compound characters (in Ezcurra and Brusatte 2011) into their component characters (Brazeau, 2011) (see S1.1.2.). The taxon list and coding of You et al. (2014) was used and re-coded accordingly. Dashes replaced question marks for characters 330, 331, 333 and 334 when character 329 was coded with a 0. The reason for this recoding is the four characters coded with a dash concern osteoderms on the dorsal surface of the organism and character 329 codes these osteoderms as present or absent.

The taxon *Daemonosaurus* is added to the analysis due to it sharing a plesiomorphic condition regarding the number of teeth in the premaxilla with *Tawa* and *Dracoraptor*. The analysis Sues et al. (2011) used to accompany the description of *Daemonosaurus* is also an adaptation of the Nesbitt et al. (2009) analysis. Therefore, it was possible to use the coding of Sues et al. (2011) for characters 1-315. The additional 24 characters were coded using the figures and text (Sues et al. 2011). The compound characters were subsequently split into their constituent parts as with all other taxa.

Taxa with multiple states for the same character are coded with an ‘&’ between the states. When there is uncertainty as to which state is applicable, but there is evidence for both ‘/’ is used between the states. Taxa with unknown states for a character are coded with ‘?’, those with non-applicable states are coded with ‘-’, all other character states are coded between 0 and 4.

Ezcurra and Brusatte (2011) used ordered characters, but You et al. (2014) did not, despite not noting an adjustment to the cladistic procedures in their paper. In this analysis ordered characters are not used, because it assumes a complete fossil record and a direction to evolution.

**S1.1.2.**

The compound character, 30 of Ezcurra and Brusatte (2011) has been rewritten and is character 31 of this analysis. It was possible to atomize the following compound characters into two or more characters; 14 (14 & 15), 57 (58 & 59), 67 (69 & 70), 77 (80 & 81), 106 (110 & 111), 128 (133 & 134), 131 (137 & 138), 137 (144 & 145), 151 (159 & 160), 191 (200 & 201), 196 (206 & 207), 211 (222 & 223), 223 (235, 236 & 237), 224 (338 & 339), 230 (245 & 246), 231 (247 & 248), 236 (253 & 254), 237 (255 & 256), 254 (273 & 274), 273 (293 & 294), 315 (336 & 337), 317 (339 & 340), 319 (342, 343 & 344), 320 (345 & 346), 323 (349 & 350). The new character numbers are in brackets above. The characters of Ezcurra and Brusatte (2011) that have not been atomized have been reordered as follows in character list below.

**S1.1.3.**

1) Premaxilla, height: length ratio below external naris: .5-1.25 (0); <.5 (1); >1.25 (2). Smith et al. 2007: 5; Irmis 2008: 9

2) Premaxilla, anterodorsal process (=nasal process), length: less than the anteroposterior length of the premaxilla (0); greater than the anteroposterior length of the premaxilla (1). Nesbitt and Norell 2006; Nesbitt 2009: 1

3) Premaxilla, angle of the anterodorsal process (=nasal process) relative to the alveolar margin: more than 75 degrees (0); less than 70 degrees (1). Smith et al. 2007: 6; Irmis 2008: 10

4) Premaxilla, posterodorsal process (=maxillary process, = subnarial process), length: less than or about the same as the anteroposterior length of the premaxilla (0); greater than the anteroposterior length of the premaxilla (1). Nesbitt 2009: 2

5) Premaxilla, posterodorsal process (=maxillary process, = subnarial process): wide, plate-like (0); thin (1). Parrish 1993; Clark et al. 2000; Olsen et al. 2000; Benton and Walker 2002; Sues et al. 2003; Clark et al. 2004; Nesbitt 2009: 3

6) Premaxilla, posterodorsal process (=maxillary process, = subnarial process): extends posteriorly to the external naris (0); restricted to the ventral border of the external naris (1). Smith et al. 2007: 7; Irmis 2008: 13

7) Premaxilla, ventral process at the posterior end of the premaxillary body: absent (0); present (1). Langer and Benton 2006; Nesbitt 2009: 5

8) Premaxilla-nasal suture, on the internarial bar: V-shaped (0); W-shaped (1). Smith et al. 2007: 16; Irmis 2008: 17

9) Premaxillary teeth, number: 3(0); 4 (1); 5 (2); 6+ (3); 0 (4). Nesbitt and Norell 2006; Nesbitt 2009: 6

10) Premaxillary teeth, serrations: present (0); absent (1). Heckert et al. 1996; Parker 2007; Nesbitt 2009: 7

11) Premaxilla, teeth: present along entire length of the premaxilla (0); absent in the anterior portion of the premaxilla (1). Smith et al. 2007: 17; Irmis 2008: 139

12) Premaxilla, narial fossa: absent or shallow (0); expanded in the anteroventral corner of the naris (1). Sereno 1999; Langer and Benton 2006; Irmis et al. 2007; Nesbitt 2009: 9

13) Premaxilla-maxilla, subnarial gap between the elements: absent (0); present (1). Gauthier 1986; Langer and Benton 2006; Nesbitt 2009: 11

14) Premaxilla-maxilla, subnarial foramen between the elements: absent (0); present (1). Modified from Benton and Clark 1988; Parrish 1993; Juul 1994; Benton 1999; Nesbitt 2009: 12

15) Premaxilla-maxilla, subnarial foramen between the elements: the border of the foramen is present on both the maxilla and the premaxilla (0); the border of the foramen is present on the maxilla but not on the premaxilla (1); the border of the foramen is present on the premaxilla but not on the maxilla (2). Modified from Benton and Clark 1988; Parrish 1993; Juul 1994; Benton 1999; Nesbitt 2009: 12

16) Maxilla, facial portion anterior to anterior edge of antorbital fenestra: shorter than posterior portion (0); equal in length or longer than portion posterior to anterior edge of fenestra (1). Clark et al. 2000; Olsen et al. 2000; Benton and Walker 2002; Clark et al. 2004; Sues et al. 2003; Clark et al. 2004; Nesbitt 2009: 14

17) Maxillary teeth, posterior edge of posterior maxillary teeth: concave or straight (0); convex (1). Sues et al. 2003; Clark et al. 2004; Nesbitt 2009: 15

18) Maxillary tooth count: less than 12 (0); 12 - 18 (1); more than 18 (2). Smith et al. 2007: 3; Irmis 2008: 31

19) Maxilla, posterior extent of the maxillary tooth row: extends to approximately half of the anteroposterior length of the orbit (0); completely antorbital; tooth row ends anterior to the vertical strut of the lacrimal (1). Smith et al. 2007: 35; Irmis 2008:147

20) Maxilla, posterior process: articulates ventral to the jugal (0); articulates into a slot on the lateral side of the jugal (1). Nesbitt 2009: 16

21) Maxilla, dentition: present (0); absent (1). Nesbitt and Norell 2006; Nesbitt 2009: 18

22) Maxilla, buccal emargination separated from the ventral margin of the antorbital fossa: absent (0); present (1). Butler 2005, 2007; Irmis et al. 2006; Irmis et al., 2007; Nesbitt 2009: 23

23) Maxilla, anterodorsal margin: separated from the external naris by the premaxilla (0); borders the external naris (1). Gauthier 1986; Langer and Benton 2006; Nesbitt 2009: 24

24) Maxilla, anterodorsal margin at the base of the dorsal process: convex or straight (0); concave (1). Langer and Benton 2006; Nesbitt 2009: 25

25) Lateral surface of the maxilla: smooth (0); sharp longitudinal ridge present (1); bulbous longitudinal ridge present (2). Gower 1999; Weinbaum and Hungerbühler 2007; Nesbitt 2009: 26

26) Maxilla, depth of the ventral portion of the antorbital fossa: less than or subequal to the depth of the maxilla below the ventral margin of the antorbital fossa (0); much greater than the depth of the maxilla below the ventral margin of the antorbital fossa (1). Smith et al. 2007: 35; Irmis 2008: 27

27) Maxilla, posterior portion ventral to the antorbital fenestra: tapers posteriorly (0); has a similar dorsoventral depth as the anterior portion ventral to the antorbital fenestra (1); expands dorsoventrally at the posterior margin of the maxilla (2). Nesbitt 2009: 27

28) Maxilla, promaxillary foramen: absent (0); present (1). Rauhut 2003; Tykoski 2005; Smith et al. 2007: 32; Irmis 2008: 34; Nesbitt 2009: 28

29) Maxilla, dorsal (=ascending) process: tapers posterodorsally (0); remains the same width (1). Nesbitt 2009: 29

30) Antorbital fenestra, anterior margin: gently rounded (0); nearly pointed (1). Benton and Clark 1988; Benton and Walker 2002; Weinbaum and Hungerbühler 2007; Nesbitt 2009: 30

31) Maxilla, palatal processes: do not meet at the midline (0); meet at the midline (1). Parrish 1993; Clark et al. 2000; Olsen et al. 2000; Benton and Walker 2002; Sues et al. 2003; Clark et al. 2004; Nesbitt 2009: 32

32) Nasals, posterior portion at the midline: convex or flat (0); concave (1). Nesbitt 2009: 34

33) Nasal, dorsolateral margin of the anterior portion (just posterodorsal to the external naris): smoothly rounded (0); distinct anteroposteriorly ridge on the lateral edge (1). Nesbitt 2009: 35

34) Nasal: does not possess a posterolateral process that envelops part of the anterior ramus of the lacrimal (0); possesses a posterolateral process that envelops part of the anterior ramus of the lacrimal (1). Yates 2003; Langer and Benton 2006; Nesbitt 2009: 36

35) Nasal: does not form part of the dorsal border of the antorbital fossa (0); forms part of the dorsal border of the antorbital fossa (1). Sereno et al. 1994; Langer and Benton 2006; Irmis et al. 2007; Nesbitt 2009: 37

36) Lacrimal: does not fold over the posterior/posterodorsal part of the antorbital fenestra (0); folds over the posterior/posterodorsal part of the antorbital fenestra (1). Sereno 1999; Langer and Benton 2006; Nesbitt 2009: 38

37) Lacrimal, height: significantly less than the height of the orbit, and usually fails to reach the ventral margin of the orbit (0); as high as the orbit, and contacts the jugal at the level of the ventral margin of the orbit (1). Rauhut 2003; Nesbitt 2009: 39

38) Lacrimal, orientation of long axis: sloping anterodorsally (0); erect or nearly vertical (1). Smith et al. 2007:60; Irmis 2008: 47

39) Lacrimal 'horn': absent (0); present, forms dorsal crest above the orbit (1). Smith et al. 2007: 52; Irmis 2008: 45

40) Lacrimal fenestra: absent (0); present (1). Smith et al. 2007: 51; Irmis 2008: 44

41) Frontal, dorsal surface: flat (0); with longitudinal ridge along midline (1). Wu and Chatterjee 1993; Clark et al. 2000; Olsen et al. 2000; Benton and Walker 2002; Sues et al. 2003; Clark et al. 2004; Nesbitt 2009: 42

42) Frontal, anterior portion: about as wide as the orbital margin or has a transversely aligned suture with the nasal (0); tapers anteriorly along the midline (1). Nesbitt 2009: 43

43) Postfrontal: present (0); absent (1). Gauthier 1986; Benton and Clark 1988; Juul 1994; Bennett 1996; Novas 1996; Benton 1999; Clark et al. 2000; Olsen et al. 2000; Benton and Walker 2002; Sues et al. 2003; Clark et al. 2004; Langer and Benton 2006; Nesbitt 2007; Irmis et al., 2007; Nesbitt 2009: 44

44) Quadratojugal: forms less than 80% of the posterior border of the lower temporal fenestra (0); more than 80% of the posterior border of the lower temporal fenestra (1). Benton and Clark 1988; Parrish 1993; Nesbitt 2009: 45

45) Squamosal, posterior end: does not extend posterior to the head of the quadrate (0); extends posterior to the head of the quadrate (1). Nesbitt 2009: 48

46) Squamosal: without distinct ridge on dorsal surface along edge of supratemporal fossa (0); with distinct ridge on dorsal surface along edge of supratemporal fossa (1). Bonaparte 1982; Parrish 1993; Nesbitt 2009: 49

47) Squamosal, facet for the paroccipital process on the medial side of the posterior process: mediolaterally thin (0); rounded and thick (1). Nesbitt 2009: 54

48) Squamosal, ventral process: wider than one quarter of its length (0); narrower than one quarter of its length (1). Yates 2003; Langer and Benton 2006; Nesbitt 2009: 56

49) Parietals, upper temporal fenestrae separated by: broad, flat area (0); supratemporal fossa separated by a mediolaterally thin strip of flat bone (1); has supratemporal fossa separated by a "sagittal crest" (which may be divided by interparietal suture) (2). Clark et al. 2000; Olsen et al. 2000; Benton and Walker 2002; Sues et al. 2003; Clark et al. 2004; Nesbitt 2009: 59

50) Postorbital, ventral termination of the ventral process: tapered (0); blunt (1). Benton and Clark 1988; Juul 1994; Benton 1999; Alcober 2000; Benton and Walker 2002; Nesbitt 2009: 65

51) Postorbital-squamosal, contact: restricted to the dorsal margin of the elements (0); continues ventrally for much or most of the ventral length of the squamosal (1). Nesbitt 2009: 66

52) Postorbital bar: composed both of the jugal and postorbital in nearly equal proportions (0); composed by mostly the postorbital (1). Nesbitt 2009: 67

53) Jugal, anterior extent of the slot for the quadratojugal: well posterior of the posterior edge of the dorsal process of the jugal (0) at or anterior to the posterior edge of the dorsal process of the jugal (1). Nesbitt 2009: 68

54) Jugal, anterior process: participates in posterior edge of antorbital fenestra (0); excluded from the antorbital fenestra by lacrimal or maxilla (1). Clark et al. 2000; Olsen et al. 2000; Benton and Walker 2002; Sues et al. 2003; Clark et al. 2004; Rauhut 2003; Langer and Benton 2006; Nesbitt 2009: 69

55) Jugal, posterior process: lies dorsal to the anterior process of the quadratojugal (0); ventral to the anterior process of the quadratojugal (1); splits the anterior process of the quadratojugal (2); is split by the anterior process of the quadratojugal (3). Nesbitt 2009: 71

56) Jugal, posterior termination: anterior to or at the posterior extent of the lower temporal fenestra (0); posterior to the lower temporal fenestra (1). Nesbitt 2009: 72

57) Jugal, long axis of the body: nearly horizontal (0); anterodorsally inclined (1). Heckert and Lucas 1999; Parker 2007; Nesbitt 2009: 74

58) Jugal, longitudinal ridge on the body: absent (0); present (1). Modified from Nesbitt 2009: 75

59) Jugal, longitudinal ridge on the body: sharp (0); rounded and broad (1); rounded and restricted to a bulbous ridge (2). Modified from Nesbitt 2009: 75

60) Quadrate, head: partially exposed laterally (0); completely covered by the squamosal (1). Sereno and Novas 1994; Juul 1994; Novas 1996; Benton 1999; Langer and Benton 2006; Nesbitt 2009: 78

61) Quadratojugal and quadrate, suture between the elements, foramen: present (0); absent (1). Parrish 1991; Benton and Walker 2002; Nesbitt 2009: 79

62) Quadrate, angled: posteroventrally or vertical (0); anteroventrally (1). Nesbitt 2007; Nesbitt 2009: 82

63) Pterygoid-ectopterygoid, articulation: ectopterygoid ventral to pterygoid (0); ectopterygoid dorsal to pterygoid (1). Sereno and Novas 1994; Novas 1996; Benton 1999; Irmis et al. 2007; Nesbitt 2009: 84

64) Ectopterygoid, ventral recess: absent (0); present (1). Gauthier 1986; Langer and Benton 2006; Nesbitt 2009: 86

65) Ectopterygoid, body: arcs anteriorly (0); arcs anterodorsally (1). Nesbitt 2009: 87

66) Ectopterygoid: single-headed (0); double-headed (1). Weinbaum and Hungerbühler 2007; Nesbitt 2009: 89

67) Basipterygoid, processes directed: anteriorly or ventrally at their distal tips (0); posteriorly at their distal tips (1). Nesbitt 2009: 93

68) Parabasisphenoid, foramina for entrance of cerebral branches of internal carotid artery into the braincase positioned on the surface: ventral (0); lateral (1). Parrish 1993; Gower and Sennikov 1996; Gower 2002; Nesbitt 2009: 95

69) Parabasisphenoid, plate: present (0); absent (1). Modified from Gower and Sennikov 1996; Nesbitt 2009: 96

70) Parabasisphenoid, plate: straight (0); arched anteriorly (1). Modified from Gower and Sennikov 1996; Nesbitt 2009: 96

71) Parabasisphenoid, semilunar depression on the lateral surface of the basal tubera: present (0); absent (1). Gower and Sennikov 1996; Nesbitt 2009: 98 71 included ? Mk1 (est.)

72) Parabasisphenoid, recess (=median pharyngeal recess of some authors = hemispherical sulcus = hemispherical fontanelle): absent (0); present (1). Nesbitt and Norell 2006; Nesbitt 2009: 100

73) Parabasisphenoid, anterior tympanic recess on the lateral side of the braincase: absent (0); present (1). Makovicky and Sues 1998; Rauhut 2003; Nesbitt 2009: 101

74) Parabasisphenoid, between basal tubera and basipterygoid processes: approximately as wide as long or wider (0); significantly elongated at least 1.5 times longer than wide(1). Rauhut 2003; Nesbitt 2007; Nesbitt 2009: 103

75) Basioccipital, portion of the basal tubera: rounded and anteroposteriorly elongated (0); blade-like and anteroposteriorly shortened (1). Nesbitt 2009: 106

76) Opisthotic, paroccipital processes: no or slight dorsal and ventral expansion distally (0); markedly expanded dorsally at the distal ends (1). Clark et al. 2000; Olsen et al. 2000; Benton and Walker 2002; Sues et al. 2003; Clark et al. 2004; Nesbitt 2009: 108

77) Opisthotic, paroccipital processes: directed laterally or dorsolaterally (0); directed ventrolaterally (1). Rauhut 2003; Hwang et al. 2004; Smith et al. 2007: 90; Irmis 2008: 89; Nesbitt 2009: 110

78) Paroccipital processes, ventral rim of the bases: above or level with the dorsal border of the occipital condyle (0); situated at mid-height of occipital condyle or lower (1). Smith et al. 2007: 91

79) Opisthotic, ventral ramus (= crista interfenestralis): extends further laterally or about the same as lateralmost edge of exoccipital in posterior view (0); covered by the lateralmost edge of exoccipital in posterior view (1). Gower 2002; Nesbitt 2009: 111

80) Exoccipital, lateral surface: without subvertical crest (= metotic strut) (0); with clear crest (= metotic strut) (1). Modified from Gower 2002; Nesbitt 2009: 114

81) Exoccipital, lateral surface: clear crest (= metotic strut) lying anterior to both external foramina for hypoglossal nerve (XII)(0); clear crest (= metotic strut) present anterior to the more posterior external foramina for hypoglossal nerve (XII) (1). Gower 2002; Nesbitt 2009: 114

82) Exocciptials: meet along the midline on the floor of the endocranial cavity (0); do not meet along the midline on the floor of the endocranial cavity (1). Gower and Sennikov 1996; Gower 2002; Nesbitt 2009: 115

83) Vestibule, medial wall: incompletely ossified (0); almost completely ossified (1). Gower 2002; Nesbitt 2009: 117

84) Lagenar/cochlea recess: absent or short and strongly tapered (0); present and elongated and tubular (1). Gower 2002; Nesbitt 2009: 118

85) Supraoccipital: excluded from dorsal border of foramen magnum by mediodorsal midline contact between opposite exoccipitals (0); contributes to border of foramen magnum (1). Gower 2002; Nesbitt 2009: 126

86) Foramen for trigeminal nerve and middle cerebral vein: combined and undivided (0); at least partially subdivided by prootic (1); fully divided (2). Gower and Sennikov 1996; Gower 2002; Nesbitt 2009: 131

87) Foramen or groove passing above and into the dorsal end of the metotic foramen: absent (0); present (1). Gower 2002; Nesbitt 2009: 132

88) Auricular recess: largely restricted to prootic (0); extends onto internal surface of epiotic/supraoccipital (1). Gower 2002; Nesbitt 2009: 133

89) Skull length: less than 50% of length of the presacral vertebral column (0); more than 50% of length of the presacral vertebral column (1). Sereno 1991; Benton 1999; Nesbitt 2009: 134

90) Skull length: longer than two-thirds of the femoral length (0); shorter than two-thirds of the femoral length (1). Gauthier 1986; Langer and Benton 2006; Nesbitt 2009: 135

91) Antorbital fossa: restricted to the lacrimal and dorsal process of the maxilla (0); present on the lacrimal, dorsal process of the maxilla and the dorsolateral margin of the posterior process of the maxilla (the ventral border of the antorbital fenestra) (1). Nesbitt 2009: 137

92) Post-temporal opening, size: equal or greater than half the diameter of the foramen magnum (0); less than half the diameter of the foramen magnum or absent (1). Sereno and Novas 1994; Novas 1996; Benton 1999; Nesbitt 2009: 141

93) Orbit, shape: circular or elliptical (0); tall and narrow (the "'keyhole-shaped orbit"'; maximum width is less than half the maximum height)(1); with distinct ventral point surrounded by V-shaped dorsal processes of jugal (2). Benton and Clark 1988; Parrish 1993; Gower 2000; Benton and Walker 2002; Nesbitt 2009: 142

94) Supratemporal fossa: absent anterior to the supratemporal fenestra (0); present anterior to the supratemporal fenestra (1). Gauthier 1986; Novas 1996; Nesbitt 2009: 144

95) Postparietal(s): present (0); absent (1). Juul 1994; Bennett 1996; Dilkes 1998; Nesbitt 2009: 146

96) Palpebral(s): absent (0); present (1). Nesbitt 2009: 147

97) Predentary: absent (0); present (1). Sereno 1986; Butler et al. 2007, 2008; Irmis et al. 2007; Nesbitt 2009: 151

98) Anterior half of the dentary, position of the Meckelian groove: dorsoventral center of the dentary (0); restricted to the ventral border (1). Nesbitt 2009: 152

99) Dentary, anterior extent of the Meckelian groove: ends well short of the dentary symphysis (0); present through the dentary symphysis (1). Nesbitt 2009: 153

100) Dentary, dorsal margin of the anterior portion compared to the dorsal margin of the posterior portion of the dentary: horizontal (about in the same plane)(0); ventrally deflected (1); dorsally expanded (2). Nesbitt 2009: 154

101) Dentary, anterior extremity: rounded (0); tapers to a sharp point (1). Nesbitt 2009: 155

102) Articular: without dorsomedial projection posterior to the glenoid fossa (0); with dorsomedial projection separated from glenoid fossa by a clear concave surface (1); with dorsomedial projection continuous with the glenoid fossa (2). Clark et al. 2000; Olsen et al. 2000; Benton and Walker 2002; Sues et al. 2003; Clark et al. 2004; Nesbitt 2009: 156

103) Articular, ventromedially directed process: absent (0); present (1). Nesbitt 2009: 157

104) Articular, glenoid of the mandible located: level with dorsal margin of the dentary (0); well ventral of the dorsal margin of the dentary (1). Gauthier 1986; Langer and Benton 2006; Nesbitt 2009: 158

105) Articular, foramen on the medial side: absent (0); present and medial to the glenoid (1). Nesbitt 2009: 159

106) Surangular, ridge or process on lateral surface, anterior to jaw suture: absent (0); present, strong anteroposteriorly extended ridge (1). Butler et al. 2008; Irmis 2008: 126

107) Coronoid process, dorsally expanded: absent (0); present (1). Sereno 1986; Sereno 1999; Butler 2005; Butler et al. 2008; Irmis et al. 2007; Nesbitt 2009: 161

108) Mandibular fenestra: anteroposterior length more than maximum depth of dentary ramus but less than half the length of the mandible (0); greater than half the length of the mandible (1); reduced, anteroposterior length less than maximum depth of dentary ramus (2).Butler 2005; Nesbitt and Norell 2006; Nesbitt 2009: 162

109) Splenial, foramen in the ventral part: absent (0); present (1). Rauhut 2003; Langer and Benton 2006; Smith et al. 2007; Nesbitt 2009: 165

110) Dentary teeth: present (0); absent (1). Modified from Parrish 1994; Parker 2007; Nesbitt 2009: 166

111) Dentary teeth present: along entire length of the dentary (0); but absent in the anterior portion (1). Modified from Parrish 1994; Parker 2007; Nesbitt 2009: 166

112) Dentition: generally homodont (0); markedly heterodont (1). Parrish 1993; Nesbitt 2009: 167

113) Tooth, serrations: present as small fine knife-like serrations (0); present and enlarged and coarser (lower density) = denticles (1). Gauthier et al. 1988; Juul 1994; Dilkes 1998; Irmis et al. 2007; Nesbitt 2009: 168

114) Extensive planar wear facets across multiple maxillary/dentary teeth: absent (0); present (1). Weishampel and Witmer 1990; Irmis et al 2007; Nesbitt 2009: 169

115) Medial or lateral overlap of adjacent crowns in maxillary and dentary teeth: absent (0); present (1). Sereno 1986; Butler et al. 2008; Nesbitt 2009: 170

116) Tooth, crown: not mesiodistally expanded (0); mesiodistally expanded above root in cheek teeth (1). Sereno 1986; Irmis et al 2007; Butler et al. 2008; Nesbitt 2009: 171

117) Moderately developed lingual expansion of crown (=cingulum) on maxillary/dentary teeth: absent (0); present (1). Sereno 1986; Butler et al. 2008: Nesbitt 2009: 172

118) Maxillary and dentary crowns, shape: apicobasally tall and blade-like (0); apicobasally short and subtriangular (1). Sereno 1986; Butler et al. 2008; Nesbitt 2009: 173

119) Tooth, implantation: teeth fused to the bone of attachment at the base (0); free at the base of the tooth (1). Gauthier 1984; Benton and Clark 1988; Benton 1990; Bennett 1996; Nesbitt 2009: 174

120) Pterygoid, teeth, on palatal process of the pterygoid: present (0); absent (1). Juul 1994; Gower and Sennikov 1997; Nesbitt 2009: 175

121) Postaxial intercentra: present (0); absent (1). Gauthier 1984; Benton and Clark 1988; Sereno 1991a; Parrish 1993; Juul 1994; Bennett 1996; Nesbitt 2009: 177

122) Atlantal articulation facet in axial intercentrum, shape: saddle-shaped (0); concave with upturned lateral borders (1). Gauthier 1986; Langer and Benton 2006; Nesbitt 2009: 178

123) Axis, dorsal margin of the neural spine: expanded posterodorsally (0); arcs dorsally, where the anterior portion height is equivalent to the posterior height (1). Nesbitt 2009: 179

124) Axis, neural spine: sheet-like (0); anteroposteriorly reduced and rod-like (1). Smith et al. 2007: 145; Irmis 2008: 157

125) Anterior cervical vertebrae, prezygapophyses: transverse distance between medial margins of prezygapophyses less than mediolateral width of the neural canal (0); medial margin of prezygapophyses situated lateral to the margins of the neural canal (1). Smith et al. 2007: 156; Irmis 2008: 163

126) Cervical vertebrae, 3-5 centrum length: shorter or the same length as the mid-dorsal (0); longer than mid-dorsal (1). Sereno 1991; Nesbitt 2009: 181

127) Cervical vertebrae, deep recesses on the anterior face of the neural arch, lateral to the neural canal (=prechonos of Welles 1984): absent (0); present (1). Nesbitt 2009: 182

128) Third cervical vertebra, centrum length: subequal to the axis centrum (0); longer than the axis centrum (1). Gauthier 1986; Langer and Benton 2006; Nesbitt 2009: 183

129) Anterior to middle cervical vertebrae, diapophysis and parapophysis: well separated (0); nearly touching (1). Nesbitt 2009: 184

130) Anterior cervical vertebrae, neural arch, posterior portion ventral to the postzygapophysis: smooth posteriorly or has a shallow fossa (0); with a deep excavation with a thin bone lamina covering the anterior extent on the posterolateral surface (1). Langer and Benton 2006; Nesbitt 2009: 185

131) Epipophyses: absent in post-axial anterior cervical vertebrae (0); present in post- axial anterior cervical vertebrae (1). Gauthier 1986; Novas 1996; Langer and Benton 2006; Nesbitt 2009: 186

132) Epipophyses: absent in posterior cervical vertebrae (cervicals 6-9) (0); present in posterior cervical vertebrae (cervicals 6-9) (1). Sereno et al. 1993; Langer and Benton 2006; Nesbitt 2009: 187

133) Cervical vertebrae, pneumatic features (=pleurocoels) in the anterior portion of the centrum: absent (0); present (1). Modified from Holtz 1994; Rauhut 2003; Smith et al. 2007: 149; Irmis 2008: 162; Nesbitt 2009: 188

134) Cervical vertebrae, pneumatic features (=pleurocoels) in the anterior portion of the centrum: deep fossae (0); foramina (1). Modified from Holtz 1994; Rauhut 2003; Smith et al. 2007: 149; Irmis 2008: 162; Nesbitt 2009: 188

135) Cervical vertebrae, rimmed depression on the posterior part of the centrum: absent (0); present (1). Gauthier 1986; Rauhut 2003; Nesbitt 2009: 189

136) Cervical vertebrae, middle portion of the ventral keel: dorsal to the ventralmost extent of the centrum rim (0); ventral to the centrum rims (1). Nesbitt 2009: 190

137) Cervical vertebrae, distal end of neural spines: expansion absent (0); present (1). Modified from Gauthier 1984; Juul 1994; Nesbitt 2009: 191

138) Cervical vertebrae, distal end of neural spines: laterally expanded in the middle of the anteroposterior length (0); expanded anteriorly so that the spine table is triangular or heart-shaped in dorsal view (1). Modified from Gauthier 1984; Juul 1994; Nesbitt 2009: 191

139) Posterior cervical and/or dorsal vertebrae, hyposphene-hypantrum accessory intervertebral articulations: absent (0); present (1). Gauthier 1986; Juul 1994; Benton 1999; Rauhut 2003; Langer and Benton 2006; Weinbaum and Hungerbühler 2007; Nesbitt 2009: 195 140

140) Cervical ribs: slender and elongated (0); short and stout (1). Gauthier 1986; Benton and Clark 1988; Juul 1994; Benton 1999; Nesbitt 2009: 196

141) Cervical ribs, pneumatic excavations in head: absent (0); present (1). Smith et al. 2007: 165; Irmis 2008: 170

142) Dorsal vertebrae, deep fossae and/or pneumatopores ("pleurocoels"): absent (0); present in anterior dorsals (1). Smith et al. 2007: 170; Irmis 2008: 172

143) Anterior dorsal vertebrae, ventral keel: absent or very poorly developed (0); pronounced (1). Smith et al. 2007: 170; Irmis 2008: 172

144) Dorsal vertebrae, neural spine distal expansion: absent (0); present (1). Modified from Nesbitt 2009: 197

145) Dorsal vertebrae, neural spine distal expansion: with a flat dorsal margin (0); with a rounded dorsal margin (1). Modified from Nesbitt 2009: 197

146) Middle dorsal vertebrae, diapophyses and parapophyses: close to the midline (0); expand on stalks (1). Nesbitt 2009: 199

147) Sacral centra: separate (0); co-ossified at the ventral edge (1). Nesbitt 2009: 200

148) Sacral vertebrae, prezygapophyses and complimentary postzygapophyses: separate (0); co-ossified (1). Nesbitt 2009: 201

149) Primordial sacral one, sacral rib: doesn't or weakly articulates with anteriorly directed process (=preacetabular process) of the ilium (0); articulates with the anteriorly directed process of the ilium (1). Nesbitt 2005; 2007; Nesbitt 2009: 202

150) Sacral vertebrae, centra articular rims: present in sacrum (0); nearly obliterated (1). Nesbitt 2007; Nesbitt 2009: 204

151) Trunk vertebrae: free from the sacrum (0); incorporated into the sacrum, with their ribs/transverse processes articulating with the pelvis (1). Sereno et al. 1993; Langer and Benton 2006; Nesbitt 2009: 205

152) Caudal vertebrae: free from the sacrum (0); incorporated into the sacrum, with their ribs/transverse processes articulating with the pelvis (1). Galton 1976; Langer and Benton 2006; Nesbitt 2009: 206

153) Insertion of a sacral vertebra between the first and second primordial sacral vertebrae: absent (0); present (1). Nesbitt 2009: 207

154) Sacral ribs: almost entirely restricted to a single sacral vertebra (0); shared between two sacral vertebrae (1). Nesbitt 2009: 208

155) First primordial sacral, articular surface of sacral rib: circular (0); C- shaped in lateral view (1). Langer and Benton 2006; Nesbitt 2009: 209

156) Middle caudal vertebrae, accessory laminar process on anterior face of neural spine: absent (0); present (1). Benton and Clark 1988; Juul 1994; Benton 1999; Benton and Walker 2002; Rauhut 2003; Irmis et al. 2007; Nesbitt 2009: 210

157) Distal caudal vertebrae, prezygapophyses: not elongated (0); elongated more than a quarter of the adjacent centrum (1). Gauthier 1986; Rauhut 2003; Nesbitt 2007; Nesbitt 2009: 211

158) Forelimb-hindlimb length ratio: more than 0.55 (0); less than 0.55 (1). Gauthier 1984; Sereno 1991; Juul 1994; Benton 1999; Nesbitt 2009: 212

159) Clavicles: present (0); absent (1). Modified from Gauthier 1986; Sereno 1991; Benton 1999; Benton and Walker 2002; Nesbitt 2009: 213

160) Clavicles: unfused (0); fused into a furcula (1). Modified from Gauthier 1986; Sereno 1991; Benton 1999; Benton and Walker 2002; Nesbitt 2009: 213

161) Interclavicle: present (0); absent (1). Gauthier 1986; Sereno 1991; Juul 1994; Benton 1999; Nesbitt 2009: 214

162) Scapula, entire anterior margin: straight/convex or partially concave (0); markedly concave (1). Gower and Sennikov 1997; Nesbitt 2009: 217

163) Scapula, blade height versus distal width: less than 3 times distal width (0); more than 3 times distal width (1). Sereno 1999; Nesbitt 2009: 218

164) Scapulocoracoid, anterior margin: distinct notch between the two elements (0); uninterrupted edge between the two elements (1). Parrish 1993; Benton 1999; Nesbitt 2009: 221

165) Coracoid: subcircular in lateral view (0); with post-glenoid process (notch ventral to glenoid) (1). Clark et al. 2004; Nesbitt 2009: 222

166) Coracoid, post-glenoid process: short (0); elongate and expanded posteriorly only (1). Clark et al. 2004; Nesbitt 2009: 223

167) Coracoid, posteroventral portion: smooth (0); possesses a "swollen" tuber (=biceps tuber) (1). Nesbitt 2009: 225

168) Glenoid, orientation: posterolaterally (0); directed posteroventrally (1). Fraser et al. 2002; Nesbitt 2009: 227

169) Humerus, apex of deltopectoral crest situated at a point corresponding to: less than 30% down the length of the humerus (0); more than 30% down the length of the humerus (1). Bakker and Galton 1974; Benton 1990; Juul 1994; Novas 1996; Benton 1999; Nesbitt 2009: 230

170) Humerus, length: longer than or subequal to 0.6 of the length of the femur (0); shorter than 0.6 of the length of the femur (1). Novas 1993; Langer and Benton 2006; Nesbitt 2009: 231

171) Humerus, proximal head: confined to the proximal surface (0); posteriorly expanded and hooked (1). Nesbitt 2009: 232

172) Humerus, proximal articular surface: continuous with the deltopectoral crest (0); separated by a gap from the deltopectoral crest (1). Nesbitt, 2009: 233

173) Humerus, ectepicondylar flange: present (0); absent (1). Nesbitt 2009: 234

174) Humerus, distal end width: narrower or equal to 30% of humerus length (0); greater than 30% of humerus length (1). Langer and Benton 2006; Nesbitt 2009: 235

175) Ulna, lateral tuber (=radius tuber) on the proximal portion: absent (0); present (1). Nesbitt 2009: 237

176) Ulna, distal end in posterolateral view: rounded and convex (0); squared off where the distal surface is nearly flat (1). Nesbitt 2009: 238

177) Ulna, distal end: anteroposteriorly compressed or oval-shaped (0); with anterior expansion (1). Nesbitt 2009: 239

178) Radius, distal end: convex (0); shallow longitudinal groove on the posterior side (1). Nesbitt 2009: 240

179) Radius, length: longer than 80% of humerus length (0); shorter than 80% of humerus length (1). Langer and Benton 2006; Nesbitt 2009: 241

180) Pteroid bone: absent (0); present (1). Bennett 1996; Nesbitt 2009: 244

181) Longest metacarpal: longest metatarsal >.5 (0); <.5 (1). Nesbitt 2009: 245

182) Metacarpals, proximal ends: overlap (0); abut one another without overlapping (1). Clark et al. 2000; Olsen et al. 2000; Benton and Walker 2002; Sues et al. 2003; Clark et al. 2004; Nesbitt 2009: 246

183) Manual length (measured as the average length of digits I-III) accounts for: less than 0.3 of the total length of humerus plus radius (0); more than 0.3 but less than 0.4 of the total length of humerus plus radius (1); more than 0.4 of the total length of humerus plus radius (2). Gauthier 1986; Langer and Benton 2006; Nesbitt 2009: 247

184) Medialmost distal carpal: subequal other distal carpals (0); significantly larger than other distal carpals (1). Gauthier 1986; Langer and Benton 2006; Nesbitt 2009: 248

185) Distal carpal V: present (0); absent (1). Sereno 1999; Langer and Benton 2006; Nesbitt 2009: 249

186) Extensor pits on the proximodorsal portion of metacarpals I-III: absent or shallow and symmetrical (0); deep and asymmetrical (1). Sereno et al. 1993; Langer and Benton 2006; Nesbitt 2009: 250

187) Metacarpal I, width at the middle of the shaft accounts for: less than 0.35 of the total length of the bone (0); more than 0.35 of the total length of the bone (1). Bakker and Galton 1974; Langer and Benton 2006; Nesbitt 2009: 251

188) Digit I with metacarpal: longer than the ungual (0); subequal or shorter than the ungual (1). Sereno 1999; Langer and Benton 2006; Nesbitt 2009: 252

189) Manual digit I, first phalanx: is not the longest non-ungual phalanx of the manus (0); is the longest non-ungual phalanx of the manus (1). Gauthier 1986; Langer and Benton 2006; Nesbitt 2009: 253

190) Metacarpal I, distal condyles: approximately aligned or slightly offset (0); lateral condyle strongly distally expanded relative to medial condyle (1). Bakker and Galton 1974, Langer and Benton 2006; Irmis et al., 2007; Nesbitt 2009: 254

191) Metacarpal II: shorter than metacarpal III (0); equal to or longer than metacarpal III (1). Gauthier 1986; Langer and Benton 2006; Irmis et al. 2007; Nesbitt 2009: 256

192) Manual digits I-III: blunt unguals on at least digits II and III (0); trenchant unguals on digits I-III (1). Gauthier 1986; Juul 1994; Benton 1999; Irmis et al. 2007; Nesbitt 2009: 257

193) Manual digit IV: five phalanges (0); four phalanges (1); three or two phalanges (2); one phalanx (3). Gauthier 1986; Benton and Clark 1988; Novas 1996; Benton 1999; Irmis et al. 2007; Nesbitt 2009: 258

194) Metacarpal IV: present (0); reduced to a nubin or absent (1). Gauthier 1986; Nesbitt 2009: 259

195) Metacarpal IV, shaft width: about the same width than that of metacarpals I-III (0); significantly narrower than that of metacarpals I-III (1). Sereno et al. 1993; Langer and Benton 2006; Nesbitt 2009: 261

196) Manual digit IV length: less than or equal to 50% of total forelimb length (0); more than 50% of total forelimb length (1). Bennett 1996; Irmis et al. 2007; Nesbitt 2009: 262

197) Manual digit V: possesses one or more phalanges (0); lacks phalanges (1); absent or reduced to a tiny nubbin (2). Bakker and Galton 1974; Langer and Benton 2006; Irmis et al. 2007; Nesbitt 2009: 263

198) Ilium, supra-acetabular crest (=supra-acetabular rim): projects laterally or ventrolaterally (0); projects ventrally (1). Gauthier 1986; Nesbitt 2009: 264

199) Ilium, distal extent of the supra-acetabular crest (=supra-acetabular rim): near or at the articular facet for the pubis (0); ends well proximal of the facet for the pubis (1). Ezcurra and Brusatte 2011: 190

200) Ilium, crest dorsal to the supra-acetabular crest: absent (0); present (1). Modified from Nesbitt 2009: 265

201) Ilium, crest dorsal to the supra-acetabular crest: divides the anterior (=preacetabular) process from the posterior (=postacetabular) process (0); confluent with anterior extent of the anterior (=preacetabular) process of the ilium (1). Modified from Nesbitt 2009: 265

202) Ilium, anterior (=preacetabular, =cranial) process: short and does not extend anterior of the acetabulum (0); long and extends anterior of the acetabulum (1). Nesbitt 2009: 269

203) Ilium, shape of the anterior (=preacetabular, =cranial) process: blunt and tapering dorsally (0); square-shaped (1). Yates 2007: 247; Irmis 2008: 239

204) Ilium, posterior margin of the postacetabular process in lateral view: straight or convex (0); notched or indented (1). Smith et al. 2007: 262; Irmis 2008: 255

205) Ilium, orientation: mainly vertically orientated (0-20°) (0); ventrolaterally deflected about 45°(1). Benton and Clark 1988; Juul 1994; Benton and Walker 2002; Nesbitt 2009: 270

206) Ilium, distinct fossa present for the attachment of the m caudifemoralis brevis: absent (0); present (1). Modified from Gauthier and Padian 1985; Gauthier 1986: Juul 1994; Novas 1996; Benton 1999; Hutchinson 2001; Nesbitt 2009: 271

207) Ilium, distinct fossa present for the attachment of the m caudifemoralis brevis: present as an embankment on the lateral side of the posterior portion of the ilium (0); present as a deep fossa on the ventral surface of postacetabular part of the ilium (1). Modified from Gauthier and Padian 1985; Gauthier 1986: Juul 1994; Novas 1996; Benton 1999; Hutchinson 2001; Nesbitt 2009: 271

208) Ilium, ridge connecting the posterior portion of the supra-acetabular rim to the posterior portion of the ilium: absent (0); present (1). Langer and Benton 2006; Nesbitt 2009: 272

209) Ilium, ventral margin of the acetabulum: convex (0); straight (1); concave (2). Bakker and Galton 1974; Gauthier and Padian 1985; Gauthier 1986; Juul 1994; Novas 1996; Benton 1999; Benton et al. 2000; Fraser et al. 2002; Langer and Benton 2006; Nesbitt 2009: 273

210) Ilium, acetabular antitrochanter: absent (0); present (1). Sereno and Arcucci 1994; Novas 1996; Benton 1999; Fraser et al. 2002; Irmis et al. 2007; Nesbitt 2009: 274

211) Ilium, dorsal margin dorsal to the supra-acetabular rim: rounded, or blade-like (0); flat (1). Nesbitt 2009: 275

212) Ilium, dorsal portion: height about the same or shorter than the dorsal portion of the supra-acetabular rim to the pubis-ischium contact (0); expanded dorsally, height markedly taller than the dorsal portion of the supra-acetabular rim to the pubis-ischium contact (1). Nesbitt 2009: 276

213) Ilium, ischiadic peduncle orientation: mainly vertical in lateral aspect (0); well expanded posteriorly to the anterior margin of the postacetabular embayment (1). Langer and Benton, 2006; Nesbitt 2009: 277

214) Pubis, length: less than 70% of femoral length (0); more than 70% or more of femoral length (1). Novas 1996; Nesbitt 2009: 278

215) Pubis, orientation: anteroventral (0); rotated posteroventrally to lie alongside the ischium (opisthopubic) (1). Sereno 1986; Butler et al. 2008b; Nesbitt 2009: 279

216) Pubis, prepubic process: absent, anterior margin unexpanded (0); present, anterior margin expanded into a process (1). Sereno 1986; Butler et al. 2008b; Nesbitt 2009: 280

217) Pubis, length: shorter or subequal to the ischium (0); longer than ischium (1). Benton and Clark 1988; Juul 1994; Novas 1996; Benton 1999; Benton and Walker 2002; Nesbitt 2009: 282

218) Pubis, distal end: unexpanded (0); expanded relative to the shaft (=pubis boot) (1). Gauthier 1986; Sereno and Novas 1992; Juul 1994; Benton 1999; Rauhut 2003; Langer and Benton 2006; Nesbitt 2007; Nesbitt 2009: 283

219) Pubis, expanded distal margin: mediolaterally thick and rounded (0); mediolaterally thin (1). Gauthier 1986; Juul 1994; Benton 1999; Nesbitt 2009: 284

220) Pubis, expanded distal margin: shorter than 33% of the length of the shaft of the pubis (0); greater than 33% of the length of the shaft of the pubis (1). Nesbitt and Norell 2006; Nesbitt 2007; Nesbitt 2009: 285

221) Proximal portion of the pubis: articular surfaces with the ilium and the ischium continuous (0); articular surfaces with the ilium and the ischium separated by a groove or gap (1). Nesbitt 2009: 286

222) Ischio-pubis contact: present (0); absent (1). Modified from Benton and Clark 1988; Novas 1996; Nesbitt 2009: 287

223) Ischio-pubis contact: extended ventrally (0); reduced to a thin proximal contact (1). Modified from Benton and Clark 1988; Novas 1996; Nesbitt 2009: 287

224) Pubis, pubic apron, proximal portion: similar anteroposterior thickness as the rest of the pubic apron (0); thickened process (1). Nesbitt 2005; 2007; Nesbitt 2009: 288

225) Pubis, mediolateral width of distal portion: nearly as broad as proximal width (0); significantly narrower than proximal width (1); mediolaterally compressed and not broader than anteroposteriorly deep (2). Galton 1976; Novas 1996; Sereno 1999; Langer and Benton 2006; Nesbitt 2009: 289

226) Ischium, medial contact with antimere: restricted to the medial edge (0); extensive contact but the dorsal margins are separated (1); extensive contact and the dorsal margins contact each other (2). Nesbitt 2009: 291

227) Ischium, cross-section of the distal portion: thin, plate-like (0); rounded or elliptical (1); sub-triangular (2). Sereno 1999; Langer and Benton 2006; Irmis et al. 2007; Nesbitt 2009: 293

228) Ischium, distal portion: unexpanded (0); expanded relative to the ischial shaft (=ischial boot) (1). Smith and Galton 1990; Holtz 1994; Hutchinson 2001; Rauhut 2003; Langer and Benton 2006; Nesbitt 2009: 294

229) Ischium, obturator process: confluent with the pubic peduncle (0); offset from the pubic peduncle by a notch (1). Gauthier 1986; Novas 1993; Rauhut 2003; Nesbitt 2009: 295

230) Ischium, ventral margin: continuous ventral margin (0); notch present (1). Sereno et al. 1996; Rauhut 2003; Nesbitt 2009: 296

231) Ischium, proximal articular surfaces: articular surfaces with the ilium and the pubis continuous (0); articular surfaces with the ilium and the pubis continuous but separated by a fossa (1); articular surfaces with the ilium and the pubis separated by a large concave surface (2). Irmis et al. 2007; Nesbitt 2009: 297

232) Ischium, groove on the dorsolateral surface of the proximal part: absent (0); present (1). Irmis et al 2007: 75; Irmis 2008: 275

233) Ischium length: about the same length or shorter than the dorsal margin of the iliac blade (minus the anterior process) (0); markedly longer than the dorsal margin of iliac blade (minus the anterior process) (1). Juul 1994; Nesbitt 2009: 298

234) Tibia (or fibula)-femur length: femur longer or about the same length as the tibia/fibula (0); tibia longer (1). Gauthier 1986; Sereno 1991a; Juul 1994; Benton 1999; Irmis et al. 2007; Nesbitt 2009: 299

235) Femur, proximal portion, anteromedial tuber: absent (0); present (1). Modified from Gauthier 1986; Benton 1999; Clark et al. 2000; Olsen et al. 2000; Benton and Walker 2002; Sues et al. 2003; Clark et al. 2004; Nesbitt 2009: 300

236) Femur, proximal portion, anteromedial tuber: small and rounded (0); large and "hooked" posteriorly (1). Modified from Gauthier 1986; Benton 1999; Clark et al. 2000; Olsen et al. 2000; Benton and Walker 2002; Sues et al. 2003; Clark et al. 2004; Nesbitt 2009: 300

237) Femur, proximal portion, anteromedial tuber offset medially (or posteriorly) relative to the posteromedial tuber: absent (0); present (1). Modified from Gauthier 1986; Benton 1999; Clark et al. 2000; Olsen et al. 2000; Benton and Walker 2002; Sues et al. 2003; Clark et al. 2004; Nesbitt 2009: 300

338) Femur, proximal portion, posteromedial tuber: present (0); absent (1). Modified from Novas 1996; Nesbitt 2005; Irmis et al. 2007; Nesbitt 2009: 301

339) Femur, proximal portion, posteromedial tuber: small (0); largest of the proximal tubera (1). Modified from Novas 1996; Nesbitt 2005; Irmis et al. 2007; Nesbitt 2009: 301

240) Femur, proximal portion, anterolateral tuber: present as an expansion (0); absent, the anterolateral face is flat (1). Sereno and Arcucci 1994; Irmis et al. 2007; Nesbitt 2009: 302

241) Femur, medial articular surface of the head in dorsal view: rounded (0); flat/straight (1). Nesbitt 2009: 303

242) Femur, ventral to the proximal head: smooth transition from the femoral shaft to the head (0); notch (1); concave emargination (2). Sereno and Arcucci 1994; Novas 1996; Nesbitt 2009: 304

243) Femur, femoral head orientation (angle with respect to the transverse axis through the femoral condyles Parrish 1986): anterior (60 - 90o) (0); anteromedial (20 - 60o) (1); medial (0 - 20o) (2). Benton and Clark 1988; Hutchinson 2001a; Nesbitt 2009: 305

244) Femur, femoral head in medial and lateral views: rounded (0); hook-shaped (1). Sereno and Arcucci 1994; Irmis et al. 2007; Nesbitt 2009: 306

245) Femur, dorsolateral margin of the proximal portion: smooth (0); ridge (=dorsolateral trochanter of some) (1). Modified from Nesbitt 2009: 307

246) Femur, dorsolateral margin of the proximal portion: ridge (=dorsolateral trochanter of some) is sharp (0); ridge (=dorsolateral trochanter of some) is rounded (1). Modified from Nesbitt 2009: 307

247) Femur, anterior trochanter (=M. iliofemoralis cranialis insertion): absent (0); present (1). Modified from Bakker and Galton 1974; Gauthier 1986; Novas 1992; Juul 1994; Novas 1996; Benton 1999; Langer and Benton 2006; Nesbitt 2009: 308

248) Femur, anterior trochanter (=M. iliofemoralis cranialis insertion): forms a steep margin with the shaft but is completely connected to the shaft (0); forms a steep margin with the shaft and separated from the shaft by a marked cleft (1). Modified from Bakker and Galton 1974; Gauthier 1986; Novas 1992; Juul 1994; Novas 1996; Benton 1999; Langer and Benton 2006; Nesbitt 2009: 308

249) Femur, medial articular facet of the proximal portion: rounded (0); straight (1). Nesbitt 2009: 309

250) Femur, anterolateral side of the femoral head: smooth, featureless (0); ventral emargination present (1). Sereno and Arcucci 1994; Irmis et al. 2007; Nesbitt 2009: 310

251) Femur, anterior trochanter shelf proximal to the fourth trochanter (insertion site for the M. iliofemoralis externus): absent (0); present (1). Gauthier 1986; Rowe and Gauthier 1990; Novas 1992; 1996; Langer and Benton 2006; Nesbitt 2009: 311

252) Femur, posterolateral portion (= fossa trochanterica, = posterolateral depression, = facies antitrochanterica articularis) of the head: level with the greater trochanter (0); ventrally descended (1). Novas 1996; Nesbitt 2009: 313

253) Femur, proximal surface: rounded and smooth (0); groove (1). Modified from Ezcurra 2006; Nesbitt 2009: 314

254) Femur, transverse groove on proximal surface: straight (0); curved (1). Modified from Ezcurra 2006; Nesbitt 2009: 314

255) Femur, fourth trochanter: present (0); absent, no distinct ridge for the attachment of the M. caudifemoralis (1). Modified from Gauthier 1986; Benton and Clark 1988; Sereno 1991; Juul 1994; Bennett 1996; Benton 1999; Nesbitt 2009: 316

256) Femur, fourth trochanter shape: mound-like and rounded (0); a sharp flange (1). Modified from Gauthier 1986; Benton and Clark 1988; Sereno 1991; Juul 1994; Bennett 1996; Benton 1999; Nesbitt 2009: 316

257) Femur, fourth trochanter: symmetrical, with distal and proximal margins forming similar low-angle slopes to the shaft (0); asymmetrical, with distal margin forming a steeper angle to the shaft (1). Langer and Benton 2006; Nesbitt 2009: 317

258) Femur, angle between the lateral condyle and the crista tibiofibularis in distal view: obtuse (0); about a right angle (1). Parker and Irmis 2005; Nesbitt 2009: 319

259) Femur, infrapopliteal ridge between medial femoral distal condyle and crista tibiofibularis: absent (0); present (1). Smith et al. 2007: 304; Irmis 2008: 306

260) Femur, medial condyle of the distal portion: tapers to a point on the medial portion in distal view (0); smoothly rounded in distal view (1). Nesbitt 2009: 320

261) Femur, surface between the lateral condyle and crista tibiofibularis on the distal surface: smooth (0); deep groove (1). Nesbitt 2009: 322

262) Femur, bone wall thickness at or near mid-shaft: thickness/diameter >0.3 (0); thin; thickness/diameter <0.3, > 0.2 (1); very thin; thickness/diameter <0.2 (2). Nesbitt 2009: 323

263) Femur, distal condyles of the femur divided posteriorly: less than 1/4 the length of the shaft (0); between 1/4 and 1/3 the length of the shaft (1). Nesbitt 2009: 324

264) Femur, anterior surface of the distal portion: smooth (0); distinct scar orientated mediolaterally (1). Nesbitt et al. 2009; Nesbitt 2009: 325

265) Femur, crista tibiofibularis (fibular condyle of Sereno & Arcucci 1994): smaller or equal in size to the medial condyle (0); larger than the medial condyle (1). Nesbitt et al. 2009; Nesbitt 2009: 326

266) Femur, anteromedial corner of the distal end: rounded (0); squared off near 90° or acute >90° (1). Nesbitt et al. 2009; Nesbitt 2009: 327

267) Tibia, proximal portion, cnemial crest: absent or just a slight bump (0); present and straight (1); present arcs anterolaterally (2). Benton and Clark 1988; Juul 1994; Novas 1996; Benton 1999; Irmis et al. 2007; Nesbitt 2009: 328

268) Tibia, proximal surface: flat or convex (0); concave, the posterior condyles of the tibia are separated from the cnemial crest by a concave surface (the cnemial process is proximally expanded) (1). Nesbitt 2009: 329

269) Tibia, proximal surface of the lateral condyle: convex or flat (0); depressed (1). Nesbitt 2009: 330

270) Tibia, lateral (fibular) condyle of the proximal portion: offset anteriorly from the medial condyle (0); level with the medial condyle at its posterior border (1). Langer and Benton 2006; Irmis et al. 2007; Nesbitt 2009: 331

271) Tibia, lateral margin of the lateral condyle of the proximal portion: rounded (0); squared-off (1). Nesbitt 2009: 332

272) Tibia, lateral side of the proximal portion: smooth (0); dorsoventrally oriented crest present (=fibular crest) and extends from the proximal articular surface (1); dorsoventrally oriented crest present and clearly separated from the proximal articular surface (2). Gauthier 1986; Rauhut 2003; Nesbitt 2009: 333

273) Tibia, posterolateral flange of the distal portion: absent (0); present and nearly contacts or contacts fibula (1); present and extends well posterior to the fibula (2). Novas 1992; Juul 1994; Benton 1999; Langer and Benton 2006; Irmis et al. 2007; Nesbitt 2009: 334

274) Tibia, posterolateral flange of the distal portion: nearly contacts or contacts fibula (0); present and extends well posterior to the fibula (1). Modified from Novas 1992; Juul 1994; Benton 1999; Langer and Benton 2006; Irmis et al. 2007; Nesbitt 2009: 334

275) Tibia, posterolateral margin of the distal end: straight or convex (0); concave (1). Irmis et al., 2007; Nesbitt 2009: 335

276) Tibia, posterior face of the distal end: rounded surface (0); distinct proximodistally oriented ridge present (1).Nesbitt 2009: 336

277) Tibia, posterior side of the distal portion: smooth and featureless (0); dorsoventrally oriented groove or gap (1).Nesbitt 2009: 337

278) Tibia, lateral side of the distal portion: smooth/rounded (0); proximodistally oriented groove (1). Novas 1996; Nesbitt 2009: 338

279) Fibula, attachment site for the M. iliofibularis, location: near the proximal portion (0); near the mid point between the proximal and distal ends (1). Sereno 1991; Nesbitt 2009: 340

280) Fibula, proximal end in proximal view: rounded or slightly elliptical (0); mediolaterally compressed (1). Nesbitt 2009: 341

281) Fibula, anterior edge of the proximal portion: rounded (0); tapers to a point and arched anteromedially (1). Nesbitt 2009: 342

282) Fibula, proximal portion in lateral view: symmetrical or nearly symmetrical (0); posterior part expanded posteriorly (1). Nesbitt 2009: 343

283) Fibula, medial face of the distal portion: smooth (0); banked with an articular facet that articulates with the astragalus (1). Nesbitt 2009: 344

284) Fibula, ridge on the medial side of the proximal end and oriented anterodistally: absent (0); present (1). Smith et al. 2007: 315; Irmis 2008: 328

285) Fibula, deep groove on the medial side of the proximal portion: absent (0); present, medial side of fibula bears distinct deep fossa (1). Smith et al. 2007: 314; Irmis 2008: 327

286) Fibula, distal end in lateral view: angled anterodorsally (asymmetrical) (0); rounded or flat (symmetrical) (1). Nesbitt 2009: 345

287) Distal tarsal 4, transverse width: broader than distal tarsal 3 (0); subequal to distal tarsal 3 (1).Sereno 1991; Juul 1994; Benton 1999; Nesbitt 2009: 347

288) Distal tarsal 4, size of articular facet for metatarsal V: more than half of lateral surface of distal tarsal 4 (0); less than half of lateral surface of distal tarsal 4 (1). Sereno 1991; Novas 1996; Benton 1999; Nesbitt 2009: 348

289) Distal tarsal 4, posterior prong: blunt (0); pointed (1). Langer and Benton 2006; Nesbitt 2009: 350

290) Distal tarsal 4, medial side: without a distinct medial process present in the anteroposterior middle of the element (0); with a distinct medial process present in the anteroposterior middle of the element (1). Nesbitt 2009: 351

291) Distal tarsal 4, proximal surface: flat (0); distinct, proximally raised region on the posterior portion (=heel of Sereno and Arcucci 1994) (1). Nesbitt 2009: 353

292) Astragalus, dorsally expanded process on the posterolateral portion of the tibial facet: absent or poorly expanded (0); expanded into a distinct, raised process (=posterior ascending process of Sereno and Arcucci 1994a) (1). Sereno and Arcucci 1994; Nesbitt 2009: 355

293) Astragalus, anterior ascending flange (anterior process): absent (0); present (1). Modified from Gauthier 1986; Novas 1992; 1996; Benton 1999; Rauhut 2003; Nesbitt 2009: 356

294) Astragalus, anterior ascending flange (anterior process): less than the height of the dorsoventral height of the posterior side of the astragalus (0); greater than the height of the dorsoventral height of the posterior side astragalus (1). Modified from Gauthier 1986; Novas 1992; 1996; Benton 1999; Rauhut 2003; Nesbitt 2009: 356

295) Astragalus, anteroposterior breadth of the astragalar ascending process: wedge- shaped/blocky (0); plate-like/laminar (1). Smith et al. 2007: 323; Irmis 2008: 335

296) Astragalus, anterior hollow: shallow depression (0); reduced to a foramen or absent (1). Nesbitt 2009: 357

297) Articular facet for the astragalus of the calcaneum lies: completely medial to the fibular facet (0); partially ventral to the fibular facet (1). Parrish 1993; Nesbitt 2009: 358

298) Astragalus, proximal surface: lacks a marked rimmed and elliptical fossa posterior to the anterior ascending process (0); possesses a marked rimmed and elliptical fossa posterior to the anterior ascending process (1). Langer and Benton 2006; Nesbitt 2009: 359

299) Astragalus, anteromedial corner shape: obtuse (0); acute (1). Bonaparte 1976; Novas 1989; Sereno 1991; Juul 1994; Novas 1996; Benton 1999; Nesbitt 2009: 361

300) Astragalus, proximal articular facet for fibula occupies: more than 0.3 of the transverse width (0); less than 0.3 of the transverse width (1). Langer and Benton 2006; Nesbitt 2009: 362

301)Astragalus, posterior groove: present (0); absent (1). Sereno 1991; Nesbitt 2009: 363 302 included ? Mk1 (est.)

302) Astragalus, tibial facet: concave or flat (0); divided into posteromedial and anterolateral basins (1). Sereno 1991; Parrish 1993; Juul 1994; Benton 1999; Nesbitt 2009: 366

303) Astragalus-calcaneum, ventral articular surface: flat or slightly convex (0); concavoconvex with concavity on calcaneum (1); concavoconvex with concavity on astragalus (2). Sereno 1991; Nesbitt 2009: 368

304) Astragalus-calcaneum, articulation: free (0); co-ossified (1). Sereno and Arcucci 1994; Irmis et al. 2007; Nesbitt 2009: 370

305) Calcaneum, ventral articular surface for distal tarsal 4 and the distal end of the tuber: continuous (0); separated by a clear gap (1); separated by a gap with a ventral fossa (2). Nesbitt 2009: 371

306) Calcaneum, articular facets for the fibula and astragalus: connected by a continuous surface (0); separated (1). Nesbitt 2009: 372

307) Calcaneum, calcaneal tuber: present (0); absent (1). Gauthier 1986; Sereno 1991; Juul 1994; Benton 1999; Nesbitt 2009: 373

308) Calcaneum, calcaneal tuber, distal end: rounded and unexpanded (0); flared, dorsally and ventrally (1). Sereno 1991; Nesbitt 2009: 374

309) Calcaneum, calcaneal tuber, distal end: without dorsoventrally aligned median depression (0); with dorsoventrally aligned median depression (1). Parrish 1993; Benton 1999; Nesbitt 2009: 375

310) Calcaneum, calcaneal tuber, shaft proportions at the mid-shaft of the tuber: taller than broad (0); about the same or broader than tall (1); just short of twice the mediolateral width of the fibular facet (2). Sereno 1991; Parrish 1993; Juul 1994; Benton 1999; Nesbitt 2009: 376

311) Calcaneum, articular surface for the fibula: convex (0); convex and hemicylindrical shaped (1); concave (2). Sereno 1991; Parrish 1993; Juul 1994; Novas 1996; Gower 1996; Benton 1999; Nesbitt 2009: 378

312) Calcaneum, shape: proximodistally compressed with a short posterior projection and medial process (0); transversely compressed, with the reduction of these projections (1). Langer and Benton 2006; Nesbitt 2009: 379

313) Calcaneum, articular surfaces for fibula and distal tarsal IV: separated by a non- articular surface (0); continuous (1). Sereno 1991; Juul 1994; Benton 1999; Nesbitt 2009: 380

314) Metatarsus, configuration: metatarsals diverging from ankle (0); compact metatarsus, with metatarsals II-IV tightly bunched (at least half of the length) (1). Sereno 1991; Juul 1994; Benton 1999; Nesbitt 2009: 382

315) Longest metatarsal: shorter than 50% of tibial length (0); longer than 50% of tibial length (1). Sereno 1991; Juul 1994; Benton 1999; Nesbitt 2009: 383

316) Metatarsals, midshaft diameters: I and V subequal or greater than II-IV (0); I and V less than II-IV (1). Sereno 1991; Juul 1994; Novas 1996; Benton 1999; Nesbitt 2009: 384

317) Metatarsal I: reaches the proximal surface of metatarsal II (0); does not reach the proximal surface of metatarsal II and attaches onto the medial side of metatarsal II (1). Gauthier 1986; Rauhut 2003; Nesbitt 2009: 385

318) Metatarsal I, length, relative to length of metatarsal III: 0-84% (0); 85% or more (1). Sereno 1991; Benton 1999; Nesbitt 2009: 387

319) Metatarsal III, proximal end: does not back to the ventral side of metatarsals II and IV (0); backs metatarsals II and IV posteroventrally, resulting in a T-shaped proximal profile (1). Carrano et al. 2002; Tykoski 2005; Nesbitt 2009: 389

320) Metatarsal III: longer than metatarsal II (0); subequal to metatarsal II (1). Nesbitt 2009: 390

321) Metatarsal III, outline of the proximal articular surface: rectangular (0); hourglass- shaped (1). Smith et al. 2007: 335; Irmis 2008: 369

322) Metatarsal IV, distal articulation surface: broader than deep to as broad as deep (0); deeper than broad (1). Sereno 1999; Langer and Benton 2006; Nesbitt 2009: 391

323) Metatarsal IV, proximal portion, possesses an elongated lateral expansion that overlaps the anterior surface of metatarsal V: absent (0); present (1). Sereno 1999; Langer and Benton 2006; Nesbitt 2009: 392

324) Metatarsal IV length: longer than metatarsal II (0); subequal or shorter than to metatarsal II (1). Gauthier 1986; Nesbitt 2009: 395

325) Metatarsal V, dorsal prominence separated from the proximal surface by a concave gap: absent (0); present (1). Nesbitt 2009: 397

326) Metatarsal V, 'hooked' proximal end: present (0); absent, and articular face for distal tarsal 4 subparallel to shaft axis (1). Sereno 1991; Juul 1994; Benton 1999; Nesbitt 2009: 398

327) Metatarsal V, phalanges: present and has "fully" developed first phalanx (0); present and has a "poorly" developed first phalanx (1); without phalangies and tapers to a point (2). Gauthier 1984; Parrish 1993; Nesbitt 2009: 399

328) Pedal unguals: weakly mediolaterally compressed, rounded or triangular in cross- section (0); dorsolaterally compressed (1); strongly mediolaterally compressed, with a sharp dorsal keel (2). Sereno 1991; Nesbitt 2009: 400

329) Osteoderms, dorsal to the vertebral column: absent (0); present (1). Gauthier 1984; Benton and Clark 1988; Sereno 1991; Juul 1994; Bennett 1996; Dilkes 1998; Benton 1999; Nesbitt 2009: 401

330) Osteoderms, presacral, dorsal, anterior edge: straight or rounded (0); with distinct anterior process (1). Clark et al. 2000; Olsen et al. 2000; Benton and Walker 2002; Sues et al. 2003; Clark et al. 2004; Nesbitt 2009: 403

331) Osteoderms, presacral, paramedian: flat (0); with distinct longitudinal bend near lateral edge (1). Clark et al. 2000; Olsen et al. 2000; Benton and Walker 2002; Sues et al. 2003; Clark et al. 2004; Nesbitt 2009: 404

332) Osteoderms, covering the appendages (=appendicular osteoderms), at least in part: absent (0); present (1). Heckert and Lucas 1999; Nesbitt 2009: 405

333) Presacral osteoderms, dimensions: square shaped, about equal dimensions (0); longer than wide (1); wider than long (2). Nesbitt 2009: 407

334) Anterior bar located on the anterior edge of an osteoderm: absent (0); present (1). Heckert and Lucas 1999; Parker 2007; Nesbitt 2009: 408

335) Ventral carapace in the dorsal area: absent (0); present (1). Heckert and Lucas 1999; Nesbitt 2009: 409

336) Gastralia: absent (0); present (1). Modified from Nesbitt 2009: 412

337) Gastralia: form extensive ventral basket with closely packed elements (0); well separated (1). Modified from Nesbitt 2009: 412

338) Angle between the ascending and horizontal processes of the maxilla: 35°-50° (0); less than 35° (1); more than 50° (2). Tykoski, 2005

339) Medial wall of the antorbital fossa extends to the posteroventral corner of the antorbital fenestra: present (0); absent (1). Modified from Galton 1990; Witmer 1997; Carrano et al. 2002; Langer 2004; Ezcurra and Novas, 2007

340) The Medial wall extends back to the posteroventral corner of the antorbital fenestra and is: narrow, lower than the jugal process of the maxilla below the antorbital fossa (0); broad, deeper than the jugal process of the maxilla below the antorbital fossa (1). Modified from Galton 1990; Witmer 1997; Carrano et al. 2002; Langer 2004; Ezcurra and Novas, 2007

341) Lateral surface of maxillary antorbital fossa at the base of the ascending process, form: smooth or widely concave (0); with deep, large, and subcircular or oval blind pocket/s (1). Carrano et al. 2002

342) Lateral lamina of bone of the lacrimal: absent (0); present (1). Modified from Ezcurra and Novas 2007 in Ezcurra and Brusatte 2011

343) Lateral lamina of the lacrimal covering most of the bone: absent (0); present (1). Modified from Ezcurra and Novas 2007 in Ezcurra and Brusatte 2011

344) Lateral lamina of bone of the lacrimal: with no interruption of the lacrimal antorbital fossa and restricted to the posterior margin of the ventral ramus along its dorsoventral extension (0); only interrupting the lacrimal antorbital fossa near the proximal end of the ventral ramus and ventrally restricted to posterior margin of the ventral ramus (1). Modified from Ezcurra and Novas 2007 in Ezcurra and Brusatte 2011

345) Sublacrimal part of jugal, form: tapering (0); bluntly squared anteriorly (1); expanded (2). Modified from Rauhut 2003; Ezcurra and Novas 2007

346) Sublacrimal part of jugal with a well developed dorsally directed prong overlapping the lacrimal: present (0); absent (1). Modified from Rauhut 2003; Ezcurra and Novas 2007

347) .Angle between ascending process and longitudinal axis of the jugal: right, obtuse or slightly acute (0); less than 75°, with an ascending process strongly posterodorsally oriented (1). Ezcurra and Novas 2007

348) Ventral process of squamosal, form: tapering ventrally (0); keeps consistent depth ventrally or expanded anteroposteriorly ventrally (1). Rauhut 2003

349) Squamosal-quadratojugal contact: present (0); absent (1). Modified from Holtz 1994

350) Squamosal-quadratojugal contact: small contact between the bones (0); broad contact (1). Modified from Holtz 1994

351) Postorbital participation in the supratemporal fossa: present (0); absent (1). Sereno 1999

352) Posteroventral process of dentary, extent: extends further posteriorly than posterodorsal process (0); subequal in length to posterodorsal process or posterodorsal process extends further posteriorly (1). Sereno 1999

353) Angular reaches posterior end of mandible, precluding surangular from the ventral margin of the jaw in lateral view: absent (0); present (1). Tykoski 2005

354) Mandibular joint, position: approximately ventral to the quadrate head (0); significantly posterior to the quadrate head (1). Rauhut 2003

355) Anterior and mid-dorsal vertebral centrum, length: less than two times centrum height (0); more than two times centrum height (1). Sereno 1999; Ezcurra and Novas 2007

356) Posterior dorsal vertebrae, length: strongly shortened, centrum length less than 1.33 times the height of the anterior articular surface (0); relatively short, centrum length equal or more than 1.33 times the height of the anterior articular surface (1); significantly elongated, centrum length equal or more than two times the height of the anterior articular surface (2). Rauhut 2003; Tykoski 2005

357) Neural spines of posterior dorsal vertebrae, shape: broadly rectangular, as high as long or longer than high (0); high rectangular, significantly higher than long (1). Rauhut 2003

358) Postacetabular process, posterior expansion form: narrow with sub-parallel margins, approximatelly straight parasagitally, or slightly laterally expanded (0); strongly laterally expanded posteriorly (1). Molnar et al. 1990; Novas 1993; Rauhut 2003; Ezcurra and Novas 2007

359) Sharp longitudinal edge on the lateral surface of the distal end of tibia: absent (0); present (1). Ezcurra and Brusatte 2011

360) Diagonal tuberosity on the anterior surface of the distal end of tibia: absent (0); present (1). Ezcurra and Brusatte 2011

361) Anteriorly bowed diagonal tuberosity on the medial surface of the distal end of tibia: absent (0); present (1). Ezcurra and Brusatte 2011

362) Strong anterior projection of the medial condyle of the astragalar body relative to the lateral condyle: present (0); absent (1). Ezcurra and Brusatte 2011

363) Ventral margin of the astragalus in anterior/posterior view: deeply concave (0); only slightly concave or straight (1). Hunt et al. 1998

364) Shallow fossa on the medial surface of the astragalar body: absent (0); present (1). Ezcurra and Brusatte 2011

365) Co-ossified tibiotarsus: absent (0); present (1). Rowe and Gauthier 1990

366) Metatarsal I, length: equal or more than 50% of the length of metatarsal II (0); less than 50% of the length of metatarsal II (1). Gauthier 1986

**S1.2.1.**

*Erythrosuchus africanus* 0001000120000110001000000000000000000100000000000000010100-000000010000000000000-0000000000010000000000001000000000000110000000?00000-000-010?00-0000000000000???1000?001?000101?000?00??01??0???000?000-00010-00000000000??000000000001000--01000000-0-000010???0000000000000000-0000010000010000000-?00?00?0000000000?000000000001000?0--?--???01-00--1101010101001000001000

*Euparkeria capensis* 000100000000?0-00100000000100000000000000000100000000[0 1]00011000000010010000000000-0001000000000000000000011000000000000100000000000000-0010000001000000000000000001000?00000010000000000??010?000?0000000-00000-00000000000??000000000001000--01000100-0-00000-0000000000000000000-0000000?00000000000-?00?0000000000010?000000000000000010100001011-010011000000110??000001000

*Revueltosaurus callenderi* 000000002000012011010100102010100000000001001010001101200110000000111-11000000?110????0?001?001?000000001100000110000?111000000000100-001101000101000000000000??01001010000000110110?0???000?0?0?????000-00000-00000000000??0000000000000010001000100-0-00000-0000000000000010000-001000010000?000100-?00?0001101101021010?0000?000000??10012110-?010100100001??01???00000100?

*Aetosaurus ferratus* 10100000111000-010110011002010?00?10000000011010?0110120011011000??11-?1?00000??????1???001000110??21??1??00001000010?111?????????????????????????0?000000???00001?01010000000110110?00??00000???00000?[0 1]0000?0-0000000000???000?0000000?0010001000100-0-00000-0000000?00000?10?00-0010??0????0???????????????????1?1?21?????????0??0???210012110-2000100101???0110???000001000

*Arizonasaurus babbitti* ??????1????00??00??00011000000100???????000?100000000?00011000000010001100000000-011?010001?001?0000011011000000000000111000010?00000-000-10?000-0?11000100???00010?1010?????????????????????????????001000010-020000?00111001-1111100011?10000000100-0-0000100001000100?0??????????????????????????????????????????????????????????????0--?--???21-0???100???00?10110????????

*Effigia okeeffeae* 011000104??0?110???01?111000000000000000011?000120010???00-0?10000011-11010??????111?0?0001?001000?01001000101-????????11???000?01?0??110-1??000-111111011?01100?00?1001??0?10?1?000100??00?000??????101010000-021010?00111101-122100001??11000100??0-0-000110???101110000001?010-0010010100010000100-?0??00011011?1?11?10000001010100?10--?--?1000001001000???10000?000001000

*Batrachotomus* *kuperferzellensis* 2?0?000?100101000011001100000111101001001100110011000?000110000001111-110011000??01111110011101?0000011011000000000000111000000000000-00111100010000100010010000010?101000000011110??????????????????001000010-00000010011000010010100011010000000100-0-0000100001010000000010000-001010010000??????0-??1????110?101021?100000??000?10?2111?10?101010100110001000101100000100?

*Postosuchus* *kirkpatricki* 000100001000010011000000201011111010010011011100111001110121000001111-110?1100111011??1?001111110002011011000000000000111??00???????0-00??1?0000-0000000000??0???1011011001000111?00100??00???0??00000010?0010-000000????????01??1110001?010000000100-0-00000-0001010?00000010000-001011011000?????00-?01?0001102101111?10000001010110?0111?100??10100--2010010110011000001000

*Dromicosuchus* *grallator* 2000000?2000110111100000001001?1000010001111110?1?100111011110?????????????100??????????001?011?000?0210110000000000001?1????????0??0-000-?1???0-00?0000000?001-01011111001110011?00100?10???????????001010010-000100?00????001??????????010000000100-0-00100-0001010?00000010000-001001011000?????00-??1????11021011?1?11?00???????????1110100101010100100???01?1?210????????

*Eudimorphodon* *ranzii* 1110???010100??0?2000011000000??0??0?000???0?????0?00000?0-??1??????????????????????????10??0?100?000000?00?0001?0000?101????1???0000-000-?1?0?0-?00?????0?010000????110000110?0?001??????00000110?110?0-100????0?00?00000??000??0?0000001????????????0-?00???1-??????0?0??????0??????????????1?0???0-?1??1????1?01?????11?001?1??0110000--?--?1121-00--201?1-11000210000???0?

*Dimorphodon* *macronyx* 2100??1?201000-0?11?0011000000?????00000???010?0?00000??00-??0??????????????????????????101?20?00??00??0?00??001?0000?1?1????1?1???????00-?1??00-0?????????010???????110000110?????1?0???00000?110?1?000-100?0-00100?00000??000????0000?0110000000?00-0-00000-???001020000000?00??000?0??1?00?11000?0-?1??101??1??1?????110001?001??1?000--?--???21-00--??0?1-1???011000011000

*Lagerpeton* *chanarensis* ??????????????????????????????????????????????????????????????????????????????????????????????????????????????????????????????????????????????0???000000000??????????????????????????????????????????000-00000-00000000000??0000000000000110001100110-0-010?0-0100010?0010100?000-0000010100011100110-?1001011?1?01???2011100000000001200--?--?????????????????????210?0000001

*Dromomeron* *romeri* ?????????????????????????????????????????????????????????????????????????????????????????????????????????????????????????????????????????????????????????????????????????????????????????????????????????????????????????????????????????110001100110-0-01010-1-?0011101111000000-00000101?00??????10-?1001011?1?01???2?1???????????????0--?--????????????????????????0000010?

*Dromomeron* *gregorii* ?????????????????????????????????????????????????????????????????????????????????????????????????????????????????????????????????????????????????????????????????????????????????????????????????????????????????????????????????????????1100011001?0-1001010-0000011101111000000-0000?????00??????1?????????1????????????????????????????????????????????????????????000?????

*Marasuchus* *lilloensis* ??????????????????????????????????????????????????????????????????011-10000?0011000????0????????????????????????????????101000?000000-000-0?0000-0000000000000???1?????1000?101???0??????????????????000-00000-00100000010??1010000000101110000000100-1000110-00000?0?00001000000-0001010100001100101001001001?010000100111000000000012?0--?--???01-??????????????010000011000

*Eucoelophysis* *baldwini* ????????????????????????????????????????????????????????????????????????????????????????????????????????????????????????????????????????????????????????????????????????????????????????????????????????????????10?????????????????????0??1001-001??1010100010????0??1100010?100????????????????????????????????????????????????0?????????????????????????????????????????????

*Sacisaurus* *agudoensis* ???????????????010?00000000000????????????????????????????????????????????????????????????1?????0?101????????0101001110?1????????????????????????????????????????1?????1?????????????????????????????0011???0100?0000100?0??1?????????????1000001110101010001000000???1000100101100001???????????????????????????????????????????????????????????21-0??????????????010000?????

*Silesaurus* *opolensis* 0?0?000?20?000-0100000000000001?000?0???00?0100100000?100110001??0001-10100000110001?001001?00100112100101000010000101011110010000000-000-100000-0000000110000???110101100001010?000?00??0???????????001100001001000010010??101011000011101001-01110101010[0 1]01000000101100010010110000101110001?1???01001001010?0?01???2011110?00010101210--?--????1-0???100?????0001100001110?

*Eocursor* *parvus* ????????????????????????????????????????????????0?????????????????011-10100???????????????????1??100?001??10?00?1011?11?1????????0?00-01??0??00??10?001?1?0??????11????11?0?101??????????????????????0011100010020000?11????001??10?0021?1100000021010110001100110010?000020000?11010101?10??1??????10???????????????????11???000???????0--?--??????????????????1????0100???0?

*Pisanosaurus* *mertii* ????????????????11??01??0???????????????????????????????????????????????????????????????0?????????000001?012000?1111111?1?????0?0???0-000-???00?????????????????????????????????????????????????????????????0??????????0??????????????2??????????????????????????001??0000?00000100001011100?1?????010010?111??0?01???21111???0?01??????0--?--????1-??????????????12??0000010?

*Heterodontosaurus* *tucki* 00010000001110-11000010000000010000000000110100020000030010000101?011-1?1100001101???0??001101111?0[0 2]000100120001110011111100000000100-000-0?0001111?0011100001??111?0?1110011010001011000100001120000?01110000-02100011110??001??100002011???00???????110?010-0110010?00002000011001??0110???111000?10?10?1?1??1?01?????111100?00101?1200--?--?0-01-011-11000101000100?00???00

*Lesothosaurus* *dianosticus* 00010?0?300000-1110001000000001000000000011010000000003000-0101010011-10100000???1??10??001101111100000101120000101101111????00000100-000-0??000-1??0?111?0??1???110???1100?1000?01010???000?00??0000001110?01002100011110??001??1000021111000000210101100010-011?01000000200000110101?111?00?????0010?100111??0?01???21111100000101???00--?--???01-011-01100000000??010????00

*Scutellosaurus* *lawleri* ??0??0??????????1?0?01??000?????????????00??10??2???????00-??????0????????0??????1??????0?1?????110[0 2]0????1?????01011011?10???00?00??0-?00-0??00??00??0???00?00??11??0?000001101000???????0???????????0?1110001002?100111?????01??0?????1111000000?1?1011000?0-01100100000020000011010?01?10001?????0100100111????01???21110?????0??????01???000?????????110???????01?0?000100?

*Saturnalia* *tupiniquim* ????????????????????0????0?????????11??????????1???????????????????1?????????0???????????1?????????0?????????0001001001?1???0???0??10-000-1??000-0000000001001???1000?111000111??010?????????????????0011000010011000100110010100121002110100000021011100011100110011?000020000110110101110011111100100101111??0?01???2011110000011101?00--?--????????????????????020000001000

*Plateosaurus* *engelhardti* 0100101020010100120000010010001001111000001010010000003010-0001010011-10100000010101?20?0111?1100101000110001000101100111110000100110-000-100100-00000010010000011000?1110011110001010101011111120000001100000-0210001001100101001210021101000000210111000011001100110000020000010110101110001111100100101111??0?01???2011010000011101100--?--???21-011-0110??000001000000[0 1]000

*Efraasia* *minor* 0?001?101001010012?0000?000000100??11000001?1001????00??1??000????011-101000000101??1???011?01??000000?1?000?0001001001?11??010?00110-0?0-100000-0000000001001??110?0?1110011110001010?01011?111?000?001100000-02100010010??10100121002110100000021011100001100110011000002000001011010111000111110010010?111??0?01???2011010000011101100--?--?1121-011-01?0??00000000000???00

*Herrerasaurus* *ischigualastensis* 20010?001001010001000000000000?0000100000?101000000000300100001?1?011-111000000??1??1???001101100??00000?100?0000000001111000?0100110-010-1??00110000000001011??11?????111011010000011201100110130101001100000-0210001001110101011200020101000010210111000110-0110011?000020010010010101110011111100100101111??0?01???2011110000011101100--?--???01-011-2001??0101001000001[0 1]00

*Staurikosaurus* *pricei* ????????????????????????????????????????????????????????????????????????????????????????????????00000?00??00?0000000001?1???0????01?0-0??????000-000000?00101????????????????????????????????????????001100000-02100010011101010111?00?011???00002101010001110011001??000020010010000101110001??????????????????????????????????????????0--?--?????????????????0??0010000???0?

*Chindesaurus* *bryansmalli* ????????????????????????????????????????????????????????????????????????????????????????????????????????????????????????1?????0?????1100??1??00???00?0????100?????????????????????????????????????????0????0????2?0??100???????1??????????1000000210111000110-0110000?0000200110100001?????????????01001?11?1?????????????????????????????????????????????????????00?000000?0?

*Eoraptor* *lunensis* 00010?001001111001000000100000?0011111000?10100?00000130010000101?011-?1?0?00???????????001?011000?00000?????0000?00?0101???00??001?0-??0-?00001100000?000?0?1??110?0?11110110100010111??1001110??101001100001012?00110011???010011000??11?????0??1?10100?0?????100????00020010?10000101?10??11111??100?0??1???0?0????2?11110000?1?1?1?00--?--?1100001000110???00?0110???00?00

*Tawa* *hallae* 1110000000111??0010000?0000000100??110000010100000001?3000-0?01?10011-11?000000??11?1001001?011000000000??00?0000000001?111?0111111110010-100??0-00000?000?011??111????1110110100000112001001101201010111000010120001100?110?0101?1???2??11010000220101000011101000111000020011010000101110001111100100101111??0?01???2111110000010101200--?--?1101-0???200101?0010110????1?00

*Coelophysis* *bauri* 1110111110111??0020000101100001?0101110000101001000011300100?01010011-111100001101???00?0011011000?00000?100?0000000001111100111111110100-100000-010001110101101111?0?111101101000101121110011113010111111110111211111001110101?11111021?11010000210111000111101001111000021011110110101110111110100100100111??1?01???21111110100101012?0--?--???101110010101-10001201?0000011

*Coelophysis* *rhodesiensis* 11101111111110-00200001111000010010111000010100100001?30010000111?011-1111000011011?1???0011011000020000??000000000000111110?1?11111101?0-100000-011001110100101111010111101101000101121110011113010211111110111211111001110100011111121011010000210111000111101001111000021011110110101110111110100100100111??1?01???2111111010010101200--?--???100110001101-1?00120101100111

*Panguraptor* *lufengensis* ???????????????00?0000??1101?0?0???1110???10?0?1000011300100?0??????????????????????????001?01?0???????0?1001??00000?0??11?0?1?11?11101?0-?00??0-0??00???????1???11??????????0??????11???????111?0?0??????????????111??????????????1?????1?????0?2?01110?01????????????0??21011?101?0101110??1?10100????0??11?????1???211111??1?0?0?0120?????????101110001110000???10?0???????

*Syntarsus* *kayentakatae* 1110111110111??002000010110100?00??1110?00101001000001300110?0????????0???00001101??1???001101100??00????10010000000001?1?100111111110100-?0?00???1??01111???101111?10111101?01??0?01????1?????12010?1?11?11011121??11001110101?1?111?21?110100002?011100011110100111100002101111011010110?111?1010010010?111??1??1????1111110?00101???0???????1100101011001000011?????0000011

*Camposaurus* *arizonensis* ????????????????????????????????????????????????????????????????????????????????????????????????????????????????????????????????????????????????????????????????????????????????????????????????????????????????????????????????????????????????????????????????????????????????101101???????1?????010010?111??1?01???211?????????????????????????????????????????????1111111?

*Liliensternus* *liliensterni* ???????????????00?0000??1100?0???????????????0?????01?3?0110??1110????????????????????????1?????000?0?????001000000000111???011??11010000-1??010-?0?00??10???1???1??101111011010001011???1???????????1111111011120011100111010101111??21?0101000021010110001110100011?000021011110110101110001?????0100100111??1??1???21111???000101???0??????????01????1001???1?101?0000000??

*Zupaysaurus* *rougieri* ???????????????0021000?0110100????11110100100?000000113010-000????0?1-11?00000????????????11011?0??000?0?10??0000?00?01??????1??????????????????????????????????????????????????????????????????????????????????????????????????????????????????????????????????????????????????101101????0??1?????0100100111??1??????2??????????????????????????00101011001011110????000000??

*Dilophosaurus* *wetherelli* 11101?11100?1??001100??00101001?0??11110??101000??00113000-000????011-1110001011010??2??001111?00000001001?010000000001111000011111111000-10?010-0000011101??1??111?1011110010100010112??1001111201011111110011121011100?110?01011111121?0101000021?1011000111010001110?0021011111110101110?11110100100100111??0?01???2111111000010101200--?--?11001010120111-?0110110000001?1

*Cryolophosaurus* *ellioti* ????????????????0?100???1???0??0??11111101100??000000?3000-?001?10?1??????00101?????0??????1111??????010?10?????0??0?01?1??????????111?00-100010-0?????1???0??????????????0???1?????????????????????????????011?21?11??0????1????111??????1010000210??110011110100011?0?00??????10??0????????1?????010?10?1????1??1???211??????????????????????11????101200???0?0100?0?0??0???

*Ceratosaurus* *nasicornis* 20001011000100-0010000100000001001111111001010012000013000-0?01?10011-1110000011011010??001111100??00000?10010000000001101001011111111000-101010-11??01111?110??10111011??????10???011???10???1??010?011111?01112??111000???1010??111?21?010100002100-1100010-0100111?000021000111110?01100100?????011110?111??1???????1110110100101?????????????0011101100101000100?01000001?

*Piatnitzkysaurus* *floresi* ???????????????001?000?00001001?????????00????????????????????????011-11100?111101???2?1??1??1?????????????????00000001?1?011?01111111000-1??110-01?0011111??1???1??1011110010100????????????????????0?11110011021111?00?1101???1111?121?010100002200-110001??0100011?000021011111110100110101?10100??1???????????????2??1??????1?????????????????000?????????????001000000?0?

*Allosaurus* *fragilis* 2100101120010101011?00010021001011111111001010000000013000-0001110011-111000111101??02??0011111000000010010010000000001111011101011111000-101110-00000111111110110101011110110100010112111101111?1??101111100110210111001101111021111121?010100002200-1100010-01000111000021011111110101110101110100111100111??0?01???2111011000110101200--?--?1100101012001010001001010000000

*Velociraptor* *mongoliensis* 01001011100100-1011100000101001001111111001010010000003100-0001110011-1?10011111011?????0011011000000000?1001000000000111??11?????????????10?01??01001111110?101101?1111100110100000012111000111?1??101111100110210111101110101?20010020101010010220??1100010-0100011100002101?111110?01?0010?11???011110?111??1?01????111011000010101200--?--?11000010111100100?00??000000000

*Daemonosaurus* *chauliodus* 200100?100011??000000000000?00?00?0010000?11????00?01030010??01?100???0????00000-1????????1?01?00?01?????1?0?0010000?01?1?00???11???100????01????????????????????????????????????????????????????????????????????????????????????????????????????????????????????????????????????????????????????????????????????????????????????????????????????21-011-0111000?00????????????

*Dracoraptor* *hanigani* 1110111?000000-00???0?11??1000????01110?????1????????1??010?????????????????????????1????0??0????????????????0?000?0001??????1??????1000???000???????????????1??????????????1?110??0??2??1000111?110?????????????????1001110?0101?01?12???100??002200-??00??0-???????2?????????1???????101?01?10010?1?10?????0???????????1?11?000101???00--?--?1?0??????011?????1??0??????????

**S1.3.1.**

Prior to running the final analysis the effect of atomizing compound characters (see S1.2.1.) was tested. The original matrix of You et al. (2014) (“unaltered” data set on the plots below) was compared to the larger matrix of this paper (see S1.2.1.) with *Daemonosaurus* and *Dracoraptor* excluded (“altered” data set on the plots below).

The two matrices were analysed using a manual jacknife procedure. One hundred replicates were run for each matrix with random numbers of random characters removed. The random characters were generated by a set of commands in Microsoft (MS) Excel. The replicates were run by typing “ccode!” (this resets the character list) into the TNT command line, followed by “ccode]” (this tells TNT to remove the following characters) and the list of characters generated by MS Excel. For each replicate the analysis was run using no weighting in a new technology search with default settings. The number of characters used per replicate and the resulting MPTs tree-lengths were recorded (MS Excel commands, the characters deleted in each replicate and resulting tree-lengths are available upon request SUV).

Two bivariate plots were generated from the recorded replicates. One bivariate examines the total number of characters per replicate *vs*. the tree-length, the other studies the number of characters removed *vs*. the difference in tree-length compared to the whole matrix. two predictions were made; 1) the first bivariate would demonstrate a decrease in tree-length relative to the number of characters in the new matrix; 2) the second bivariate would demonstrate the rate of decrease in tree-length as characters are removed is the same in each matrix.

The results of each bivariate demonstrated the predicted distributions. There was an overall reduction in character conflict, thus tree-length was relatively shorter with the additional characters.

The total tree-length of the MPTs recovered from the “unaltered” matrix is 1011 steps for 339 characters and 44 taxa, with a CI of 0.400 and RI of 0.700. The total tree-length of the MPTs recovered from the “altered” matrix is 1026 for 366 characters and 44 taxa, with a CI of 0.394 and RI of 0.700. The minimum possible tree-length for each matrix is 404, due to both using the same data. The maximum possible tree-length for the “unaltered” matrix is 2424, whereas for the “altered” matrix it is 2480. The first of the graphs above demonstrates that the “altered” matrix is ~60 steps shorter, relative to the number of characters when compared to the “unaltered” matrix. The second graph demonstrates that the general trend of the reduction in tree-length as characters are removed is at the same rate in both matrices, thus the observed difference is a product of atomizing compound characters and not the effect of the jacknife procedure.

Note that the plots presented here demonstrate a heteroskedastic distribution (Breusch-Pagan test = 0.000201 F significance testing the null hypothesis), consequently the regression values are not reported and little statistics can be performed on the graphs.

In conclusion, the utility of atomizing compound characters has been demonstrated. Despite not being able to re-code the matrix from direct observations due to limited resources, we consider this matrix to be far more robust than that of Nesbitt et al. (2009), Ezcurra and Brusatte (2011) and You et al. (2014). The RI values for each analysis are the same. The CI values are .006 lower in the “altered” dataset, but this is due to the effect of adding characters, while the minimum possible number of steps (404) remains the same. To resolve any human errors that may have been introduced by this practice implied weighting is used during the final analysis.

**S1.4.1.**

The analysis was run in TNT 1.1 (Goloboff *et al.* 2008), last updated February 2015, on a PC with an AMD FX 4350 (4.2 GHz Quad core) CPU and 8 GB (RAM) memory. The RAM available to the program was set to the maximum 1GB.

*Erythrosuchus africanus* was set as the outgroup. There were 46 active taxa and 366 characters. The matrix was run using implied weights with a K value of 3. In the first phase of tree searching a ‘new technology’ search was used to recover the shortest tree possible. To ensure the search was exhaustive, ratcheting was set to its maximum number of iterations. The settings are as follows:

Get trees from… Driven search (46 taxa at level 15) with initial additional sequences = 5

Find minimum length 1 time

Random seeds set to 11665 (using rseed* command)

Replace existing trees

Auto-constrain

Use… Sectorial search parameters:

RSS – Factor for number of selections = 43; Min. sector size = 23; Max. sector size = 23

CSS – Rounds = 3; Min. sector size = 10

For RSS and CSS globally swap every… 2 changes in sectors of size below 75; 10 changes in sectors of size above 75

For selections of size… above 75 use 6 drifting cycles; below 75 use 3 starts and fuse trees 0 times

Use… Ratchet parameters:

Stop perturbation phase when 20 substitutions made, or 99% swapping completed

Perturbation phase... 4 up-weighting probability, 4 down-weighting probability. Use alternate equal weights.

Number of iterations… 100,000 total number, 0 auto-constrained.

Use… Tree fusing parameters:

3 rounds, swapping after exchanging, start from best tree

When using driver… use fusing to multiply optimal trees

The second phase of tree searching used the ‘traditional search’ function to find the maximum number of MPTs. The search used trees saved to the RAM from the ‘new technology’ search as the starting trees. A TBR (Tree Bisection Reconnection) swapping algorithm was used to find the maximum topologies of the same length or shorter.

**S1.5.1.**

**Initial TNT new technology run**

Number of rearrangements tried = 35,469,085,232

1 tree saved

Tree length = 107.10053

CI = 0.378

RI = 0.681

**TNT traditional search**

Start swapping from1 tree, score 107.10053 steps

Number of rearrangements tried = 33,269

1 tree saved

Tree length = 107.10053

**S1.5.2.**

**
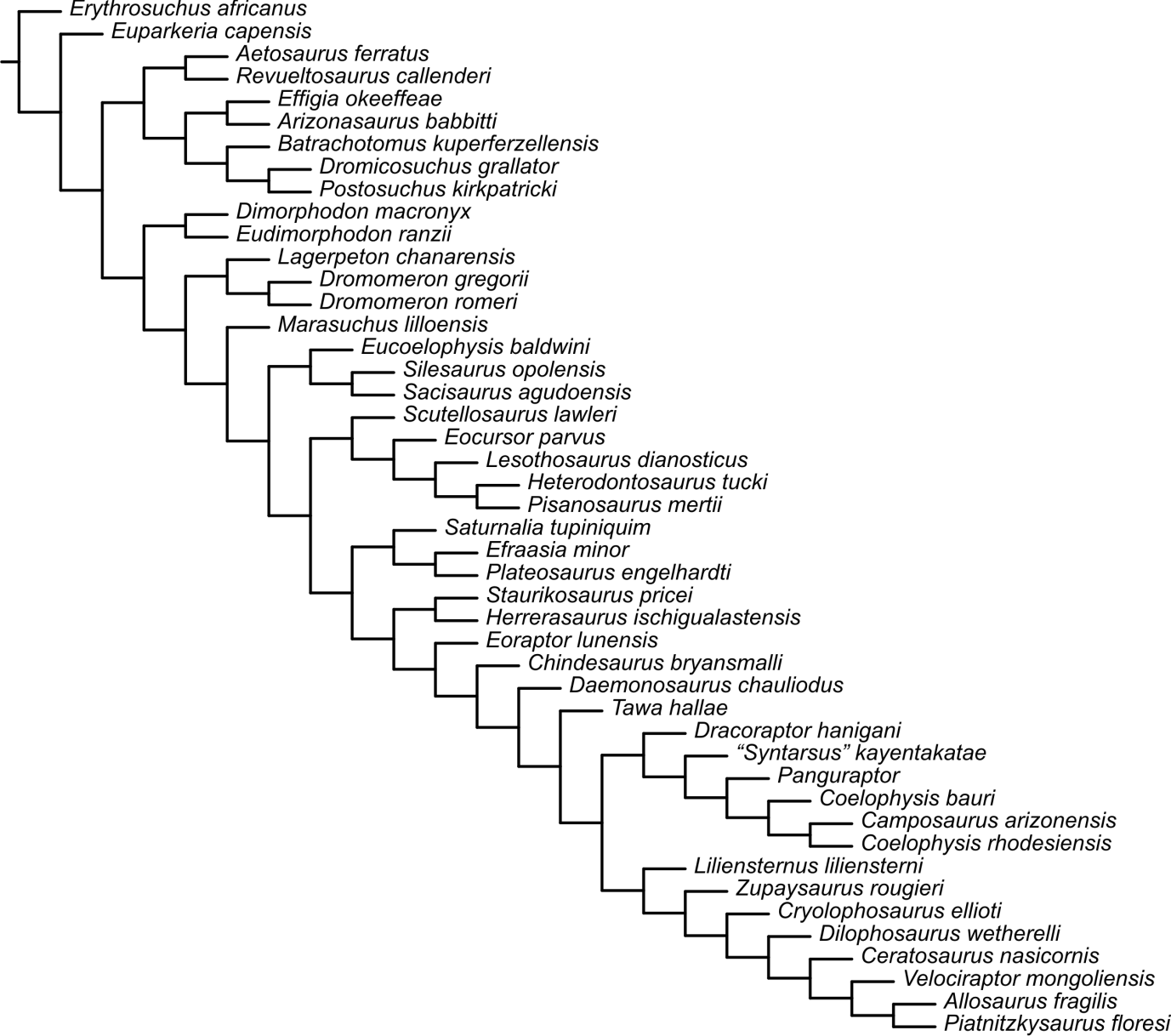
**

Single most parsimonious tree

**S1.5.3.**

**Synapomorphies/apomorphies at key nodes**

**Dinosauria**

Ch. 59: 1→0

Ch. 82: 0→1

Ch. 94: 0→1

Ch. 131: 0→1

Ch. 179: 0→1

Ch. 231: 1→2

Ch. 256: 0→1

Ch. 257: 0→1

Ch. 267: 1→2

Ch. 300: 0→1

Ch. 346: 0→1

**Neotheropoda**

Ch. 5: 0→1

Ch. 7: 0→1

Ch. 25: 0→1

Ch. 26: 0→1

Ch. 38: 0→1

Ch. 79: 0→1

Ch. 152: 0→1

Ch. 153: 0→1

Ch. 184: 0→1

Ch. 198: 0→1

Ch. 202: 0→1

Ch. 203: 0→1

Ch. 207: 0→1

Ch. 212: 0→1

Ch. 268: 0→1

Ch. 272: 0→1

Ch. 275: 0→1

Ch. 276: 0→1

Ch. 289: 1→0

Ch. 298: 1→0

Ch. 304: 0→1

Ch. 317: 0→1

Ch. 339: 1→0

*Dracoraptor hanigani*

Ch. 12: 1→0

Ch. 13: 1→0

Ch. 24: 0→1

Ch. 27: 0→1

Ch. 176: 0→1

Ch. 189: 1→0

Ch. 194: 0→1

Ch. 227: 1→0

Ch. 237: 1→0

Ch. 243: 1→2

Ch. 245: 1→0

Ch. 253: 1→0

Ch. 262: 1→2

Ch. 281: 1→0

Ch. 288: 1→0

Ch. 295: 0→1

Ch. 296: 1→0

Ch. 356: 1→0

**S1.5.4.**

Bootstrap supports were calculated for the single MPT, using 100 replicates in a new technology search with no ratchet. Absolute frequencies were recorded and are shown on the cladogram below.


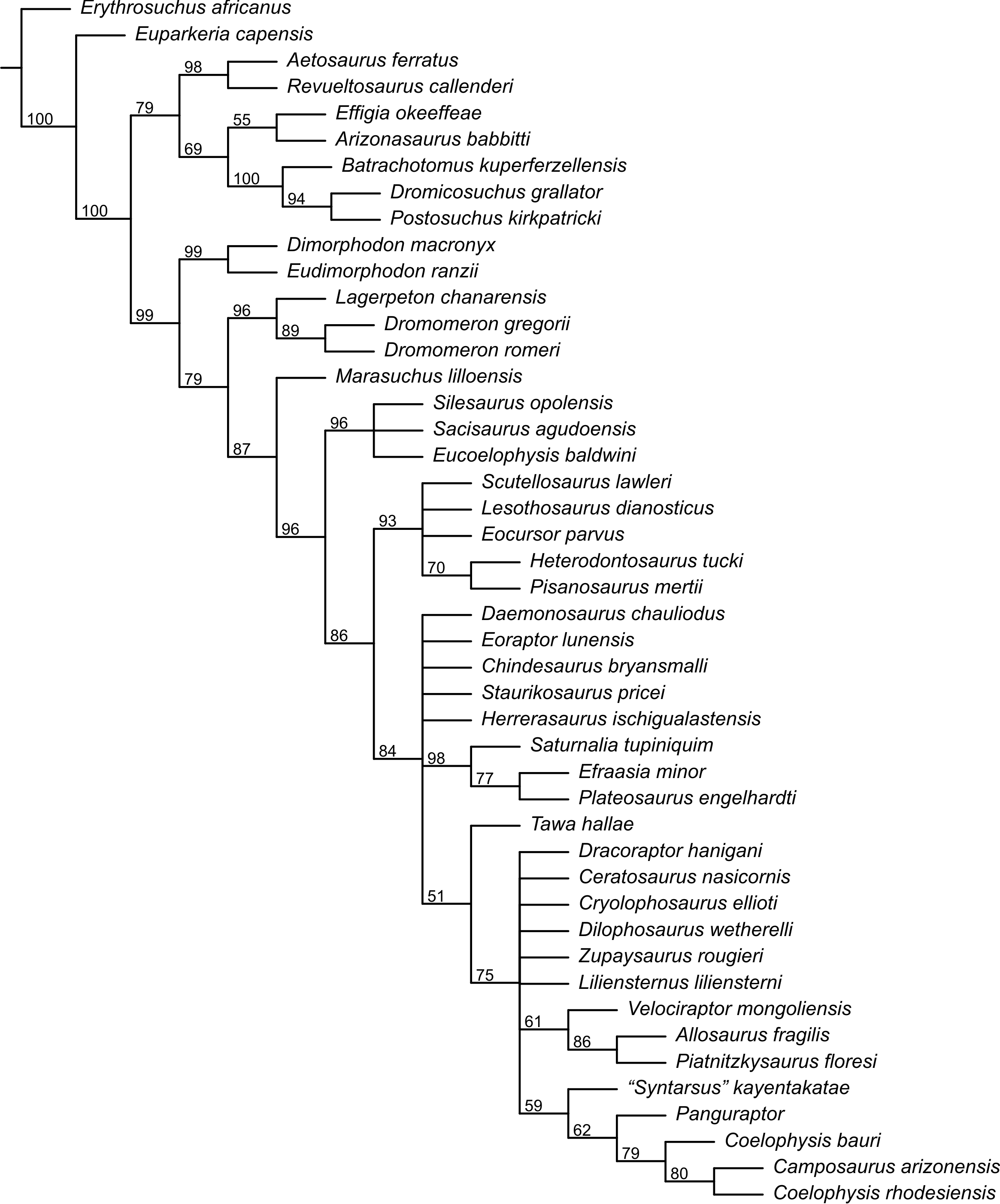


**References**

Brazeau MD (2011) Problematic character coding methods in morphology and their effects. Biological Journal of the Linnean Society 104: 489-498.

Ezcurra MD and Brusatte SL (2011) Taxonomic and phylogenetic reassessment of the early neotheropod dinosaur *Camposaurus arizonensis* from the Late Triassic of North America. Palaeontology 54: 763–772. doi:10.1111/j.1475-4983.2011.01069.x

Goloboff PA, Farris JS, Nixon KC (2008) TNT, a free program for phylogenetic analysis. Cladistics 24: 774-786.

Nesbitt SJ, Smith ND, Irmis RB, Turner AH, Downs A and Norell MA (2009) A complete skeleton of a Late Triassic saurischian and the early evolution of dinosaurs. Science 326: 1530–1533. doi:10.1126/science.1180350

Sues HD, Nesbitt SJ, Berman DS and Henrici AC (2011) A late-surviving basal theropod dinosaur from the latest Triassic of North America. Proceedings of the Royal Society B 278:3459-3464

You H-L, Azuma Y, Wang T, Wang Y-M and Dong Z-M (2014) The first well-preserved coelophysoid theropod dinosaur from Asia. Zootaxa 3873: 233–249. doi:10.11646/zootaxa.3873.3.3.
